# Tracking the catastrophic collapse of hybrid exciton-phonon order in a quantum material

**DOI:** 10.1038/s41467-026-75887-9

**Published:** 2026-07-30

**Authors:** Omar Abdul-Aziz, Danilo Comini, Johannes Lang, Nils Bartel, Michael Buchhold, Sebastian Diehl, Daniel Wolverson, Charles J. Sayers, Giulio Cerullo, Paul H. M. van Loosdrecht, Hamoon Hedayat

**Affiliations:** 1 II. Physikalisches Institut, Universität zu Köln, Köln, Germany; 2 Institut für Theoretische Physik, Universität zu Köln, Köln, Germany; 3 Institut für Theoretische Physik, Universität Innsbruck, Innsbruck, Austria; 4 Department of Physics, University of Bath, Bath, UK; 5 Dipartimento di Fisica, Politecnico di Milano, Milan, Italy; 6 Istituto di Fotonica e Nanotecnologie, Consiglio Nazionale delle Ricerche, Milan, Italy

**Keywords:** Phase transitions and critical phenomena, Ultrafast photonics, Electronic properties and materials

## Abstract

Revealing the hidden interactions that bind electronic and lattice components of cooperative quantum order is central to sculpting new states of matter. This challenge is epitomized by the charge density wave material 1*T*-TiSe_2_, where photoexcitation disrupts its presumed hybrid exciton-phonon order: the electronic component collapses within femtoseconds, while the periodic lattice distortion persists, challenging the definition of hybrid order. Here we resolve this paradox by uncovering a low-frequency mode (~0.13 THz) that emerges only in the ordered state and signals exciton-phonon coupling. This mode is consistent with a locked phason, a collective excitation arising when excitonic and lattice degrees of freedom share a coupled potential landscape. At a critical photoexcitation threshold, the collapse of the excitonic order flattens this potential, causing the locked phason to disappear, the charge density wave phonon to selectively overheat, and the surviving lattice distortion to become a trapped non-thermal remnant.

## Abstract (web summary)

Disentangling electronic and lattice orders and their coupling can be challenging. Here, the authors identify a low-frequency mode that is consistent with a locked phason and which signals the presence of exciton-phonon coupling, providing a signature of excitonic and lattice subsystem coupling.

## Introduction

In the landscape of quantum materials, the emergence of cooperative phases from the delicate interplay of electronic and lattice degrees of freedom is a central theme. Phenomena like charge density wave (CDW), unconventional superconductivity, and Mott insulating states are manifestations of this intricate coupling, where feedback between electron-phonon interactions and correlation-driven instabilities dictates the ground state^1,2^. A key frontier is therefore to transcend the static description of these phases, which, while well-defined, often conceals the complex hierarchy of interactions responsible for the phase stability. By leveraging intense ultrafast light-matter interactions, it is possible to break this equilibrium balance and drive a material into a non-thermal regime. The ultimate goal is to sculpt and control light-driven states of matter, transient configurations with emergent functionalities beyond the equilibrium limits, and establish a paradigm of materials through dynamic design^3,4^.

Quantum materials with coupled electronic and lattice orders provide a fertile ground for exploring complex many-body phenomena, where the hierarchy of interactions challenges conventional understanding and motivates advances in both theory and experiment, enabling the discovery of emergent phases under controlled perturbations. The layered transition metal dichalcogenide 1*T*-TiSe_2_ has long served as the prototypical testbed for this pursuit. Below a transition temperature of *T*_CDW_ ≈ 202 K, it hosts a commensurate CDW that simultaneously features a 2 × 2 × 2 periodic lattice distortion (PLD) and the opening of an indirect bandgap^5^. The fundamental origin of this phase has been the subject of a long and vigorous debate, with initial theories proposing a phonon-driven instability^6–9^ or pointing towards a purely excitonic insulator mechanism^10–15^. This dichotomy has now largely been resolved into the prevailing consensus that the CDW is a cooperative phenomenon^16,17^. In the equilibrium ground state, the excitonic and structural instabilities are not competing; rather, they are mutually reinforcing, locking the system into a hybrid order where the two components are inseparable. Yet, while this cooperative picture is widely accepted^18–22^, a direct experimental signature of the coupling itself has remained elusive.

**Fig. 1:**
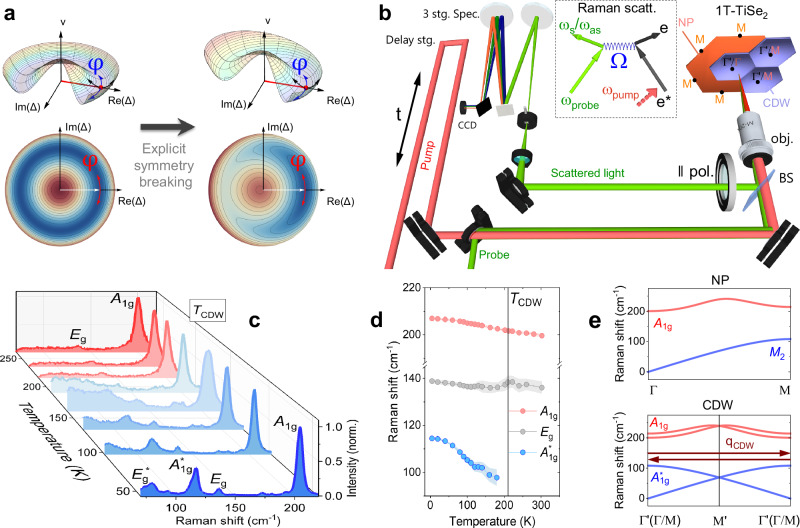
Raman access to coupled order in 1*T*-TiSe_2_. **a** Schematic free-energy landscape of the complex order parameter Δ = ∣Δ∣*e*^*iφ*^. Left: in the absence of locking, the potential has a Mexican-hat shape with a degenerate circle of minima in the $({{{\rm{Re}}}}\,(\Delta ),{{{\rm{Im}}}}\,(\Delta ))$ plane, and the phason corresponds to motion along the phase coordinate *φ*. Right: pinning to an external potential, e.g., exciton-phonon interactions, lifts this degeneracy, thereby turning the phason into a locked finite-frequency mode. **b** Overview of the experimental configuration and processes. Time-resolved Raman spectroscopy setup combining a femtosecond 750 nm pump with a time-delayed 513 nm probe in parallel backscattering geometry. Inset: Pump-probe Raman process; the pump pulse excites electrons, which relax by emitting phonons; the delayed probe detects Stokes (phonon creation) and anti-Stokes (phonon annihilation) photons. **c** Temperature-dependent Raman spectra under weak CW excitation at 532 nm. Below *T*_CDW_ ≈ 202 K, folded ${A}_{{{{\rm{1g}}}}}^{{{{\rm{*}}}}}$ and ${E}_{{{{\rm{g}}}}}^{{{{\rm{*}}}}}$ modes appear. **d** Evolution of phonon energies. The ${A}_{{{{\rm{1g}}}}}^{{{{\rm{*}}}}}$ mode hardens upon cooling, with ${A}_{{{{\rm{1g}}}}}^{{{{\rm{*}}}}}$ near 116 cm^−1^ at low temperature, serving as the primary phononic marker of the CDW. Shaded regions indicate fitting uncertainty of the extracted quantities. **e** Schematic phonon dispersions from DFT in the normal phase (NP, top) and in the CDW phase (CDW, bottom), showing only the branches relevant for the discussion. In the NP, the *A*_1g_ branch (red) is Raman active at *Γ*, while the soft *M*_2_ branch (blue) resides at the zone-boundary M point and is therefore not accessible in first-order Raman scattering. In the CDW phase, Brillouin-zone folding maps M onto the new zone centre $\Gamma^{{\prime}}$, where the former *M*_2_ branch appears as the folded ${A}_{{{{\rm{1g}}}}}^{{{{\rm{*}}}}}$ mode (blue). The emergence of ${A}_{{{{\rm{1g}}}}}^{{{{\rm{*}}}}}$ at $\Gamma^{{\prime}}$ in the Raman spectra is thus a direct fingerprint of the PLD.

To resolve this, it is essential to identify a spectroscopic observable that captures the coupling between the electronic condensate and the lattice. It is well established that the electronic gap oscillates at the frequency of the CDW phonon (${A}_{{{{\rm{1g}}}}}^{{{{\rm{*}}}}}$)^23–25^, yet this behavior alone does not constitute proof of a cooperative mechanism; such oscillations can also arise in a Peierls-like CDW, where the electronic order parameter follows the lattice distortion dynamics^26^. Following the experimental observation of exciton condensation in this system^27^, the key question now is whether the condensate exhibits its own intrinsic collective dynamics that actively couple to the lattice, thereby revealing the true cooperative nature of the order. The excitonic insulator phase is described by a complex order parameter Δ = ∣Δ∣*e*^*iφ*^, whose amplitude and phase reflect the magnitude and coherence of the electron-hole condensate. In the presence of a continuous U(1) symmetry, fluctuations of the phase *φ* cost negligible energy and correspond to a massless collective mode. In this context, the U(1) symmetry corresponds to the global phase of the electron-hole condensate, and the coupling to the CDW phonon explicitly breaks this continuous symmetry by fixing the relative phase between electronic and lattice components, thereby generating a pseudo-Goldstone mass for the phason, i.e., the locked phason (Fig. 1a)^28^. The phason frequency *ω*_phason_ thus serves as a direct spectroscopic fingerprint of the coupled exciton-phonon condensate and its symmetry-broken ground state. In CDWs driven by strong electron-phonon coupling, commensurability and strong bonding forces impose a rigid potential, producing a stiff locked phason with frequency comparable to that of the CDW phonon Ω, in the THz range^29,30^. By contrast, in a hybrid excitonic regime, the mass arises from comparatively weak exciton-phonon coupling, yielding a soft locked phason with *ω*_phason_ ≪ Ω^31–33^. This contrasts with systems such as rare-earth tritellurides, where Raman studies identified an axial Higgs mode rather than a soft locked phason^34,35^. Although low-frequency locked phasons have been observed in incommensurate CDW where continuous symmetry degeneracy is lifted by interactions such as impurities, interlayer coupling, or Coulomb forces^36–38^, their detection in a commensurate CDW system such as 1*T*-TiSe_2_ supports an excitonic-phononic hybrid origin^16,17,20^. This distinction renders the locked phason a spectroscopic fingerprint of cooperative exciton-phonon coupling.

The equilibrium picture of a unified, inseparable hybrid order is challenged when the system is driven far from equilibrium. A wealth of time-domain studies on 1*T*-TiSe_2_ have revealed a striking paradox^17,18,24,25,39–43^: Probes sensitive to electronic order, particularly time- and angle-resolved photoemission spectroscopy (TR-ARPES), report a collapse of the CDW gap within 100 femtoseconds^14^ after photoexcitation. In stark contrast, ultrafast diffraction experiments reveal that the superlattice peaks associated with the PLD persist for several picoseconds. Recent ultrafast low-energy electron diffraction studies interpreted the persistence of the PLD as evidence for a residual Peierls component of the CDW^18^. This subtlety suggests that both the excitonic and lattice components contribute but ultimately remain separable. In this view, once the excitonic condensate is disrupted by photoexcitation, a structural distortion remains, attributed to an underlying lattice-driven instability that survives independently. However, such an interpretation treats the excitonic and structural components as cooperative yet fundamentally distinct, challenging the notion of a truly hybrid order parameter and raising deeper questions about the physical origin and nature of the surviving PLD. While several observations of this transient decoupling have been made, the mechanism governing this phenomenon remains unresolved. Time-resolved Raman spectroscopy (TR-Raman) is ideally suited to address this, as it directly probes the dynamics of CDW phonons as well as the electronic susceptibility. Despite its proven sensitivity to non-equilibrium collective dynamics in correlated materials^44–46^, it has yet to be fully exploited to unravel the dynamics of CDW systems.

In this work, we resolve the paradox of hybrid order in 1*T*-TiSe_2_ by identifying a low-energy (~0.13 THz) collective mode that emerges exclusively in the ordered phase. Its appearance provides a spectroscopic signature of coupling between the excitonic and lattice subsystems. The frequency and non-equilibrium response of this mode are consistent with the behavior expected for a locked phason, arising from the coupling between the excitonic condensate and the CDW phonon. At a critical photoexcitation threshold, the collapse of the excitonic order parameter Δ flattens the coupled potential landscape, suppressing the restoring force for the CDW phonon and triggering a cascade of effects linked to an exciton-phonon catastrophe: phonon overheating, locked phason suppression, and the emergence of a non-thermal metastable state. The resulting phase, characterized by a saturated phonon response and the absence of the low-energy hybrid phason, is a dynamically trapped, non-thermal remnant of the PLD, whose nature is confirmed by a temperature evolution of the phonon component that diverges from the equilibrium CDW Raman response, indicating a distinct non-equilibrium trajectory. These findings establish a framework for disentangling and controlling cooperative quantum orders through targeted instabilities of a coupled potential landscape.

## Results

### Raman response of the CDW phonons in 1*T*-TiSe_2_

Figure 1b illustrates the TR-Raman experiment: a femtosecond 750 nm pump pulse photoexcites charge carriers, while a  ~1.2 ps and 513 nm probe pulse captures both Stokes and anti-Stokes Raman scattering in a parallel polarization scheme using a triple-stage spectrometer and CCD detector (see Methods and Suppl. Note 3). The inset illustrates the Raman process: the ultrafast photoexcitation promotes electrons across the indirect gap, creating a non-equilibrium carrier population that couples to a phonon of energy Ω. The time-delayed probe then drives a virtual electronic transition and is inelastically scattered as a Stokes (*ω*_0_ − Ω) or anti-Stokes (*ω*_0_ + Ω) photon, thus reading out the phonon population.

To establish a baseline for the coupled electron-lattice dynamics, we first map the equilibrium Raman spectrum of 1*T*-TiSe_2_ across its CDW transition. Figure 1c displays temperature-dependent Raman spectra obtained under weak continuous-wave excitation in a parallel polarization configuration. Above *T*_CDW_, only the zone-center *A*_1g_ and *E*_g_ modes of the high-symmetry phase are visible. Cooling below the transition activates two additional peaks, ${A}_{{{{\rm{1g}}}}}^{{{{\rm{*}}}}}$ and ${E}_{{{{\rm{g}}}}}^{{{{\rm{*}}}}}$ of the CDW phase.

In TR-Raman, to track the evolution of the CDW, we focus on the ${A}_{{{{\rm{1g}}}}}^{{{{\rm{*}}}}}$ phonon. The temperature dependence of the static Raman spectra (Fig. 1d) reveals that this mode emerges just below *T*_CDW_ at  ~ 95 cm^−1^ and hardens progressively to  ~116 cm^−1^ upon cooling, whereas the parent-phase *A*_1g_ mode remains only slightly affected^47^. First-principles phonon calculations (Fig. 1e and Suppl. Fig. S1) confirm that the ${A}_{{{{\rm{1g}}}}}^{{{{\rm{*}}}}}$ mode originates from the *M*-point phonon (labelled *M*_2_) of the normal phase, identified as the CDW soft mode^9,27^, which folds to the Brillouin-zone center ${\Gamma ^{{\prime}} }$ of the 2 × 2 × 2 superstructure (Fig. 1e). Since Raman spectroscopy probes only zone-center excitations, the appearance of this folded mode provides direct spectroscopic evidence of symmetry breaking and the formation of the PLD^48^. Accordingly, the TR-Raman measurements were performed in parallel (XX) polarization geometry to maximize sensitivity to the folded ${A}_{{{{\rm{1g}}}}}^{{{{\rm{*}}}}}$ mode (Suppl. Note 2). Crucially, the ${A}_{{{{\rm{1g}}}}}^{{{{\rm{*}}}}}$ phonon is not merely a marker of lattice distortion in Raman response. This CDW phonon strongly modulates the valence-band conduction-band hybridization amplitude^23^ and, by sharing the same wavevector *q*_CDW_, couples linearly to the excitonic condensate^27^. This interaction renders it a highly sensitive probe of the hybrid order in 1*T*-TiSe_2_, governed by the cooperative electronic and lattice dynamics.

### Critical transformation of the CDW phonon under photoexcitation

**Fig. 2:**
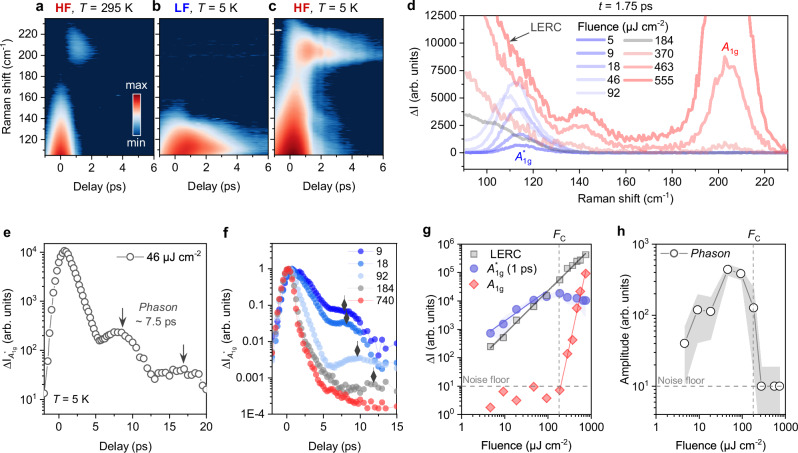
Critical transformation of the CDW phonon under photoexcitation. **a**–**c** Time-resolved Raman maps of the pump-induced differential intensity *ΔI*(*t*, *ω*) under three representative conditions, the same color scale and definition of *ΔI* apply to all three panels. **a** High fluence at 295 K: *ΔI* shows a strong low-energy Raman continuum (LERC) and population of the normal-phase *A*_1g_ near 200 cm^−1^. **b** Low fluence at 5 K: *ΔI* contains LERC and the folded CDW phonon $\Delta {I}_{{A}_{1g}^{*}}$. **c** High fluence at 5 K: the LERC and $\Delta {I}_{{A}_{1g}}$ dominate the spectrum, and $\Delta {I}_{{A}_{1g}^{*}}$ becomes spectrally obscured by the LERC. **d** Differential spectra *ΔI*(*ω*) at a fixed delay of 1.75 ps for fluences as indicated. $\Delta {I}_{{A}_{1g}^{*}}$ (blue) dominates at low fluence, weakens near the threshold range *F*_*c*_ ≈ 100–200*μ*J cm^−2^, and the normal-phase $\Delta {I}_{{A}_{1g}}$ (red) emerges abruptly above threshold, while the broadband *ΔI*_LERC_ increases steadily. **e** Time trace of the CDW mode $\Delta {I}_{{A}_{1g}^{*}}$ at 46 *μ*J cm^−2^ and 5 K, showing that the slowly decaying dynamics are modulated by a  ~7.5 ps (0.13 THz) oscillation (arrows), revealing the locked phason coupled to ${A}_{{{{\rm{1g}}}}}^{{{{\rm{*}}}}}$. **f** Dynamics of ${A}_{{{{\rm{1g}}}}}^{{{{\rm{*}}}}}$ for fluences as indicated, the oscillation amplitude decreases while its period increases, indicating locked phason softening (see diamonds). **g** Fluence dependence of the extracted intensities: *ΔI*_LERC_ grows superlinearly ( ∝ *F*^1.51±0.08^); $\Delta {I}_{{A}_{1g}}$ appears only above *F*_*c*_ and shows extreme nonlinearity ( ∝ *F*^6.99±0.40^); the amplitude of $\Delta {I}_{{A}_{1g}^{*}}$ initially scales linearly with fluence ( ∝ *F*^1.00±0.10^), but saturates as *F *≥ *F*_*c*_. **h** The locked phason amplitude grows only within a narrow low-fluence range and vanishes near *F*_*c*_, revealing a distinct behavior that identifies it as a separate collective excitation. Grey shaded region indicates the uncertainty range of the extracted phason amplitude, and the horizontal dashed line marks the noise floor.

The TR-Raman response of 1*T*-TiSe_2_ reveals that the hybrid order is highly susceptible to ultrafast perturbations, undergoing a qualitative transformation once the photoexcitation density exceeds a well-defined threshold. At room temperature (*T* >  *T*_CDW_), the sample is in the undistorted normal phase. Figure 2a shows that immediately after photoexcitation, a low-energy Raman continuum (LERC) at  ≲ 150 cm^−1^ rises and decays within the  ~1.2 ps pump-probe cross-correlation, followed by a transient population of the normal-phase mode near 200 cm^−1^, indicated by the change in its Raman intensity $\Delta {I}_{{A}_{1g}}$. The rise of the LERC mirrors the instrumental response function and resembles the fast sub-picosecond component in the time-resolved reflectivity (TRR) traces (Suppl. Note 11), pointing to ultrafast electronic energy redistribution rather than phonon population dynamics. While broad continua are known in equilibrium Raman spectra of other excitonic materials, such as Ta_2_NiSe_5_^49–51^, we avoid assigning this feature as a direct signature of the exciton condensate here. Although the LERC may be related to the excitonic fluctuations^51^, given its nearly invariant lineshape across conditions, we regard it as an electronic background channel (i.e., a change in the Raman susceptibility), and model it using an exponential function at each delay, which is then subtracted to provide the isolated phonon response (Suppl. Note 4).

At *T* = 5 K, there is a clear difference in the non-equilibrium response. For low fluences (LF; *F* ≲ 100 *μ*J cm^−2^), the dominant feature is a photoinduced change in the CDW phonon intensity $\Delta {I}_{{A}_{1g}^{*}}$, while the normal-phase phonon intensity remains unaffected by the pump. The LERC increases only modestly and, after approximately 1.5 ps, decays sufficiently to reveal a clean phononic spectrum.

In the high-fluence (HF) regime (*F* ≳ 200 *μ*J cm^−2^; Fig. 2c), the $\Delta {I}_{{A}_{1g}}$ response becomes visible, with an intensity even greater than that observed at 295 K, while the LERC intensifies and spectrally overwhelms $\Delta {I}_{{A}_{1g}^{*}}$, making the latter difficult to resolve. To quantify this crossover, we analyze the fluence dependence at a fixed delay of 1.75 ps (Fig. 2d). As *F* increases, the $\Delta {I}_{{A}_{1g}^{*}}$ intensity initially increases, but near *F* ≃ 187 *μ*J cm^−2^ its spectral weight above  ~ 100 cm^−1^ begins to diminish. Concurrently, the $\Delta {I}_{{A}_{1g}}$ associated with the normal phase exhibits a photoinduced increase starting at *F* ≳ 200 *μ*J cm^−2^. This transition becomes apparent only above a critical threshold *F*_*c*_ ≈ 100– 200 *μ*J cm^−2^. We note that this fluence range is consistent with the critical fluence range reported in prior ultrafast studies of 1*T*-TiSe_2_ (Suppl. Note 12). Based solely on this, one might speculate that the coexistence of an increasing $\Delta {I}_{{A}_{1g}}$ with a suppressed $\Delta {I}_{{A}_{1g}^{*}}$ reflects a photoinduced drift from the CDW landscape toward the normal phase; however, as shown below, this inference does not withstand scrutiny.

A central feature in the dynamics is a long-period modulation at  ~0.13 THz which modulates the ${A}_{1g}^{*}$ intensity (Fig. 2e and Suppl. Fig. S2), and is inconsistent with conventional phonon dynamics and points to a collective mode of the CDW with finite frequency, enabled by the hybrid exciton-phonon order (see Fig. 2f). We systematically rule out alternative scenarios. (i) A purely phononic origin is excluded, as no low-energy branches are found in either the normal or CDW phase, based on existing literature^52^ and our DFT calculations. A thickness-quantized acoustic breathing mode is also inconsistent with our observation; such a mode requires two free surfaces, whereas our bulk crystal does not provide the boundary conditions needed for a standing thickness resonance^53^. (ii) Beating between nearly degenerate phonons is inconsistent with both the fluence dependence of the oscillation and the absence of any such modes in our spectral window. (iii) A plasmonic origin is likewise implausible, as plasmon modes typically appear at much higher frequencies ( ≳ 1 THz)^17,27^. (iv) Phase stiffness governed by lattice pinning or impurity potentials fails to account for the observed behavior of the mode. In commensurate CDW systems, the energy scale of the phason is set by the Peierls pinning potential, i.e., comparable to bare lattice frequencies in the THz range. However, if the mode were of electron-phonon-driven, Peierls-type origin, photoexcitation would harden the phase mode toward its bare lattice frequency^54^, in stark contrast to the softening observed here. Moreover, the abrupt disappearance of the 0.13 THz mode at a fluence where the CDW phonon remains clearly visible further supports that it does not originate from lattice pinning and thus rules out a purely lattice-driven origin for the observed low-frequency oscillation. (v) Finally, instrumental artifacts or modulation of the Raman tensor are excluded, as they cannot generate a discrete frequency component with robust phase coherence and strong fluence-dependent behavior (Suppl. Notes 5–7).

Collectively, the evidence supports the interpretation of this mode as a locked phason of a hybrid exciton-phonon CDW. Several key observations reinforce this assignment. (i) The mode exclusively modulates the ${A}_{1g}^{*}$ phonon and is absent in other phononic or electronic dynamics (Suppl. Note 7). (ii) Its normalized amplitude, the ratio of the oscillation to the underlying ${A}_{1g}^{*}$ mode, remains constant for fluences *F* ≲ 20 *μ*J cm^−2^, a regime in which the excitonic condensate remains largely intact^17,24^, while the frequency of the oscillation softens continuously as the system approaches the critical threshold *F*_*c*_ (Fig. 2f, Suppl. Fig. S2). (iii) The mode vanishes abruptly at *F*_*c*_, even though the spectral weight and frequency of the ${A}_{1g}^{*}$ phonon persist well beyond this threshold. Although this combined evidence strongly supports the locked-phason interpretation, other exotic mechanisms cannot be fully ruled out. Throughout this work, we define *F*_*c*_ as the excitation fluence at which the locked phason amplitude collapses, marking the loss of coherent excitonic order while the lattice distortion remains.

Reports of locked phasons in other CDW systems^36,37^ support the presence of collective modes in the 0.1–0.15 THz range, but the fluence-dependent softening and abrupt disappearance observed here provide direct evidence of a hybrid character. We thus interpret the 0.13 THz oscillation as a locked phason of the coupled exciton-phonon order, i.e., a pseudo-Goldstone mode of the excitonic order which is pinned to the lattice. Its small gap arises from the breaking of the excitonic symmetry by phonon interactions, and its collapse at *F*_*c*_ reflects the loss of excitonic order, even as a metastable PLD remains. In the following sections, we demonstrate how such a locked phason emerges naturally in the effective theory of the exciton-phonon coupled potential.

To deconstruct the competing dynamics within the non-equilibrium state, we performed a quantitative analysis of the distinct components of the Raman response by fitting each spectrum with constrained Gaussians for the ${A}_{{{{\rm{1g}}}}}^{{{{\rm{*}}}}}$ and *A*_1g_ modes on top of an exponential LERC (for details of the analysis, Suppl. Note 4). The extracted fluence dependencies, shown in Fig. 2g, reveal that the electronic continuum, the normal-phase phonon, and the CDW phonon are governed by fundamentally different physical mechanisms. First, the photoinduced change of LERC intensity grows superlinearly as Δ*I*_LERC_ ∝ *F*^1.51±0.08^ (Fig. 2g). This non-integer exponent signifies a complex electronic response beyond simple single-photon absorption, likely involving a combination of non-thermal carrier-carrier scattering and a strong modulation of the electronic Raman susceptibility. Next, the changes in the normal-phase *A*_1g_ mode appear only above the critical threshold, and the amplitude rises with an exceptionally large exponent, $\Delta {I}_{{A}_{1g}}\propto \,{F}^{6.99\pm 0.40}$. Such strong nonlinearity is the hallmark of a highly cooperative or avalanche-like activation channel, which we attribute to a multiphonon-assisted carrier relaxation pathway across the residual partial gap, a scenario consistent with TR-ARPES observations of surviving gap features above *F*_*c*_^24,25^. For *F* ≲ 20 *μ*J cm^−2^, the change in the ${A}_{1g}^{*}$ amplitude scales linearly with fluence, $\Delta {I}_{{A}_{1g}^{*}}\propto {F}^{1.00\pm 0.10}$, consistent with a linear-response regime in which the CDW order is only weakly disturbed. Deviations from this behaviour appear only for *F* ≥ *F*_*c*_, where the ${A}_{1g}^{*}$ response saturates, signalling the onset of a distinct state in the hybrid exciton-phonon potential landscape (Fig. 2g). The persistence of this spectral weight, even in a regime dominated by the normal-phase mode, provides crucial evidence against a simple thermal melting of the CDW, which would require the CDW phonon to soften and disappear rather than saturate while retaining its spectral weight, supporting the survival of a PLD remnant in a non-equilibrium state. Finally, the amplitude of the locked phason exhibits a qualitatively different fluence dependence from all other observables: it grows only within a narrow low-fluence window, and then collapses to zero as *F* ≥ *F*_*c*_ (Fig. 2h). This distinct trend shows that the phason does not simply follow the ${A}_{{{{\rm{1g}}}}}^{{{{\rm{*}}}}}$ intensity or the electronic background, but instead represents a separate collective excitation.

### Selective overheating and saturation of the CDW phonon

**Fig. 3:**
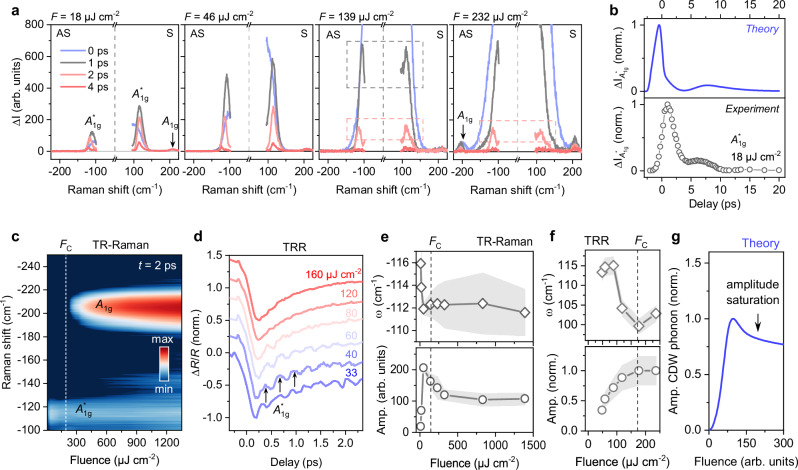
Selective overheating and saturation of the CDW phonon. **a** Time-resolved anti-Stokes (AS) and Stokes (S) Raman spectra at selected delays for increasing fluences. At *F* = 139*μ*J cm^−2^, the anti-Stokes and Stokes amplitudes of the ${A}_{{{{\rm{1g}}}}}^{{{{\rm{*}}}}}$ mode become nearly equal (dashed box), *I*_AS_ ≈ *I*_S_, indicating a strongly overheated, highly populated ${A}_{{{{\rm{1g}}}}}^{{{{\rm{*}}}}}$ phonon. Above *F*_*c*_, the ${A}_{{{{\rm{1g}}}}}^{{{{\rm{*}}}}}$ response saturates and an AS-active contribution from the normal-phase *A*_1g_ mode emerges. **b** Delay-dependent intensity $\Delta {I}_{{A}_{1g}^{*}}$ of anti-Stokes and Stokes signals from theory (top) and experiment (bottom) at *F* = 18 *μ*J cm^−2^. **c** Fluence dependence of the anti-Stokes spectra at t = 2 ps delay time. The ${A}_{{{{\rm{1g}}}}}^{{{{\rm{*}}}}}$ mode persists over all fluences, while *A*_1g_ emerges above *F*_*c*_. **d** Fluence-dependent time-resolved reflectivity (TRR) dynamics, dominated by ${A}_{{{{\rm{1g}}}}}^{{{{\rm{*}}}}}$ oscillations. Oscillations persist at all fluences, but become rapidly damped as *F* approaches *F*_*c*_. The dynamics are normalized to the initial transient reflectivity change, which increases with fluence, making the coherent ${A}_{{{{\rm{1g}}}}}^{{{{\rm{*}}}}}$ oscillations appear reduced, although their absolute amplitude increases and eventually saturates (Supplementary Note 11). **e** Anti-Stokes fits of ${A}_{{{{\rm{1g}}}}}^{{{{\rm{*}}}}}$ peak parameters versus fluence. Top: phonon frequency shows softening; bottom: amplitude saturates above *F*_*c*_. **f** TRR analysis of ${A}_{{{{\rm{1g}}}}}^{{{{\rm{*}}}}}$: both oscillation frequency and amplitude saturate near *F*_*c*_, consistent with Raman results. Grey shaded regions indicate the fitting uncertainties of the extracted parameters. **g** Theoretical model reproduces the saturation behavior of the ${A}_{{{{\rm{1g}}}}}^{{{{\rm{*}}}}}$ amplitude observed in the experiment.

To track the fate of the CDW phonon across the critical threshold, we turn to anti-Stokes TR-Raman, which allows for a more accurate determination of phonon occupation (Fig. 3). As the pump fluence approaches the critical range *F*_*c*_ ≈ 100-200*μ*J cm^−2^, the anti-Stokes signal of the CDW mode ${A}_{{{{\rm{1g}}}}}^{{{{\rm{*}}}}}$ increases and becomes comparable to its Stokes counterpart within ∼1 ps (Fig. 3a, dashed box), demonstrating strongly populated non-equilibrium state. Above *F*_*c*_, the anti-Stokes intensity of ${A}_{{{{\rm{1g}}}}}^{{{{\rm{*}}}}}$ saturates and shows no further increase, while the anti-Stokes response of the emergent normal-phase *A*_1g_ mode grows, signalling a crossover to a different dynamical regime.

To obtain a quantitative estimate of the phonon population after photoexcitation, we evaluate the detailed anti-Stokes to Stokes ratio *I*_AS_(*t*)/*I*_S_(*t*) and invert the Bose-Einstein relation (Suppl. Note 5, Eqs. 6,7) to extract the phonon occupation at frequency Ω and delay *t*, *n*(Ω, *t*), and the corresponding effective phonon temperature *T*(*t*). Well below *F*_*c*_, the maximum *T*(*t*) remains moderate, around 100-200 K (Suppl. Fig. S3). As *F* approaches *F*_*c*_, ${T}_{{A}_{1g}^{*}}$ rises steeply and exceeds 10^3^ K, far above the equilibrium *T*_CDW_. Crucially, even at these extreme temperatures, a clear CDW phonon peak ${A}_{{{{\rm{1g}}}}}^{{{{\rm{*}}}}}$ remains in the Raman spectrum (Fig. 3a), demonstrating that the phonon is still present and that the PLD survives overheating of the CDW mode. The temporal profile of *T*(*t*) shows a delayed rise and a slow oscillatory tail (Suppl. Fig. S3). A fit with two exponential decays plus a damped cosine function (Suppl. Note 6, Eq. 8) yields a low-frequency mode near 0.13 THz (Suppl. Fig. S3). In contrast, the time-dependent Raman susceptibility decays exponentially on a  ~ 1.5 ps timescale and shows no detectable modulation (Suppl. Fig. S3). The coherent modulation of the ${A}_{{{{\rm{1g}}}}}^{{{{\rm{*}}}}}$ mode persists on the anti-Stokes side (Fig. 3b), and the effective theory closely reproduces its dynamics. The effect is more pronounced than on the Stokes side (Suppl. Fig. S3), suggesting that this long-period mode interacts with a genuine phonon contribution rather than an electronic response. This mode appears with a cosine-like phase in the phonon sector (frequency of anti-Stokes ${A}_{{{{\rm{1g}}}}}^{{{{\rm{*}}}}}$ and effective phonon temperature ${T}_{{A}_{1g}^{*}}$ shown in Suppl. Fig. S3b, c), consistent with a hybrid excitonic-phononic character and a rapid displacive quench of the potential: photoexcitation changes the minimum set by the excitonic order on an ultrafast timescale, and the locked phason corresponds to coherent phase oscillations around this new minimum.

The fluence-delay map of the anti-Stokes signal in Fig. 3c shows that, while the normal-phase *A*_1g_ response strengthens dramatically above *F*_*c*_, the CDW phonon ${A}_{{{{\rm{1g}}}}}^{{{{\rm{*}}}}}$ remains clearly visible at all fluences, demonstrating that the underlying PLD is not completely melted, but survives at least partially even in the high-fluence regime. Gaussian fits to the anti-Stokes lineshape reveal that, above *F*_*c*_, the ${A}_{{{{\rm{1g}}}}}^{{{{\rm{*}}}}}$ frequency and amplitude approach plateau values (Fig. 3e): the mode neither softens further nor gains additional population. Any possible chiral refinement of the CDW order is not expected to alter this interpretation, since the collapse of the low-frequency mode together with the survival of the folded ${A}_{{{{\rm{1g}}}}}^{{{{\rm{*}}}}}$ response is not explained by a small symmetry refinement affecting mainly folded *E*_*g*_ mode features^55^. To independently probe the coherent dynamics, we performed TRR measurements across a broad fluence range and compared the results with anti-Stokes TR-Raman. The reduced oscillation visibility in Fig. 3d at high fluence stems from normalization to the large initial step and increased damping (Suppl. Note. 11). The reflectivity oscillations, matching the ${A}_{{{{\rm{1g}}}}}^{{{{\rm{*}}}}}$ frequency, show the same fluence dependence as the anti-Stokes Raman signal: amplitude saturation and frequency softening above *F*_*c*_, with no evidence of phonon disappearance. By isolating the oscillatory component from the background decay and fitting the resulting signal, we extracted fluence-dependent amplitude and frequency trends (Fig. 3f), which closely mirror the frequency-domain results.

Taken together, these observations reveal that the CDW mode does not melt away, but remains as a well-defined folded CDW phonon whose coherence is strongly suppressed once the critical regime is reached. Experimentally, this manifests as (i) a simultaneous saturation of the anti-Stokes ${A}_{{{{\rm{1g}}}}}^{{{{\rm{*}}}}}$ intensity and the phonon occupation *n*(Ω), such that *I*_AS_(*t*) ≈ *I*_S_(*t*) while the ${A}_{{{{\rm{1g}}}}}^{{{{\rm{*}}}}}$ peak itself remains clearly visible, and (ii) a saturation of the intensity of the CDW mode above *F*_*c*_, verified by both TR-Raman and TRR: additional photoexcitation energy neither enhances the coherent oscillation amplitude nor further increases the phonon population. Instead, the ${A}_{{{{\rm{1g}}}}}^{{{{\rm{*}}}}}$ mode persists as a well-defined CDW phonon with essentially fixed spectral weight and frequency, while its phase-coherent motion is largely quenched and the PLD survives in an overheated, metastable configuration. This saturation is a key signature of metastability: once the coherent hybrid coupling collapses at *F*_*c*_, additional absorbed energy is redirected into other relaxation channels rather than further enhancing the ${A}_{{{{\rm{1g}}}}}^{{{{\rm{*}}}}}$ response, therefore, the CDW mode remains visible, but its response no longer grows.

This behaviour is captured by our effective theory (see Methods, Effective theory), where the CDW phonon amplitude itself saturates as a function of pump strength (Fig. 3g), in line with the experimentally observed saturation of the TR-Raman anti-Stokes population (Fig. 3e). We emphasize that the effective model is designed to capture the symmetry structure and qualitative evolution of the coupled exciton-phonon potential, rather than to predict absolute frequencies or microscopic parameters, and therefore, its role is to elucidate the origin of the observed soft mode and the collapse of phase stiffness under increasing fluence. In the model, the amplitude phonon couples linearly to the excitonic order parameter Δ, and a finite Δ value at low fluence stabilizes a Mexican-hat potential for the hybrid exciton-phonon order. The coupling to the phonon explicitly breaks the continuous phase symmetry of the excitonic condensate, giving rise to a finite-frequency pseudo-Goldstone excitation, the locked phason. As the fluence approaches the critical threshold *F*_*c*_, the collapse of Δ gradually deforms the potential landscape and suppresses coherent phase oscillations, thereby removing the locked phason and reducing the restoring force that sustains the coupled dynamics. Experimentally, this corresponds to a saturation of both the anti-Stokes population and the coherent amplitude (Fig. 3f, g), marking the onset of a regime dominated by incoherent phonon excitation.

### Fingerprints of a dynamically trapped non-thermal state

**Fig. 4:**
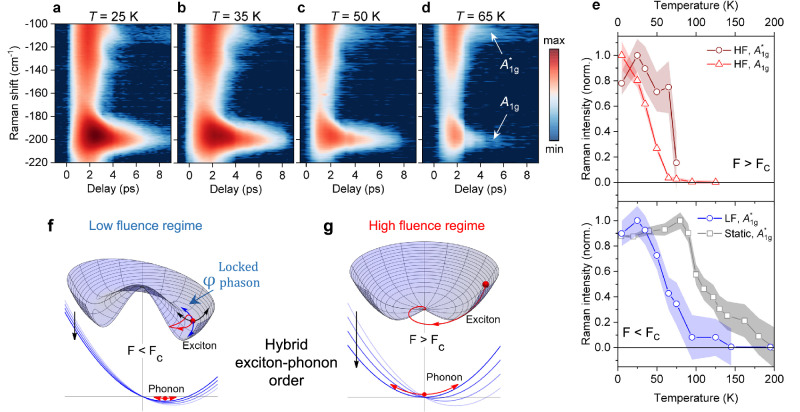
The high-fluence regime constitutes a non-thermal, dynamically trapped phase. **a**–**d** Anti-Stokes TR-Raman maps of the pump-induced differential intensity *ΔI* at a fixed fluence of *F* = 405*μ*J cm^−2^ for base temperatures of 25, 35, 50, and 65 K, the same color scale and definition of *ΔI* apply to all four panels. The photoinduced normal-phase *A*_1g_ mode ( ~ 200 cm^−1^, bottom arrow) weakens with increasing *T* and  is strongly reduced by 65 K, whereas the folded CDW mode ${A}_{{{{\rm{1g}}}}}^{{{{\rm{*}}}}}$ ( ~ 116 cm^−1^, top arrow) persists. **e** Temperature dependence of normalized Raman intensities. Top: In the high-fluence regime (*F* > *F*_*c*_), both *A*_1g_ (red triangles) and ${A}_{{{{\rm{1g}}}}}^{{{{\rm{*}}}}}$ (red circles) collapse near 65 K. Bottom: In the low-fluence regime (*F* < *F*_*c*_), the ${A}_{{{{\rm{1g}}}}}^{{{{\rm{*}}}}}$ intensity (blue circles) tracks the equilibrium static CDW order parameter (grey squares). Shaded regions indicate fitting uncertainties of the extracted parameters. **f**, **g** Schematic of the coupled exciton-phonon potential. For simplicity, the excitonic potential is shown statically, and the red arrow indicates the trajectory of the order parameter after the quench. **f** Low-fluence regime (*F* < *F*_*c*_) where the tilted Mexican-hat potential remains intact, supporting coherent oscillations of the locked phason. **g** High-fluence regime (*F* > *F*_*c*_) where the potential flattens, trapping the system in a metastable remnant PLD distinct from the thermal state.

Finally, we demonstrate that the high-fluence regime above *F*_*c*_ constitutes a distinct, dynamically trapped non-equilibrium phase, one that cannot be accessed via thermal pathways. To probe this regime, we perform temperature-dependent TR-Raman at a fixed fluence well above *F*_*c*_. The results reveal a strikingly non-thermal behavior: the intensity of the photoinduced *A*_1g_ mode, i.e., an excitation fingerprint of the normal phase, is strongest at low temperatures and systematically weakens as the base temperature approaches *T*_CDW_ (Fig. 4a–d). This trend is counterintuitive: in a thermally driven transition, one would expect the normal-mode population to grow with temperature, not decrease. Even at 65 K, the CDW mode remains comparable in strength to the *A*_1g_ (white arrows, Fig. 4d), highlighting their distinct dynamical origins.

This anomalous temperature dependence implies that a hidden, non-thermal parameter governs the evolution, most plausibly, the underlying electronic structure. This interpretation is consistent with the temperature dependence of the LERC. As established by TR-ARPES, the electronic gap in 1*T*-TiSe_2_ remains partially open even at high fluence^24,25,56–58^, supporting the persistence of a correlated phase. As the base temperature rises, this gap narrows, reducing the density of states available for phonon-mediated relaxation and potentially suppressing electron-phonon coupling due to the evolving lattice and band structure at the Brillouin zone boundary. In this picture, as the gap closes at higher temperatures, the relaxation channel weakens, explaining the suppression of the photoinduced increase in *A*_1g_ intensity with increasing *T*. The system thereby enters a regime where a normal-mode response is strongest at the lowest temperature, signifying a trapped out-of-equilibrium phase stabilized by electronic structure constraints. The contrasting temperature dependences of *A*_1g_ and ${A}_{{{{\rm{1g}}}}}^{{{{\rm{*}}}}}$ therefore reflect distinct non-equilibrium channels rather than simple thermal activation of one mode over the other.

To confirm that the high-fluence state represents a distinct non-equilibrium phase, we contrast phonon evolution with the melting behavior observed at low fluence (Fig. 4e). At low fluence, the ${A}_{{{{\rm{1g}}}}}^{{{{\rm{*}}}}}$ intensity persists up to  ~ 100 K, tracking a non-thermal melting pathway that accelerates relative to the equilibrium transition (Supplementary Note 9). The dynamics change entirely under high fluence. Here, the ${A}_{{{{\rm{1g}}}}}^{{{{\rm{*}}}}}$ intensity deviates sharply from this trend, vanishing abruptly around 65 K in stark contrast to the BCS-like suppression^59^ and the observed evolution for an equilibrium order parameter. This anomalous behavior provides direct evidence for a non-thermal state and suggests that the excitonic component of the coupled order parameter has been annihilated. Meanwhile, the amplitude of the *A*_1g_ mode weakens gradually as the base temperature increases, diminishing to a constant, room-temperature-level background by  ~ 65 K. This strong quenching is consistent with the highly nonlinear excitation mechanism of this mode (Fig. 2g); as the system heats up, the progressive thermal closure of the residual CDW gap reduces the density of photoexcited carriers available for multiphonon relaxation. A simple laser-induced heating cannot account for the transition at 65 K^60^ (Suppl. Note 10), reinforcing the non-thermal nature of this dynamically trapped state.

## Discussion

Our results demonstrate that the paradox of disentangling cooperative CDW order in 1*T*-TiSe_2_ under strong drive is not merely a delay of the lattice response, but the catastrophic collapse of a genuinely hybrid exciton-phonon order. By isolating a collective low-frequency mode, accessed through the CDW phonon and identified as a locked phason of the coupled order parameter, we obtain a direct spectroscopic signature of the shared potential landscape linking excitonic and structural degrees of freedom. This 0.13 THz mode emerges from the hybrid potential and acquires a finite frequency through breaking of the excitonic *U*(1) symmetry by the CDW phonon. We note that the observation of the soft locked phason reflects the phase dynamics of the hybrid order and does not by itself quantify the relative energetic contributions of excitonic versus lattice components.

Tracking the locked phason across the critical excitation threshold allows us to probe the transformation of the exciton-phonon potential. Our effective theory shows that this mode is stabilized by the excitonic order parameter Δ, and that its suppression marks the collapse of the coupled potential. This drives the saturation of the ${A}_{1g}^{*}$ amplitude. Beyond this point, coupled excitonic-phononic dynamics vanishes: energy is transferred to incoherent phonon excitations, leaving a structurally ordered but excitonically depleted state.

The persistence of this structural distortion after the loss of excitonic order warrants further scrutiny. Recent ultrafast low-energy electron diffraction experiments have similarly reported a *saturation* of PLD that survives at high fluence, consistent with our observations^18^. While that study attributes the remaining distortion to a dominant electron-phonon driven (Peierls-like) component of the equilibrium CDW, our detection of the soft locked phason challenges this interpretation. In our picture, the PLD survives not as a purely lattice-stabilized order, but as a dynamically arrested remnant. As the optical drive suppresses Δ, the hybrid potential flattens, removing the restoring force for the locked phason and leading to the saturation of the CDW phonon response (Fig. 3e, g). The lattice remains stranded in a non-thermal configuration due to inertial, anharmonic, and dissipative constraints that prevent structural recovery. This mechanism demonstrates how non-equilibrium drives can be used to dismantle complex quantum orders by dynamically reshaping the potential that binds their constituents.

These findings establish a route for quantum control: the dismantling of cooperative phases through nonlinear excitation. This mechanism offers a plausible connection to the light-induced dimensional crossover recently reported in this and other layered materials^4,25,40,43^. In particular, the 3D-to-2D CDW crossover in 1*T*-TiSe_2_ has been linked to the breakup of bound electron-hole pairs^40^. The exciton-phonon catastrophe identified here provides a clear fluence-dependent pathway for annihilating the excitonic component of the order, potentially triggering the loss of interlayer coherence. These results suggest that direct manipulation of the low-frequency hybrid locked phason, such as via resonant excitation, could enable coherent control over the dimensionality of electronic order in layered quantum materials. Looking ahead, the strategy established here should be transferable to a broad class of correlated systems, using ultrafast optical drive as a practical design tool for non-equilibrium quantum phases.

## Methods

### Time-resolved Raman spectroscopy (TR-Raman)

Time-resolved spontaneous Raman measurements were performed in fs pump and ps probe configuration. A 750 nm laser pulse  (~ 0.3 ps) was used as the pump to impulsively excite the sample, while a time-delayed 513 nm laser pulse  (~ 1.2 ps) was used to record the Stokes and anti-Stokes Raman spectra in parallel polarization geometry. The time resolution of the setup was approximately 1.2 ps, defined by the cross-correlation between pump and probe pulses^61^. A mechanical beam blocker cut the scattering below 100 cm^−1^ to enable detection of the relevant low-energy phonon modes. The probe fluence was kept low to ensure a non-perturbative measurement, and the sample was held at cryogenic temperatures in a vacuum cryostat throughout all measurements.

### Effective theory

TiSe_2_ undergoes a second-order transition at *T*_*c*_ ≈ 202 K into a commensurate 2 × 2 × 2 charge-density wave (CDW) with a weak trigonal lattice distortion. Experimental and theoretical studies point toward a cooperative origin involving both excitonic condensation and a periodic lattice distortion (PLD).

To describe the ultrafast photoexcitation dynamics in 1*T*-TiSe_2_, we employ a minimal model consisting of two electronic bands (valence and conduction) and a single optical phonon mode. Electrons and phonons are itinerant, but their mutual interactions are local in real space. This approximation rests on three physical arguments: (i) Coulomb screening renders the effective electron-electron interaction short-ranged, (ii) the low-temperature CDW corresponds to an equal-weight superposition of the three symmetry-equivalent *M*-point phonons, so one effective phonon mode (${A}_{{{{\rm{1g}}}}}^{{{{\rm{*}}}}}$) suffices (this is consistent with the literature, e.g., shown in DFT simulations^52^), and (iii) the lattice distortion couples predominantly to on-site charge transfer between Ti and Se orbitals.

#### Hamiltonian

The total Hamiltonian

1 $$\begin{array}{r}H={H}_{{{{\rm{el}}}}}+{H}_{{{{\rm{ph}}}}}+{H}_{{{{\rm{el-ph}}}}}+{H}_{{{{\rm{el-el}}}}}\end{array}$$

comprises electronic, phononic, and interaction terms. Electrons in valence and conduction bands (*i* = 1, 2) have dispersions *ϵ*_*i*_(**k**) in the Brillouin zone, while phonons correspond to a single local mode of frequency Ω at each site *n*:

2 $${H}_{{{{\rm{el}}}}}={\sum}_{{{{\bf{k}}}},i}{\epsilon }_{i}({{{\bf{k}}}})\,{\hat{c}}_{{{{\bf{k}}}},i}^{{{\dagger}} }{\hat{c}}_{{{{\bf{k}}}},i},\qquad {H}_{{{{\rm{ph}}}}}=\frac{1}{2M}{\sum}_{n}({\hat{P}}_{n}^{2}+{M}^{2}{\Omega }^{2}{\hat{X}}_{n}^{2}),$$

where ${\hat{X}}_{n}$ and ${\hat{P}}_{n}$ are the displacement and conjugate momentum of site *n*.

The precise form of these interactions is irrelevant; only their symmetry properties matter. The simple choices made here simplify the derivation of the equations of motion. Assuming short-range interactions, the electron-phonon and screened Coulomb terms are local and of Fröhlich, respectively, Hubbard form:

3 $${H}_{{{{\rm{el-ph}}}}}=g{\sum}_{n,ij}{\hat{X}}_{n}\left({\hat{c}}_{n,i}^{{{\dagger}} }{\hat{c}}_{n,j}+{{{\rm{h.c.}}}}\right),$$

4 $${H}_{{{{\rm{el-el}}}}}=V{\sum}_{n,ij}{\hat{c}}_{n,i}^{{{\dagger}} }{\hat{c}}_{n,j}^{{{\dagger}} }{\hat{c}}_{n,j}{\hat{c}}_{n,i}.$$

Here *g* is the on-site electron-phonon coupling, and *V* > 0 the screened electron-electron repulsion. The latter favors local electron-hole pairing between valence and conduction states, promoting excitonic condensation.

The complex order parameter

5 $${\Delta }_{n}=V{\sum}_{ij}\langle {\hat{c}}_{n,i}^{{{\dagger}} }{\hat{c}}_{n,j}\rangle$$

quantifies the exciton condensate, which induces hybridization between the two bands. We assume that in the CDW phase, Δ_*n*_ and the lattice displacement *X*_*n*_ share the same modulation wavevector: phonons connect the valence-band maximum at *Γ* with the conduction-band minima at the symmetry-equivalent *M* points, so a static distortion at momentum **q**_*i*_ = *Γ* → *M*_*i*_ induces an excitonic gap with the same periodicity.

In thermal equilibrium, all *M*-points are symmetry equivalent. Here we assume that this also holds after photo-excitation, i.e., that the pump couples equally to all *M* points, and hence only the uniform component of the coupled order is dynamic. We mention that this approach also excludes the effect of a collective, coherent rotation through different *M*-points, which might emerge from an anisotropic excitation of the material.

We extend the time-dependent mean-field theory that was successfully used in the related material Ta_2_NiSe_5_^31,62^ to the study of TiSe_2_: we apply a mean-field decoupling to *H*_el_ − ph and *H*_el_ − el and assume homogeneous mean fields Δ_*n*_ → Δ(**q**_*ℓ*_) ≡ Δ and $\langle {\hat{X}}_{n}\rangle \to \langle \hat{X}({{{{\bf{q}}}}}_{\ell })\rangle \equiv X$ for all components with **q**_*ℓ*_ being the vector pointing from *Γ* to *M*_*ℓ*_. Moving to the reduced Brillouin zone, this yields

6 $${H}^{{{{\rm{MF}}}}}={\sum}_{{{{\bf{k}}}},ij}\left[{\epsilon }_{i}({{{\bf{k}}}}){\delta }_{ij}-(\Delta+gX){\tau }_{ij}^{x}\right]{\hat{c}}_{{{{\bf{k}}}},i}^{{{\dagger}} }{\hat{c}}_{{{{\bf{k}}}},j}+g\,{{{\rm{Re}}}}(\Delta ){\sum}_{n}{\hat{X}}_{n}+{H}_{{{{\rm{ph}}}}},$$

where *τ*^*x*^ acts in the valence-conduction subspace. The combination Δ + *gX* forms an effective hybridization gap: a finite Δ induces *X*, and vice versa.

#### Time-dependent mean-field dynamics

To model ultrafast pump-probe dynamics, we promote Δ(*t*) and *X*(*t*) to time-dependent fields. Neglecting spatial variations, the system is described by a (0 + 1)D time-dependent Ginzburg-Landau theory (cf. ref. ^31^). Implementing a canonical relaxation mechanism  ~ *κ*, *γ* driving the system back towards the stationary state yields the equations of motion

7 $${\partial }_{t}^{2}X=	 -\gamma {\partial }_{t}X-{\Omega }^{2}X-2g\Omega \,{\mbox{Re}}\,\left(\Delta \right)\,\\ i{\partial }_{t}\Delta=	 (1+i\kappa (t))\left\{\left[-m(t)+U| \Delta {| }^{2}\right]\Delta+2{g}^{{\prime} }X\right\}\,.$$

The phonon frequency Ω has been measured to high precision with X-ray scattering^63^. The effective mass *m* and anharmonicity *U* are obtained from integrating over particle-hole fluctuations. Within this framework, phonon-phonon scattering is included phenomenologically through the lattice damping term *γ*, while *κ*(*t*) describes relaxation in the excitonic sector. The damping term *γ* accounts for the observed decay and linewidth broadening of the ${A}_{{{{\rm{1g}}}}}^{{{{\rm{*}}}}}$ response. here, the photon flux and the collective response of the electron fluctuations enter via the time-dependence in *m*(*t*), *κ*(*t*)

8 $$m(t)=	 {m}_{0}-F{e}^{-{t}^{2}/{\tau }^{2}}-\eta (t),\\ 	 \kappa (t)={\kappa }_{0}\left(1+\eta (t)\right).$$

It includes the pump and a convolution of the pump with an exponential decay $\exp (-t/{\tau }_{th})\theta (t)$. The term *η*(*t*) represents an effective Hartree term generated by the fluctuations generated during the quench

9 $$\eta (t)=F{\eta }_{e}{e}^{\frac{{\tau }^{2}-4t{\tau }_{th}}{4{\tau }_{th}^{2}}}\left(\,{\mbox{erf}}\,\left(\frac{t}{\tau }-\frac{\tau }{2{\tau }_{th}}\right)+1\right).$$

Within this minimal model, the coupled dynamics of Δ(*t*) and *X*(*t*) capture the coherent evolution of excitonic and phononic order observed in time-resolved Raman and reflectivity measurements. Without loss of generality, we set *m*_0_ = 1. The other fixed parameters are $\Omega=\frac{37}{10}*2\pi$ (which translates to  ≈ 3.5 THz by setting the time unit to 1 *ps*), *η*_*e*_ = 1/40, *U* = 1, *τ* = 1/10, *g* = − 10, ${g}^{{\prime} }=-1/10$, *γ* = 1/2, *κ* = 1/3, *τ*_*th*_ = 3.

#### Collective excitations and locked Phason

The excitonic-phononic order in 1*T*-TiSe_2_ breaks a discrete rotational symmetry rather than a continuous one. This symmetry breaking is governed by the electron-phonon coupling *g*, which is small compared to both Ω and the electron-electron interaction. Consequently, the phase (phason) mode of the exciton acquires a small mass term $\propto \sqrt{g{g}^{{\prime} }}$, producing a weakly gapped collective excitation, the *locked phason*.

Linearizing the equations of motion around equilibrium yields three collective modes: (i) a high-frequency amplitude (*Higgs*) mode, (ii) a hybridized phonon mode dominated by the PLD, and (iii) a low-frequency locked phason with ${\omega }_{{{{\rm{phason}}}}}\propto \sqrt{g{g}^{{\prime} }}\ll \Omega$. This slow mode naturally explains the few-picosecond oscillations observed experimentally.

### Density functional theory

First-principles calculations were carried out using the Vienna Ab initio Simulation Package (VASP)^64,65^. The structural coordinates for the commensurate 2 × 2 × 2 CDW phase of 1*T*-TiSe_2_ (24 atoms) were taken from the GGA-vdW relaxed structure reported in ref. ^52^, with one further relaxation step using the GGA-PBE exchange-correlation functional and projector augmented-wave (PAW) potentials. Van der Waals interactions were included using Grimme’s D2 correction^66^. Electronic smearing was applied with a broadening of 0.08 eV (effective *T* ~ 900 K), and results were checked down to *T* ~ 100 K. Phonon properties were computed using Phonopy^67^ with a 2 × 2 × 2 supercell (192 atoms) based on the CDW unit cell. A kinetic energy cutoff of 300 eV and a 6 × 6 × 3 Monkhorst-Pack *k*-point grid were used^68^.

## Supplementary information

Supplementary Information

Peer Review file

## Data Availability

The source data supporting the main and Supplementary Figs. are available in Zenodo at 10.5281/zenodo.20395573. The repository includes a README file mapping each figure panel to the corresponding data files.

## Code availability

The density-functional-theory calculations were performed using VASP, and phonon properties were computed using Phonopy and UPHO. The corresponding input files and workflow commands are available in Zenodo at 10.5281/zenodo.20395573. Scripts used for the effective theory simulations are available from the corresponding authors upon request.

## References

[1] Rowe, E. et al. Resonant enhancement of photo-induced superconductivity in K_3_C_60_. *Nat. Phys.* **19**, 1821–1826 (2023).

[2] Maklar, J. et al. Nonequilibrium charge-density-wave order beyond the thermal limit. *Nat. Commun.* **12**, 2499 (2021). 33941788 10.1038/s41467-021-22778-w PMC8093280

[3] Yusupov, R. et al. Coherent dynamics of macroscopic electronic order through a symmetry breaking transition. *Nat. Phys.* **6**, 681–684 (2010).

[4] Domröse, T. et al. Light-induced hexatic state in a layered quantum material. *Nat. Mater.* **22**, 1345–1351 (2023). 37414945 10.1038/s41563-023-01600-6 PMC10627829

[5] Traum, M., Margaritondo, G., Smith, N., Rowe, J. & Di Salvo, F. Tise_2_: Semiconductor, semimetal, or excitonic insulator. *Phys. Rev. B* **17**, 1836 (1978).

[6] Williams, P. Phase transitions and charge density waves in the layered transition metal dichalcogenides. In *Crystallography and crystal chemistry of materials with layered structures*, 51–92 (Springer, Dordrecht, 1976).

[7] Hughes, H. Structural distortion in TiSe_2_ and related materials-a possible Jahn-Teller effect? *J. Phys. C: Solid State Phys.* **10**, L319 (1977).

[8] Rossnagel, K., Kipp, L. & Skibowski, M. Charge-density-wave phase transition in 1T-TiSe_2_: Excitonic insulator versus band-type Jahn-Teller mechanism. *Phys. Rev. B* **65**, 235101 (2002).

[9] Weber, F. et al. Electron-phonon coupling and the soft phonon mode in TiSe_2_. *Phys. Rev. Lett.* **107**, 266401 (2011). 22243169 10.1103/PhysRevLett.107.266401

[10] Di Salvo, F. J., Moncton, D. & Waszczak, J. Electronic properties and superlattice formation in the semimetal TiSe_2_. *Phys. Rev. B* **14**, 4321 (1976).

[11] Pillo, T. et al. Photoemission of bands above the Fermi level: The excitonic insulator phase transition in 1T-TiSe_2_. *Phys. Rev. B* **61**, 16213 (2000).

[12] Cercellier, H. et al. Evidence for an excitonic insulator phase in 1 T-TiSe_2_. *Phys. Rev. Lett.* **99**, 146403 (2007). 17930692 10.1103/PhysRevLett.99.146403

[13] Monney, C. et al. Temperature-dependent photoemission on 1 T -TiSe_2_: Interpretation within the exciton condensate phase model. *Phys. Rev. B* **81**, 155104 (2010).

[14] Rohwer, T. et al. Collapse of long-range charge order tracked by time-resolved photoemission at high momenta. *Nature* **471**, 490–493 (2011). 21389987 10.1038/nature09829

[15] Rossnagel, K. On the origin of charge-density waves in select layered transition-metal dichalcogenides. *J. Phys.: Condens. Matter* **23**, 213001 (2011). 21558606 10.1088/0953-8984/23/21/213001

[16] van Wezel, J., Nahai-Williamson, P. & Saxena, S. S. Exciton-phonon-driven charge density wave in tise_2_. *Phys. Rev. B—Condens. Matter Mater. Phys.* **81**, 165109 (2010).

[17] Porer, M. et al. Non-thermal separation of electronic and structural orders in a persisting charge density wave. *Nat. Mater.* **13**, 857–861 (2014). 25038729 10.1038/nmat4042

[18] Kurtz, F. et al. Non-thermal phonon dynamics and a quenched exciton condensate probed by surface-sensitive electron diffraction. *Nat. Mater.* **23**, 890–897 (2024). 38688990 10.1038/s41563-024-01880-6 PMC11230895

[19] Novko, D., Torbatian, Z. & Lončarić, I. Electron correlations rule the phonon-driven instability in single-layer TiSe_2_. *Phys. Rev. B* **106**, 245108 (2022).

[20] Kaneko, T., Ohta, Y. & Yunoki, S. Exciton-phonon cooperative mechanism of the triple- q charge-density-wave and antiferroelectric electron polarization in TiSe_2_. *Phys. Rev. B* **97**, 155131 (2018).

[21] Otto, M. R. et al. Mechanisms of electron-phonon coupling unraveled in momentum and time: The case of soft phonons in TiSe_2_. *Sci. Adv.* **7**, eabf2810 (2021). 33980488 10.1126/sciadv.abf2810 PMC8115930

[22] Michael, S. & Schneider, H. C. Optical amplification in a charge density wave phase of a quasi-two-dimensional material. *Phys. Rev. B* **105**, 235108 (2022).

[23] Monney, C. et al. Revealing the role of electrons and phonons in the ultrafast recovery of charge density wave correlations in 1 T - TiSe_2_. *Phys. Rev. B* **94**, 165165 (2016).

[24] Hedayat, H. et al. Excitonic and lattice contributions to the charge density wave in 1T-TiSe_2_ revealed by a phonon bottleneck. *Phys. Rev. Res.* **1**, 023029 (2019).

[25] Duan, S. et al. Optical manipulation of electronic dimensionality in a quantum material. *Nature* **595**, 239–244 (2021). 34234338 10.1038/s41586-021-03643-8

[26] Hellmann, S. et al. Time-domain classification of charge-density-wave insulators. *Nat. Commun.* **3**, 1069 (2012). 22990865 10.1038/ncomms2078

[27] Kogar, A. et al. Signatures of exciton condensation in a transition metal dichalcogenide. *Science* **358**, 1314–1317 (2017). 29217574 10.1126/science.aam6432

[28] Murakami, Y. et al. Collective modes in excitonic insulators: Effects of electron-phonon coupling and signatures in the optical response. *Phys. Rev. B* **101**, 195118 (2020).

[29] Sugai, S., Takayanagi, Y. & Hayamizu, N. Phason and amplitudon in the charge-density-wave phase of one-dimensional charge stripes in La_2−*x*_Sr_*x*_CuO_4_. *Phys. Rev. Lett.* **96**, 137003 (2006). 16712024 10.1103/PhysRevLett.96.137003

[30] Baldini, E. et al. The spontaneous symmetry breaking in Ta_2_NiSe_5_ is structural in nature. *Proc. Natl. Acad. Sci.* **120**, e2221688120 (2023). 37071679 10.1073/pnas.2221688120 PMC10151608

[31] Ning, H. et al. Signatures of ultrafast reversal of excitonic order in Ta_2_NiSe_5_. *Phys. Rev. Lett.* **125**, 267602 (2020). 33449742 10.1103/PhysRevLett.125.267602

[32] Mazza, G. et al. Nature of symmetry breaking at the excitonic insulator transition: Ta_2_NiSe_5_. *Phys. Rev. Lett.* **124**, 197601 (2020). 32469559 10.1103/PhysRevLett.124.197601

[33] Zenker, B., Fehske, H. & Beck, H. Fate of the excitonic insulator in the presence of phonons. *Phys. Rev. B* **90**, 195118 (2014).

[34] Singh, B. et al. Ferroaxial density wave from intertwined charge and orbital order in rare-earth tritellurides. *Nat. Phys.* **21**, 1578–1586 (2025).

[35] Wang, Y. et al. Axial Higgs mode detected by quantum pathway interference in RTe_3_. *Nature* **606**, 896–901 (2022). 35676485 10.1038/s41586-022-04746-6

[36] Sheng, S. et al. Terahertz spectroscopy of collective charge density wave dynamics at the atomic scale. *Nat. Phys.* **20**, 1603–1608 (2024). 39416850 10.1038/s41567-024-02552-7 PMC11473363

[37] Kim, S. et al. Observation of a massive phason in a charge-density-wave insulator. *Nat. Mater.* **22**, 429–433 (2023). 36894771 10.1038/s41563-023-01504-5

[38] Bae, S. et al. Dynamic competition between phason and amplitudon observed by ultrafast multimodal scanning tunneling microscopy. *arXiv: 2507.11627* (2025).

[39] Burian, M. et al. Structural involvement in the melting of the charge density wave in 1 T-TiSe_2_. *Phys. Rev. Res.* **3**, 013128 (2021).

[40] Cheng, Y. et al. Light-induced dimension crossover dictated by excitonic correlations. *Nat. Commun.* **13**, 963 (2022). 35181649 10.1038/s41467-022-28309-5 PMC8857203

[41] Ou, Y. et al. Incoherence-to-coherence crossover observed in charge-density-wave material 1T-TiSe_2_. *Nat. Commun.* **15**, 9202 (2024). 39448597 10.1038/s41467-024-53647-x PMC11502827

[42] Lian, C., Zhang, S.-J., Hu, S.-Q., Guan, M.-X. & Meng, S. Ultrafast charge ordering by self-amplified exciton–phonon dynamics in TiSe_2_. *Nat. Commun.* **11**, 43 (2020). 31896745 10.1038/s41467-019-13672-7 PMC6940384

[43] Cheng, Y. et al. Ultrafast formation of topological defects in a two-dimensional charge density wave. *Nat. Phys.* **20**, 54–60 (2024).

[44] Yang, J.-A., Pellatz, N., Wolf, T., Nandkishore, R. & Reznik, D. Ultrafast magnetic dynamics in insulating YBa_2_Cu_3_O_6+x_ revealed by time resolved two-magnon Raman scattering. *Nat. Commun.* **11**, 2548 (2020). 32439836 10.1038/s41467-020-16275-9 PMC7242324

[45] Chou, T.-H. et al. Ultrafast Raman thermometry in driven YBa_2_Cu_3_O_6.48_. *Phys. Rev. B* **109**, 195141 (2024).

[46] Glier, T. E. et al. Direct observation of the Higgs particle in a superconductor by non-equilibrium Raman scattering (2024).

[47] Duong, D. L. et al. Raman characterization of the charge density wave phase of 1T-TiSe_2_: from bulk to atomically thin layers. *ACS Nano* **11**, 1034–1040 (2017). 28045499 10.1021/acsnano.6b07737

[48] Holy, J. A., Woo, K. C., Klein, M. V. & Brown, F. C. Raman and infrared studies of superlattice formation in TiSe_2_. *Phys. Rev. B* **16**, 3628–3637 (1977).

[49] Volkov, P. A. et al. Critical charge fluctuations and emergent coherence in a strongly correlated excitonic insulator. *npj Quantum Mater.* **6**, 52 (2021).

[50] Kim, M.-J. et al. Phononic soft mode behavior and a strong electronic background across the structural phase transition in the excitonic insulator Ta_2_NiSe_5_. *Phys. Rev. Res.* **2**, 042039 (2020).

[51] Kim, K. et al. Direct observation of excitonic instability in Ta_2_NiSe_5_. *Nat. Commun.* **12**, 1969 (2021). 33785740 10.1038/s41467-021-22133-z PMC8010035

[52] Bianco, R., Calandra, M. & Mauri, F. Electronic and vibrational properties of TiSe_2_ in the charge-density-wave phase from first principles. *Phys. Rev. B* **92**, 094107 (2015).

[53] Karam, T. E., Hu, J. & Blake, G. A. Strongly coupled electron–phonon dynamics in few-layer TiSe_2_ exfoliates. *ACS Photonics* **5**, 1228–1234 (2018).

[54] Thomson, M. D. et al. Phase-channel dynamics reveal the role of impurities and screening in a quasi-one-dimensional charge-density wave system. Sci. Rep. **7**, 2039 (2017). 28515446 10.1038/s41598-017-02198-x PMC5435704

[55] Kim, K. et al. Origin of the chiral charge density wave in transition-metal dichalcogenide. *Nat. Phys.* **20**, 1919–1926 (2024).

[56] Huber, M. et al. Revealing the order parameter dynamics of 1T-TiSe_2_ following optical excitation. *Sci. Rep.* **12**, 15860 (2022). 36151110 10.1038/s41598-022-19319-w PMC9508156

[57] Huber, M. et al. Ultrafast creation of a light-induced semimetallic state in strongly excited 1T-TiSe_2_. *Sci. Adv.* **10**, eadl4481 (2024). 38728393 10.1126/sciadv.adl4481 PMC11086600

[58] Hedayat, H. et al. Investigation of the non-equilibrium state of strongly correlated materials by complementary ultrafast spectroscopy techniques. *N. J. Phys.* **23**, 033025 (2021).

[59] Chen, P. et al. Charge density wave transition in single-layer titanium diselenide. *Nat. Commun.* **6**, 8943 (2015). 26568512 10.1038/ncomms9943 PMC4660365

[60] Möhr-Vorobeva, E. et al. Nonthermal Melting of a Charge Density Wave in TiSe_2_. *Phys. Rev. Lett.* **107**, 036403 (2011). 21838383 10.1103/PhysRevLett.107.036403

[61] Versteeg, R. et al. A tunable time-resolved spontaneous Raman spectroscopy setup for probing ultrafast collective excitation and quasiparticle dynamics in quantum materials. *Struct. Dyn.* **5**, 044301 (2018). 10.1063/1.5037784 PMC6051769 30057929

[62] Murakami, Y., Golež, D., Eckstein, M. & Werner, P. Photoinduced nonequilibrium states in Mott insulators. *Rev. Mod. Phys.* **97**, 035001 (2025).

[63] Holt, M., Zschack, P., Hong, H., Chou, M. Y. & Chiang, T.-C. X-ray studies of phonon softening in tise_2_. *Phys. Rev. Lett.* **86**, 3799–3802 (2001). 11329327 10.1103/PhysRevLett.86.3799

[64] Kresse, G. & Joubert, D. From ultrasoft pseudopotentials to the projector augmented-wave method. *Phys. Rev. B* **59**, 1758–1775 (1999).

[65] Hafner, J. Ab-initio simulations of materials using VASP: Density-functional theory and beyond. *J. Comput. Chem.* **29**, 2044–2078 (2008). 18623101 10.1002/jcc.21057

[66] Grimme, S. Accurate description of van der Waals complexes by density functional theory including empirical corrections. *J. Comput. Chem.* **25**, 1463–1473 (2004). 15224390 10.1002/jcc.20078

[67] Togo, A. & Tanaka, I. First principles phonon calculations in materials science. *Scr. Materialia* **108**, 1–5 (2015).

[68] Monkhorst, H. J. & Pack, J. D. Special points for Brillouin-zone integrations. *Phys. Rev. B* **13**, 5188–5192 (1976).

