# Supplementary material for: Tracking the catastrophic collapse of hybrid exciton-phonon order in a quantum material
Source: Nat Commun. 2026 Jul 30;17:7607. doi: 10.1038/s41467-026-75887-9 (PMC13424097; doi:10.1038/s41467-026-75887-9)
Supplement: Supplementary file 1 — Supplementary Information [file 41467_2026_75887_MOESM1_ESM.pdf]

Supplementary Information for

Tracking the Catastrophic Collapse of Hybrid Exciton-Phonon Order in a Quantum Material

Omar Abdul-Aziz[1], Danilo Comini[1], Johannes Lang[2], Nils Bartel[2], Michael Buchhold[3], Sebastian Diehl[2], Daniel Wolverson[4], Charles J. Sayers[5], Giulio Cerullo[5,6], Paul H. M. van Loosdrecht[1], and Hamoon Hedayat[1]

[1]II. Physikalisches Institut, Universität zu Köln, Zülpicher Straße 77, Köln D-50937, Germany

[2]Institut für Theoretische Physik, Universität zu Köln, Zülpicher Straße 77, D-50937 Köln, Germany

[3]Institut für Theoretische Physik, Universität Innsbruck, Technikerstraße 21a, Innsbruck, 6020,Austria

[4]Department of Physics and Centre for Photonics and Photonic Materials, University of Bath, BA2 7AY Bath, UK

[5]Dipartimento di Fisica, Politecnico di Milano, Piazza L. da Vinci 32, 20133 Milan, Italy

[6]Istituto di Fotonica e Nanotecnologie, Consiglio Nazionale delle Ricerche, Piazza L. da Vinci 32, 20133 Milano, Italy

Supplementary Figures S1–S4 present additional data corresponding to Figs. 1–4 of the main text, respectively.

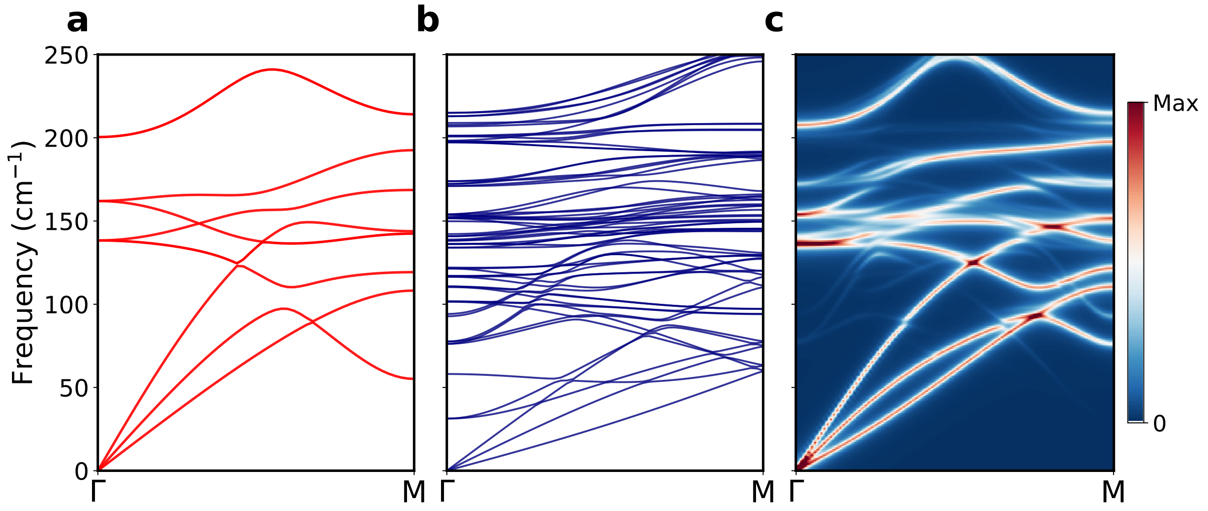

Figure S1: **Phonon dispersion of 1*T*-TiSe$_2$.** (a) Phonon dispersion in the normal phase (space group $P\bar{3}m1$) from DFT simulations. (b) Phonon dispersion in the CDW phase (space group $P\bar{3}c1$). The A_{1g}^* and E_g^* modes originate from M (or L)-point phonons that fold to the new Γ' point in the reduced Brillouin zone, consistent with the zone-folding picture of the CDW. (c) Unfolded phonon dispersion of the CDW phase of TiSe$_2$. The false-colour scale (arbitrary units), indicated by the colour bar on the right, shows the weights of the projections of the CDW phonon branches onto the irreducible representations of the little groups of the 3-atom primitive structure. The k-point labels refer to the unfolded (primitive) Brillouin zone, from Γ to M. The phonon unfolding was performed using the UPHO code [1].

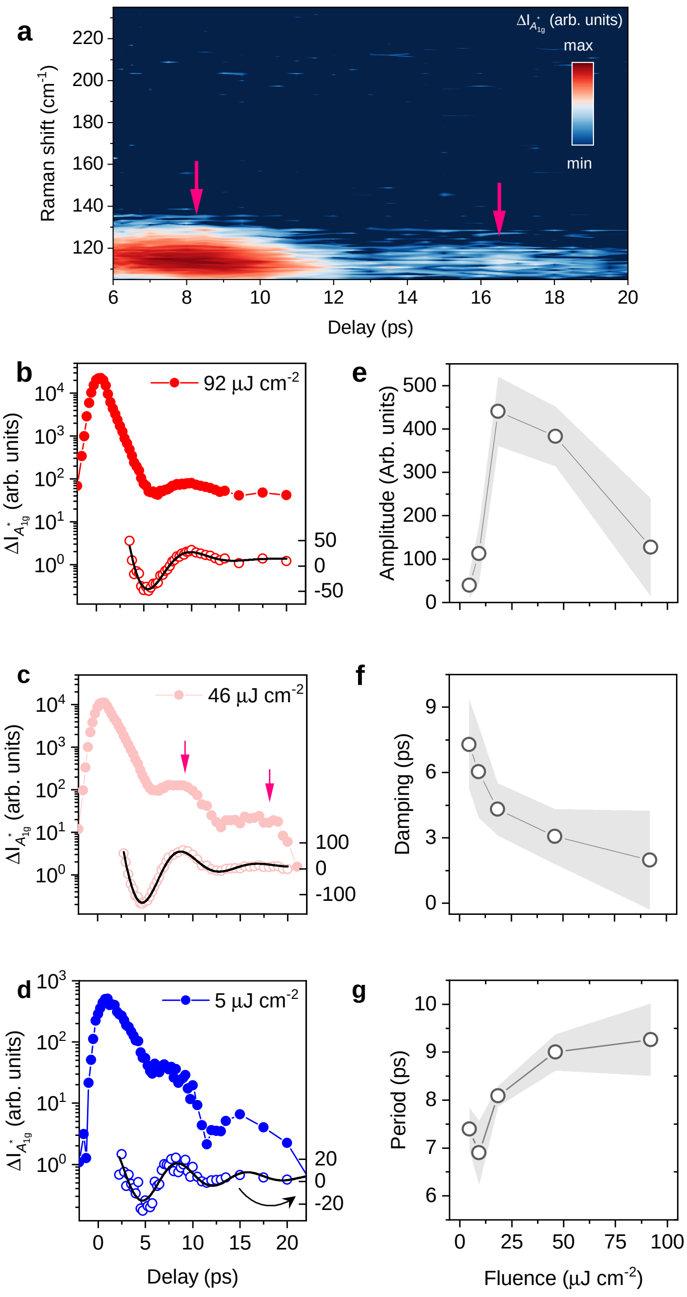

Figure S2: **Fluence-dependent behavior of the hybrid exciton-phonon mode. a,** Time-resolved Raman map of the photoinduced A_{1g}^* response at low temperature over an extended delay range, corresponding to the dataset shown in (Fig. 2b). The modulation of the folded CDW phonon intensity is visible at long delays (arrows). **b-d,** Residual coherent oscillations of the photoinduced A_{1g}^* phonon dynamics after exponential background subtraction (Supplementary Note 6) for three representative fluences. Black lines show damped-cosine fits to the residual oscillatory component. Extracted amplitude (**e**), damping time (**f**), and period (**g**) as a function of excitation fluence. Grey shaded regions indicate the fitting uncertainties of the extracted parameters. At low fluence, the oscillations are long-lived and periodic; with increasing fluence, the hybrid mode softens and damps, disappearing above 100 μJ cm^{-2}. This behavior indicates a fluence-driven collapse of the hybrid locked phason mode.

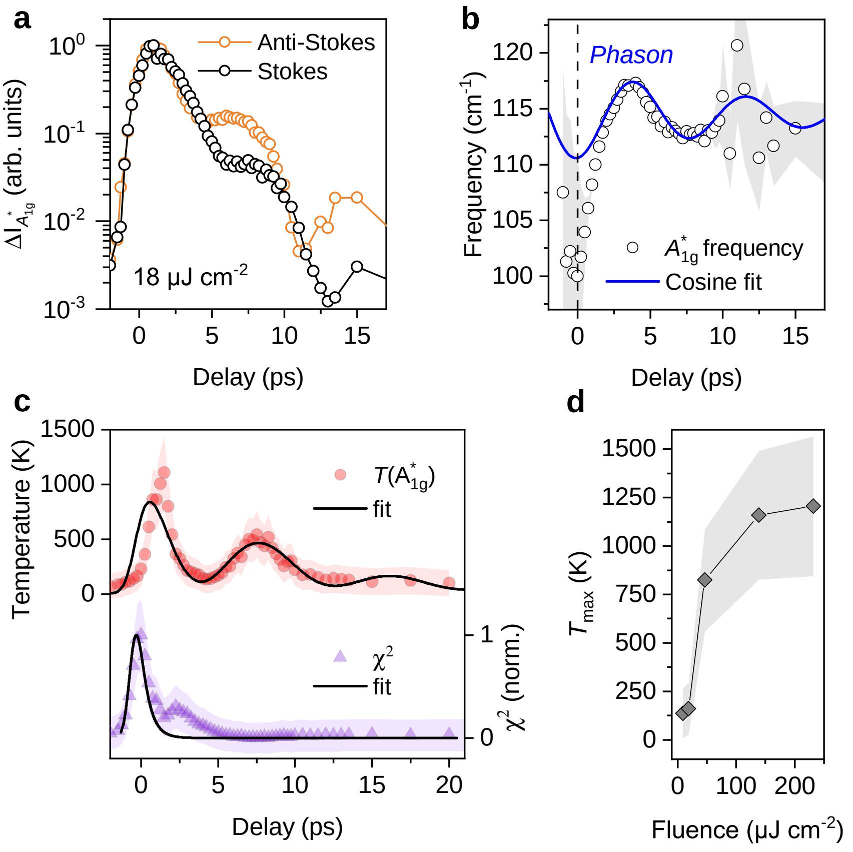

Figure S3: **Comparison of Stokes and anti-Stokes dynamics, and extracted phonon temperature. a,** Stokes and anti-Stokes dynamics of A^*_{1g} at $F = 18\ \mu\text{J}\,\text{cm}^{-2}$. The anti-Stokes signal closely tracks the coherent oscillations seen in the Stokes channel with enhanced relative amplitude, demonstrating the coupling of the locked phason to the CDW phonon. **b,** Time-dependent frequency of the A^*_{1g} mode $\Omega_{A^*_{1g}}(t)$. The mode exhibits a pronounced initial softening, followed by a cosine oscillatory response of the same period as the locked phason. In all panels, the shaded regions indicate the uncertainties from the fitting procedure. **c,** Extracted phonon temperature $T_{\text{ph}}(t)$ and time-dependent Raman susceptibility $\chi(t)$ at $F = 46\ \mu\text{J}\,\text{cm}^{-2}$. The data reveal a monotonically decaying $\chi(t)$, indicative of a loss of CDW-related susceptibility, together with oscillatory dynamics of $T_{\text{ph}}(t)$ that follow the low-frequency modulation of the A^*_{1g} mode. **d,** Maximum phonon temperature $T^{\text{max}}_{\text{ph}}$ as a function of fluence, extracted from fits to the anti-Stokes dynamics. The apparent divergence and subsequent saturation of $T^{\text{max}}_{\text{ph}}$ above the critical fluence F_c indicate that the CDW phonon enters an overheated regime.

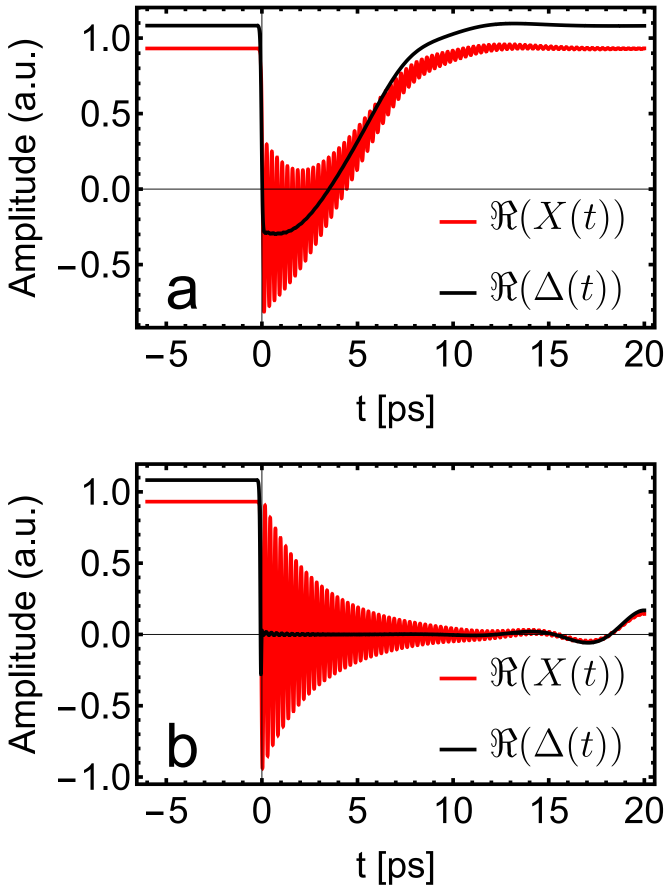

Figure S4: **Theoretical simulation of the exciton-phonon order.** Simulated time evolution of the real parts of the coupled order parameters following a sudden quench at time $t = 0$. For low fluence ($F < F_c$) (**a**), the initial state is recovered quickly, following a phase of violent oscillations of the phononic order. For high fluence ($F > F_c$) (**b**), the excitonic order is lost, and the subsequent relaxation occurs on a much longer time scale. Lattice displacement is shown by $\Re(X(t))$ (red) and the excitonic order parameter by $\Re(\Delta(t))$ (black).

Note 1 Sample growth and Characterization

High-quality $1T$-$TiSe_2$ single crystals were grown using the chemical vapor transport method. Titanium (99.9%) and selenium ($> 99.9\%$) powders were sealed inside an evacuated quartz ampoule, together with iodine ($> 99.9\%$) which acts as the transport agent. To ensure the correct stoichiometry, a slight selenium excess was included. Single crystals with a typical size of $4 \times 4 \times 0.1\,\mathrm{mm}^3$ were selected for TR-Raman measurements.

To verify sample quality and confirm the CDW phase transition, standard four-probe transport measurements were performed on the $1T$-$TiSe_2$ single crystals. The transition temperature is determined as $T_{\mathrm{CDW}} = 202$ K from the derivative of the resistivity, consistent with near-stoichiometric crystals [2].

Note 2 Symmetry Analysis of Raman Modes in $1T$-$TiSe_2$ and DFT Calculated Unfolded Dispersion

2.1 Symmetry Analysis of Raman Modes

The symmetry classification of Raman-active phonon modes in $1T$-$TiSe_2$ is fundamental to interpreting its vibrational spectra and understanding the structural dynamics associated with its CDW phase transition [3]. In the high-temperature normal phase ($T > 202\,\mathrm{K}$), $1T$-$TiSe_2$ crystallizes in a trigonal structure belonging to the space group D^3_{3d} ($P\bar{3}m1$), with one formula unit per primitive cell. Group theoretical analysis of the nine zone-center vibrational modes yields the decomposition into irreducible representations:

$$\Gamma_{\mathrm{normal}} = A_{1g} + E_g + 2A_{2u} + 2E_u. \tag{1}$$

Among these, the $A_{1\mathrm{g}}$ and E_g modes are Raman-active. The $A_{1\mathrm{g}}$ mode corresponds to symmetric, out-of-plane vibrations of the Se atoms, while the doubly degenerate E_g mode involves symmetric, in-plane shear-like vibrations of the Se atoms relative to the Ti atoms. The A_{2u} and E_u representations correspond to infrared-active and acoustic modes, respectively, and are therefore silent in Raman spectroscopy.

Below the CDW transition temperature $T_{\mathrm{CDW}} \approx 202\,\mathrm{K}$, the crystal undergoes a structural phase transition, forming a $2a_0 \times 2a_0 \times 2c_0$ superlattice described by the space group D^4_{3d} ($P\bar{3}c1$). This reconstruction involves the folding of Brillouin zone edge points, particularly the L-points where the CDW instability occurs, to the new zone center (Γ'). The new, larger unit cell contains eight formula units (24 atoms), leading to a significantly richer phonon spectrum. The 72 vibrational modes at the zone center of the CDW phase decompose as:

$$\Gamma_{\mathrm{CDW,\ optical}} = 4A_{1g} + 4A_{2g} + 4E_g + 3A_{1u} + 3A_{2u} + 7E_u. \tag{2}$$

In this broken-symmetry phase, the number of Raman-active modes increases to four $A_{1\mathrm{g}}$ and four E_g modes. Prominent new features emerge in the Raman spectrum, most notably the CDW amplitude mode ($A^*_{1\mathrm{g}}$) at approximately $116\,\mathrm{cm}^{-1}$ and an interlayer shear mode (E^*_g) near $74\,\mathrm{cm}^{-1}$. These modes are direct spectroscopic signatures of the reduced lattice symmetry. The $A^*_{1\mathrm{g}}$ amplitude mode involves out-of-phase displacement of the Ti and Se atoms, which

directly modulates the CDW gap and the magnitude of the periodic lattice distortion (PLD). Its observation and behavior are thus central to understanding the lattice component of the CDW [4].

To further verify that the mode assignment used throughout the main text is consistent with the expected Raman selection rules, we performed polarization-resolved static Raman measurements at 100 K in parallel (XX) and crossed (XY) geometries, as shown in Fig. S5. The spectra follow the standard symmetry expectations for $1T$-$TiSe_2$ in the CDW phase [3]: the fully symmetric A_{1g} mode near 200 cm^{-1} is strongly enhanced in XX and strongly suppressed in XY, while the folded A^*_{1g} mode also appears predominantly in XX. By contrast, the E_g and folded E^*_g modes remain visible in both polarization configurations, consistent with their non-fully-symmetric character. This polarization dependence confirms that the folded A^*_{1g} feature tracked in the time-resolved measurements is observed in the expected symmetry channel, and it also motivates our choice of XX geometry in TR-Raman, where maximizing sensitivity to the folded A^*_{1g} response is essential for resolving its non-equilibrium dynamics and low-frequency modulation.

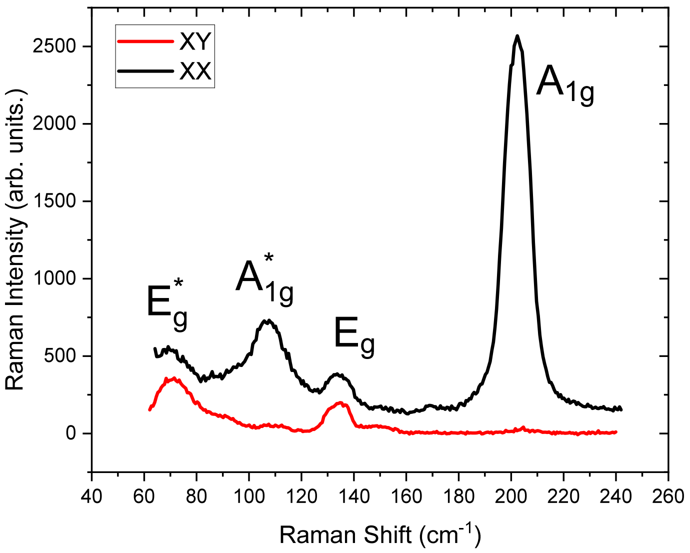

Figure S5: **Polarization-resolved static Raman spectra of $1T$-$TiSe_2$ at 100 K.** Raman spectra measured in parallel (XX, black) and crossed (XY, red) polarization geometries. The fully symmetric A_{1g} mode is dominant in XX and strongly suppressed in XY, while the folded A^*_{1g} mode is also strongest in XX. The E_g and folded E^*_g modes remain visible in both XX and XY. The observed polarization dependence is consistent with the expected D_{3d} Raman selection rules and supports the use of XX geometry in the time-resolved Raman measurements to maximize sensitivity to the folded A^*_{1g} mode.

2.2 Discussion of unfolded dispersion

The band-unfolding method [5] decomposes the phonon eigenvectors of a supercell structure (which is often a supercell representing a disordered random alloy, but is here the CDW phase)

to obtain an effective phonon dispersion displayed as a function of wavevector in the underlying primitive lattice (here, the non-CDW phase). The results are instructive in understanding the influence of the CDW on the phonon band structure, as shown in a study of the CDW phase of $NbSe_2$ [6]. The UPHO package [7] was used in conjunction with Phonopy [8, 9] to produce the unfolded CDW phonon dispersion shown in Supplementary Fig. S1. The folded phonon dispersion and the computational methods used to obtain it are discussed in the Supporting Information.

2.3 DFT Computational methods

The plane-wave density functional theory (DFT) code VASP [10] was used to calculate the phonon dispersion using the finite displacement method as implemented in the PHONOPY package [8, 9] with $2 \times 2 \times 2$ supercells (i.e, 192 atoms, given a CDW cell of 24 atoms). Atomic coordinates for the CDW phase were taken from the detailed study of CDW $1T$-$TiSe_2$ lattice dynamics by Bianco *et al.* [11] and were relaxed with the constraint that Ti positions retained the CDW displacements; inter-layer van der Waals interactions were modelled at the Grimme-D2 level [12]. With these choices, the calculated CDW phonon dispersion was purely real for electronic smearing above $kT \sim$100 K. Raman-active modes were identified by analysis of the mode symmetries at Γ which, for example, identifies the A^*_{1g} mode at a calculated Raman shift of 117 cm^{-1}.

Note 3 Experimental Methods: Time-Resolved Raman and Optical Spectroscopy

Our time-resolved spontaneous Raman spectroscopy (TR-Raman) setup enables ultrafast measurements of vibrational and electronic dynamics in quantum materials with high temporal and spectral resolution. A schematic overview is shown in Fig. S6.

The laser source is a Yb:KGW-based chirped-pulse amplifier (PHAROS, Light Conversion) operating at 1024 nm, 35 kHz, and up to 18 W output power. It simultaneously drives two optical parametric amplifier (OPA) branches: a femtosecond OPA (fs-OPA, ORPHEUS) that delivers broadband tunable pump pulses (350-2000 nm, $\sim$250 fs), and a picosecond OPA (ps-OPA) line incorporating a second-harmonic bandwidth compressor (SHBC) that generates narrowband probe pulses ($\sim$1.5 ps, $\Delta\nu \sim$ 10-15 cm^{-1}) centered around 512 nm. The fs-OPA ensures impulsive, selective excitation, while the ps-OPA provides the spectral resolution needed to resolve low-energy Raman-active phonons with minimal Fourier broadening.

In the optical layout, the pump and probe beams are aligned collinearly and focused onto the sample inside a closed-cycle helium cryostat (4–400 K) using a long-working-distance objective in a backscattering configuration. The temporal delay between pump and probe is controlled by a high-precision linear motorized delay stage. The backscattered light is collected either through a confocal or macro-Raman configuration and analyzed by a triple-subtractive spectrometer (TriVista 555, S&I GmbH). The first two stages are operated in subtractive mode, forming a tunable ultranarrow notch filter that suppresses stray light by more than 10^8, essential for detecting weak low-frequency Raman signals. The third stage disperses the filtered signal onto

a back-illuminated, liquid-nitrogen-cooled CCD, allowing parallel detection of Stokes and anti-Stokes spectra [13].

To complement the Raman measurements, a time-resolved reflectivity channel is used. After interacting with the sample, a portion of the probe beam is diverted to a fast silicon photodiode. The pump beam is modulated using an optical chopper synchronized to a lock-in amplifier. This arrangement allows measurement of differential reflectivity $\Delta R/R$ with high sensitivity, providing a direct probe of electronic dynamics such as carrier relaxation and excitonic suppression. Importantly, this dual-modality detection scheme enables simultaneous tracking of both vibrational and electronic order parameters under identical excitation conditions [14].

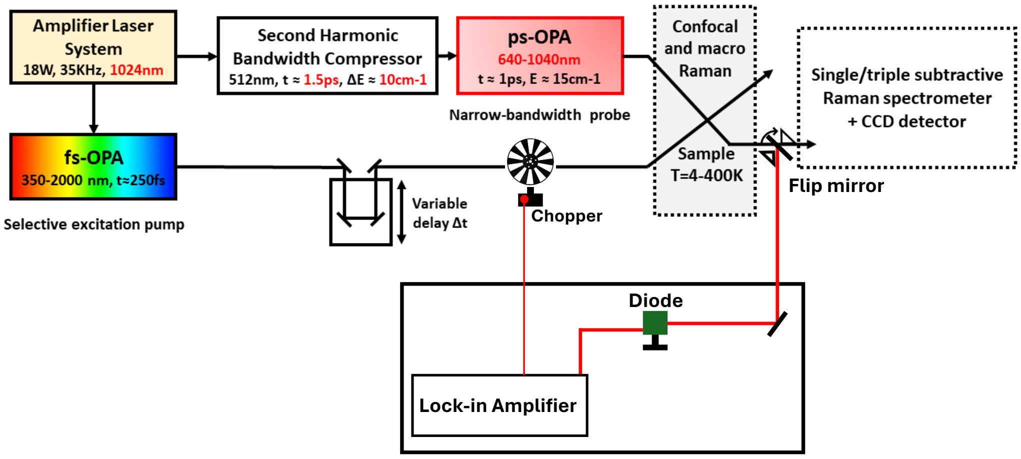

Figure S6: **Time-resolved Raman and reflectivity setup.** A high-repetition-rate Yb:KGW amplifier drives both a femtosecond OPA (fs-OPA) for broadband pump pulses and a picosecond OPA (via second-harmonic bandwidth compression) for narrowband probe pulses. Pump and probe are focused collinearly onto the sample (4–295 K). Scattered light is analyzed using a triple-subtractive spectrometer with a CCD detector. In TRR spectroscopy, a chopper-modulated pump beam and photodiode-reflected probe enable detection via a lock-in amplifier.

Note 4 Data Processing and Analysis: Time-Resolved Raman Spectra

The analysis of the TR-Raman spectra is a multi-step process designed to extract the dynamics of individual phonon modes and the underlying electronic continuum.

4.1 Data Pre-processing and Background Subtraction

Raw spectra acquired from the CCD are first processed to remove spurious signals from cosmic rays. A crucial subsequent step is the subtraction of the broad, non-vibrational background arising from inelastic light scattering by the transient population of photoexcited carriers. At

negative time delays, this electronic continuum exhibits a clear exponential lineshape. This shape is used as a model for the background at all time delays, with its amplitude allowed to vary as a fitting parameter to account for the dynamics of the photoexcited carrier population.

4.2 Spectral Fitting Model

To quantify the dynamics of phonon modes in $1T$-$TiSe_2$, we employ a systematic spectral fitting approach applied to time-resolved Raman spectra under both equilibrium and photoexcited conditions. Figure S7 displays typical Raman spectra at room temperature (295 K) and in the CDW phase (5 K) without pump excitation. As expected, the 295 K spectrum shows only the A_{1g} phonon near $200\,\mathrm{cm}^{-1}$; the E_g mode is symmetry-allowed but too weak to be observed clearly in our parallel polarization geometry. In contrast, at 5 K, the CDW-induced A^*_{1g} mode emerges at $\sim 120\,\mathrm{cm}^{-1}$, along with a pronounced low-frequency background.

Upon pump excitation, a broad, fluence-dependent continuum appears early positive delays. This component, most evident below $150\,\mathrm{cm}^{-1}$, arises from photoinduced carrier excitation and its associated optical response. The intensity and shape of this feature evolve systematically with pump fluence and delay. Figure S8 shows the logarithmic intensity plot of this background for various fluences. Its linear decay in logarithmic scale justifies modeling it with an exponential function, which we refer to as the *Low-Energy Raman Continuum* (LERC). Subtraction of this term effectively isolates the phonon response.

After LERC subtraction, the remaining vibrational spectrum is fitted using a sum of Gaussian functions. This representation accurately captures the convolution of the intrinsic Lorentzian phonon lineshape with the Gaussian spectral response of the probe ($\Delta\nu \approx 10$ cm^{-1}). The complete model is expressed as:

$$I(x,t) = A(t) + \sum_{i=1}^{3} \mathrm{amp}_i(t) \cdot \exp\left(-\frac{(x - \mathrm{cen}_i(t))^2}{2 \cdot \mathrm{sigma}_i(t)^2}\right), \tag{3}$$

where x is the Raman shift, and for each mode i (e.g., A^*_{1g}, E^*_g, A_{1g}), the amplitude $\mathrm{amp}_i(t)$, center frequency $\mathrm{cen}_i(t)$, and linewidth $\mathrm{sigma}_i(t)$ are extracted as functions of the pump-probe delay t. A constant offset $A(t)$ accounts for residual background contributions. Gaussian profiles are chosen to represent the convolution of the phonon lineshape with the instrumental response, allowing direct extraction of time-dependent phonon parameters such as integrated intensity, frequency, and linewidth.

This fitting procedure was applied consistently across all delays and temperatures. Figures S9 and S10 show representative examples of the fits at selected pump-probe delays for 295 K and 5 K, respectively. The decomposition separates the phonon peaks (colored Gaussians) from the electronic continuum (gray). The CDW phase (5 K) reveals the transient evolution and recovery of the A^*_{1g} mode, while at 295 K only the A_{1g} phonon persists, modulated by the carrier-induced background. This analysis forms the foundation for extracting mode-specific dynamics such as frequency softening, coherent amplitude evolution, and linewidth broadening under non-equilibrium excitation.

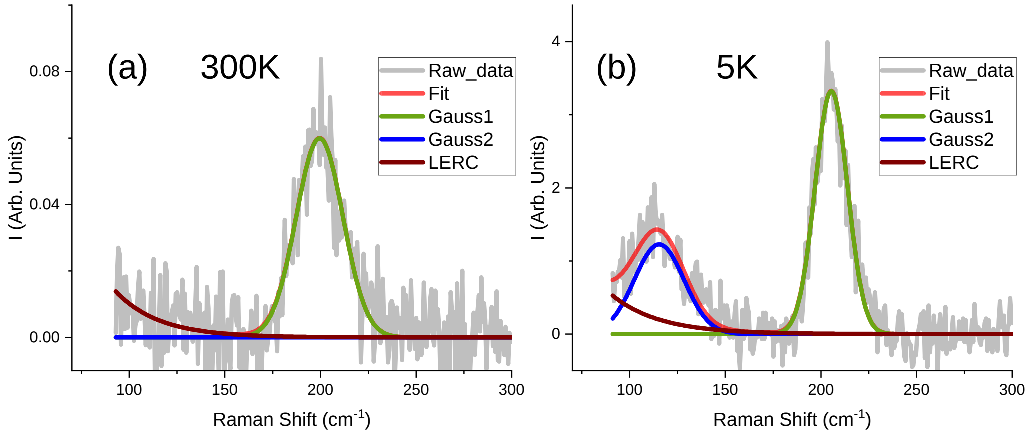

Figure S7: **Static Raman spectra at equilibrium using pulsed laser.** (a) 295 K spectrum showing the A_{1g} phonon at $\sim$200 cm^{-1} and a weak background. (b) 5 K spectrum in the CDW phase displaying both the A^*_{1g} mode at $\sim$117 cm^{-1} and the A_{1g} phonon. Colored curves indicate the individual Gaussian phonon components, the exponential background (LERC), and the overall fit.

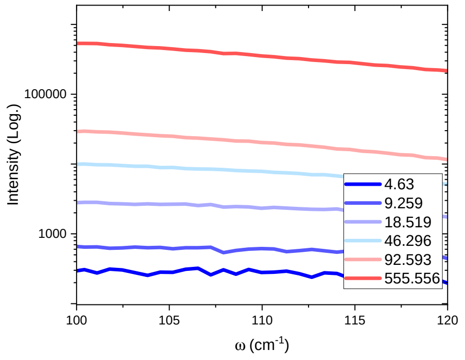

Figure S8: **Logarithmic representation of the photoinduced background.** Raman intensity (log scale) for several pump fluences, showing a linear decay below 120 cm^{-1}. The approximately constant slope across fluences validates the exponential model used for the low-energy Raman continuum (LERC).

Note 5 Determination of Phonon Properties from Raman Stokes Anti-Stokes

Effective Phonon Temperature from Detailed Balance

Time-resolved Stokes (I_S) and anti-Stokes (I_{AS}) Raman intensities provide a direct probe of lattice heating and cooling dynamics. For one-phonon scattering, the respective intensities are given by

$$I_S(\omega_L - \Omega) \propto |\chi_R|^2(\omega_L - \Omega)^4[n(\Omega) + 1], \tag{4}$$

$$I_{AS}(\omega_L + \Omega) \propto |\chi_R|^2(\omega_L + \Omega)^4 n(\Omega), \tag{5}$$

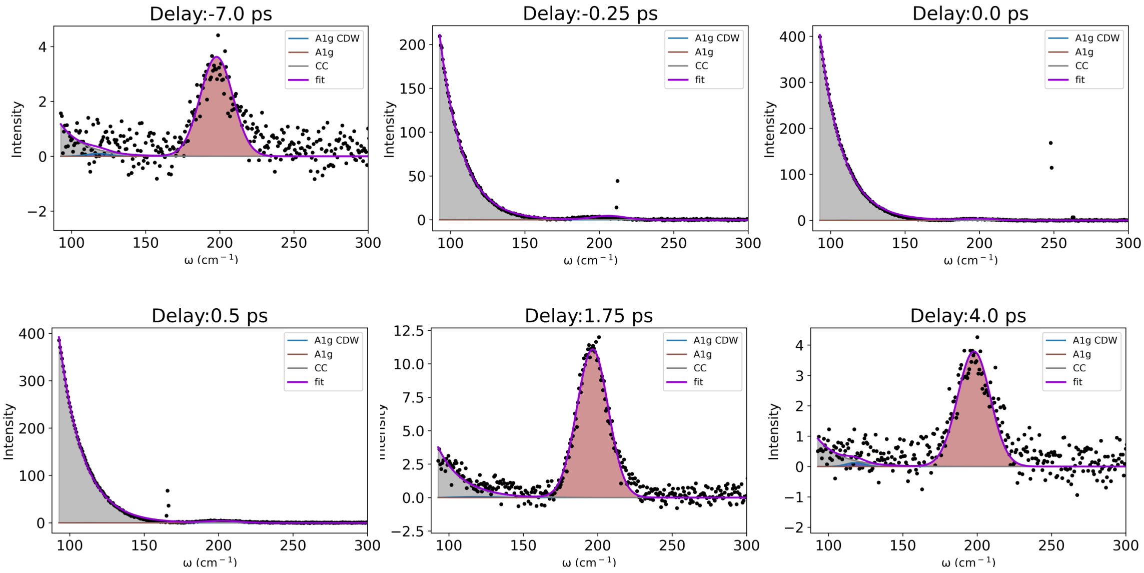

Figure S9: **Transient Raman spectra and fits at 295 K.** Time-resolved spectra and multi-peak fits at selected pump-probe delays from negative to +4 ps. Only the A_{1g} phonon is observed, superimposed on the dynamic low-frequency continuum (gray). The lineshape remains consistent across delays, showing no signatures of the CDW phase.

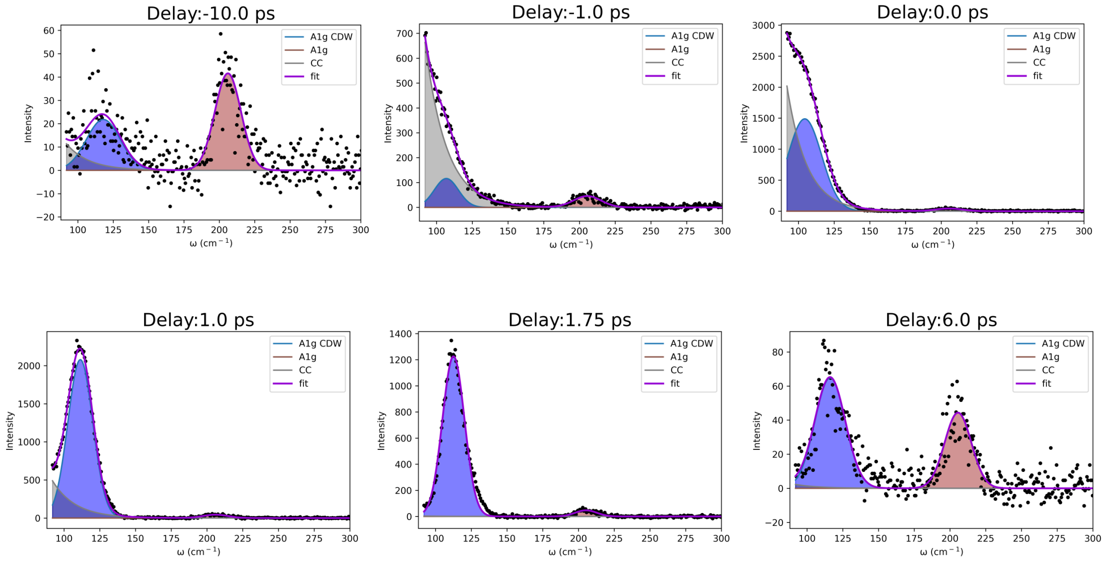

Figure S10: **Transient Raman spectra and fits at 5 K.** Delay-dependent spectra and corresponding multi-peak fits from negative delays to +6 ps in the CDW phase. Both the A_{1g}^{*} and A_{1g} phonons are resolved together with a delay-dependent electronic background. The evolution of the A_{1g}^{*} intensity and its recovery dynamics reflect the transient suppression and reformation of the CDW order.

where ω_L is the probe photon frequency, Ω the phonon frequency, $|\chi_R|^2$ the squared Raman tensor, and $n(\Omega)$ the phonon occupation number. The ratio of anti-Stokes to Stokes intensity follows from the principle of detailed balance:

$$\frac{I_{AS}}{I_S} = \left(\frac{\omega_L + \Omega}{\omega_L - \Omega}\right)^4 \frac{n(\Omega)}{n(\Omega)+1}. \tag{6}$$

This expression remains valid even under non-equilibrium excitation as long as the phonons can be described by a quasi-thermal distribution. From Eq. 6, the transient phonon occupation $n(\Omega, t)$ is extracted at each pump-probe delay, and an effective phonon temperature $T_{ph}(t)$ is obtained by inverting the Bose-Einstein relation:

$$n(\Omega, t) = \frac{1}{\exp\left[\frac{\hbar\Omega}{k_B T_{ph}(t)}\right] - 1} \quad \Rightarrow \quad T_{ph}(t) = \frac{\hbar\Omega}{k_B \ln[1 + 1/n(\Omega, t)]}. \tag{7}$$

Before photoexcitation ($t < 0$), the extracted temperature equals the cryostat base temperature, fixing all spectral prefactors. The resulting time-dependent Stokes, anti-Stokes, and derived $T_{ph}(t)$ traces are shown in Fig. S11. The anti-Stokes signal rises rapidly after excitation, reflecting the transient phonon population, while the Stokes is dominated by the Raman tensor variation. The extracted phonon temperature peaks at ∼7-8 ps and exhibits oscillations at longer delays, indicative of a coherent hybrid mode contribution. In principle, subtle symmetry-related changes in the Raman matrix elements could renormalize the absolute temperature scale extracted from the Stokes/anti-Stokes ratio. We do not expect such effects to alter the central conclusion here for several reasons. A recent study of chiral CDW order in $1T$-$TiSe_2$ suggests that possible chirality-related corrections are small in the present context, since our analysis is performed for the folded A_{1g}^* phonon, which is the primary structural CDW mode rather than the secondary chiral refinement discussed in that work [15]. Then, in our analysis we explicitly separate the phonon-population response from the transient Raman susceptibility: the susceptibility change is confined to the earliest delays, whereas the phonon occupation builds up later and exhibits the delayed behavior discussed in the main text. This temporal separation strongly supports the interpretation that the inferred temperature reflects phonon population dynamics rather than a transient renormalization of the Raman matrix element. Finally, our main conclusion does not rely on the exact absolute value of the extracted temperature, but on its fluence dependence: the folded A_{1g}^* phonon remains clearly visible even when the inferred occupation becomes very large. We therefore use the Stokes/anti-Stokes ratio primarily as an effective measure of phonon occupation, while allowing that small symmetry-related corrections could renormalize the absolute temperature scale.

Probing Electronic Variations via the Raman Tensor

To separate the ultrafast electronic response from the phonon dynamics, we must isolate the Raman tensor, $|\chi_R(t)|^2$, which is sensitive to the transient electronic distribution. The Stokes and anti-Stokes intensities are given by $I_S(t) \propto |\chi_R(t)|^2[n(t) + 1]$ and $I_{AS}(t) \propto |\chi_R(t)|^2 n(t)$, respectively. While the Stokes intensity I_S is often used as a proxy for $|\chi_R|^2$ at equilibrium (where $n \ll 1$), this approximation breaks down in our non-equilibrium experiment, where the pump creates a large phonon population $n(t)$ (see Fig. S11).

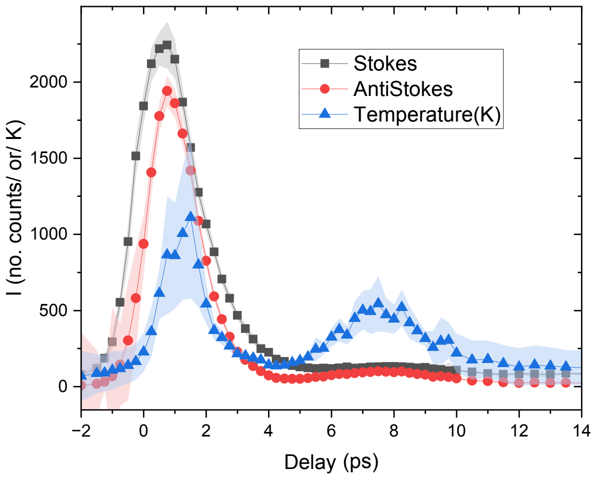

Figure S11: **Transient Stokes and anti-Stokes intensities and derived phonon temperature.** Time-resolved Stokes (black squares) and anti-Stokes (red circles) intensities, together with the extracted effective phonon temperature $T_{\mathrm{ph}}(t)$ (blue triangles). The shaded regions denote the fit uncertainty propagated from the individual Raman-peak fits. The delayed temperature rise and its oscillatory tail indicate the coupling between electronic excitation and lattice modes. Shaded regions represent the uncertainties of the fitted parameters.

Therefore, we first extract the true transient phonon occupation $n(t)$ at each delay using the detailed balance ratio $I_{\mathrm{AS}}(t)/I_{\mathrm{S}}(t)$ (from Eq. 6). With $n(t)$ known, we can then rigorously extract the pure electronic response using the full relation: $|\chi_{\mathrm{R}}(t)|^2 \propto I_{\mathrm{S}}(t)/[n(t)+1]$.

The resulting electronic response, $\chi^2(t)$, is shown in Fig. S12. It exhibits a sub-picosecond rise and decay, in stark contrast to the slower, delayed, and oscillatory dynamics of the phonon temperature $T_{\mathrm{ph}}(t)$ (seen in Fig. S11). The complete lack of a ∼0.13 THz oscillatory modulation in $\chi^2(t)$ provides definitive proof that the long-period oscillations are not an electronic effect (like a quantum beat) but arise from the hybrid phonon-exciton coherence within the lattice.

Temporal Phonon Temperature and Multichannel Dynamical Model

To interpret the extracted phonon temperature $T_{\mathrm{ph}}(t)$ and disentangle the contributions of the electronic, phononic, and hybridized exciton-phonon channels, we employ a phenomenological multi-component model reproducing the observed dynamics:

$$\Delta I(t) = \text{offset} + \left[A_1 e^{-(t-t_0)/\tau_1} + A_2 e^{-(t-t_0)/\tau_2}\right] * G(t)\, H(t) + A_{\mathrm{osc}}\, e^{-(t-t_0)/\tau_{\mathrm{damp}}} \cos\left[\frac{2\pi}{T}(t-t_0)+\phi\right] H(t-t_0), \tag{8}$$

where $G(t)$ represents the Gaussian instrument response (FWHM 1.2 ps) and $H(t)$ is the Heaviside function. The exponential terms describe (i) ultrafast carrier thermalization ($\tau_1 \approx$ 0.5 ps) and (ii) subsequent phonon-lattice relaxation ($\tau_2 \approx 8$ ps), while the oscillatory term captures a damped coherent hybrid mode with period $T \approx 8.6$ ps (0.13 THz) and decay time

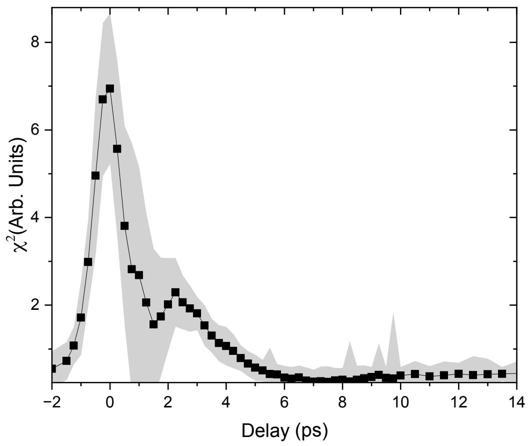

Figure S12: **Transient Raman susceptibility dynamics.** Time-dependent squared Raman tensor $\chi^2(t)$ extracted from Stokes intensity fits. The rapid rise ($<$ 1ps) and decay ($\sim$1.5ps) with a short-lived exponential tail reflect the electronic excitation and dephasing processes. Grey shaded regions represent the uncertainties associated with the fitted parameter. The absence of oscillatory features indicates that the coherent response originates from lattice rather than electronic degrees of freedom.

$\tau_{\text{damp}} \approx 10$ ps. The relative phases of these components reproduce the experimentally observed sequence: an immediate electronic peak, a delayed thermal maximum, and a slowly decaying oscillation.

This model quantitatively reproduces the measured temperature and susceptibility traces, confirming that the ultrafast response of $1T$-$TiSe_2$ is governed by a cascade of processes: rapid electronic excitation, energy transfer to phonons via electron-phonon coupling, and coherent modulation by a hybrid exciton-phonon mode. The hierarchy of timescales and the absence of oscillatory behavior in $\chi^2(t)$ together establish that the low-frequency oscillations are of lattice origin and correspond to the pseudo-Goldstone hybrid mode discussed in the main text.

Note 6 Fluence-Dependent Dynamics of the Hybrid Mode

To quantitatively analyze the hybrid phonon-exciton mode, we examine the residual coherent oscillations of the A_{1g}^* phonon following photoexcitation at different fluences. This procedure is carried out in three steps:

Step 1: Extraction of A_{1g}^* Dynamics

We first isolate the dynamics of the A_{1g}^* phonon by tracking its transient intensity from the time-resolved Raman spectra. The peak is fitted at each pump-probe delay using the spectral decomposition model detailed in Note 5. At low fluence (e.g., 4.6 μJ cm^{-2}), the A_{1g}^* response exhibits a clear oscillatory modulation atop a decaying background.

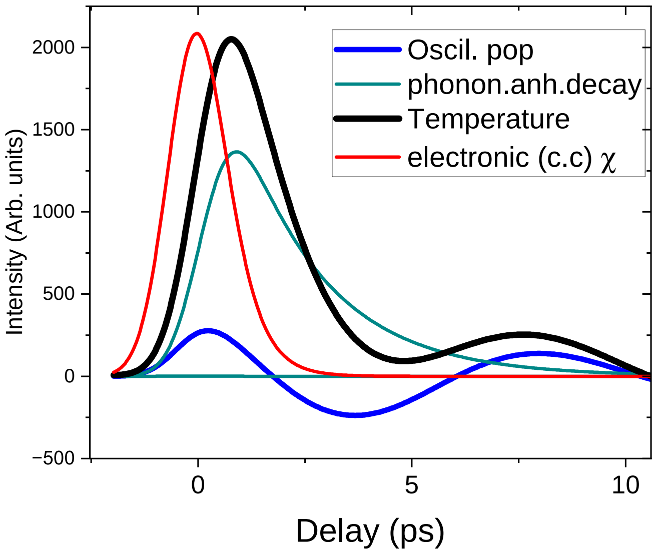

Figure S13: **Simulated decomposition of electronic, phonon, and hybrid contributions.** Modeled temporal components of the transient Raman response: electronic Raman susceptibility $\chi(t)$ (red), anharmonic phonon decay (cyan), effective lattice temperature (black), and coherent hybrid mode population (blue). The simulation reproduces the fast rise and decay of the electronic channel, the delayed lattice heating, and the long-lived low-frequency oscillation associated with the hybrid phonon-exciton mode.

Step 2: Background Subtraction and Oscillatory Isolation

To isolate the coherent part, we subtract a biexponential decay from the A_{1g}^* intensity dynamics, which captures the incoherent lattice thermalization background. The residual oscillatory component is then fitted using a damped cosine function of the form:

$$I_{\rm osc}(t) = A_{\rm osc} \cdot e^{-t/\tau_{\rm damp}} \cdot \cos\left(\frac{2\pi}{T}t + \phi\right), \tag{9}$$

where $A_{\rm osc}$ is the amplitude, $\tau_{\rm damp}$ the damping time, T the oscillation period, and ϕ the phase. This fit is performed for each fluence level, allowing a consistent comparison of the coherent mode evolution.

Step 3: Fluence Dependence and Suppression Threshold

The extracted oscillatory parameters are shown in Supplementary Fig. S2. At low fluence ($< 20\ \mu$J cm^{-2}), the hybrid mode remains sharp, with a well-defined amplitude and long damping time. As the fluence increases, the mode undergoes significant changes: (i) its amplitude increases initially (as more excitons are photoinduced), (ii) the damping time shortens, and (iii) the oscillation period lengthens, consistent with a softening behavior. At the highest fluence of 184 μJ/cm^2, the coherent oscillations become nearly undetectable in the Stokes response, indicating a breakdown of the hybrid mode and transition into a decohered, overheated lattice state.

Note 7 Long-time tail of the A_{1g}^* response at F$_c$

Figure S14 shows the long-time evolution of the A_{1g}^* Raman intensity for several pump fluences on a logarithmic scale. For low excitation densities, the signal consists of a sharp maximum around time zero followed by a rapid decay to the background within $\sim$ 10-15 ps, with no discernible long-time component. In contrast, at fluence ($F \approx 92\ \mu$J cm^{-2}) below the critical range of 100-200 μJ cm^{-2} defined in the main manuscript, the dynamics develop a pronounced plateau-like tail that remains nearly constant up to at least 60 ps, well longer than the decay times observed at lower and higher fluences. At higher fluences the decay becomes fast again, and the long-lived tail is absent. The persistence of this $\sim$ 60 ps component below F_c thus points to a transition in which the PLD remains trapped while the coherent motion of the A_{1g}^* phonon is strongly suppressed.

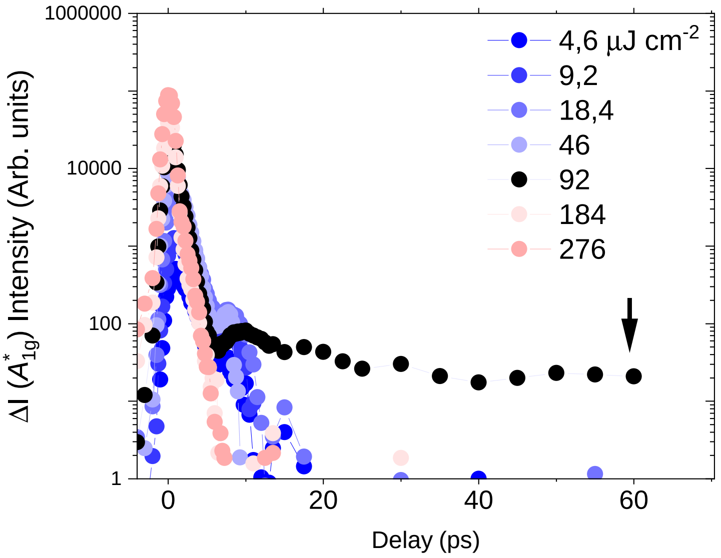

Figure S14: **Long-time Raman dynamics of the A_{1g}^* mode.** Time-resolved Raman intensity of the A_{1g}^* peak on a logarithmic scale for selected fluences. At low fluence the response decays within $\sim$ 10-15 ps, whereas below the critical fluence ($F \approx 92\ \mu$J/cm^2, black symbols) a long-lived tail persists beyond 60 ps (arrow). At higher fluences the signal again decays in about 20 ps without a pronounced long-time tail.

Note 8 Mode-Selective Nature of the Oscillation

To determine whether the coherent oscillation is specific to the A_{1g}^* mode or occurs generically across the Raman-active spectrum, we compare the dynamics of various Raman features, including the low-energy Raman continuum (LERC), the normal A_{1g} phonon mode, and the extracted χ^2 component. This analysis is presented in Fig. S15.

At low fluence (Fig. S15a), none of the non-CDW-associated signals, including χ^2, LERC, and A_{1g}, exhibit any hint of coherent oscillation. This confirms that the oscillation is not a

trivial optical response, but selectively tied to the CDW-associated A_{1g}^* mode.

At high fluence, as shown in Fig. S15b, the $A_{1\mathrm{g}}$ normal mode, despite its large amplitude, exhibits no oscillatory behavior. This stark contrast confirms the mode-selective character of the coherent dynamics and supports the picture of a hybrid phonon-exciton mode that undergoes catastrophic collapse above a critical fluence. Unlike the zone-center $A_{1\mathrm{g}}$ mode, A_{1g}^* originates from the L-point soft phonon directly tied to the CDW instability and selectively modulates the excitonic condensate via interlayer Ti-Se hybridization.

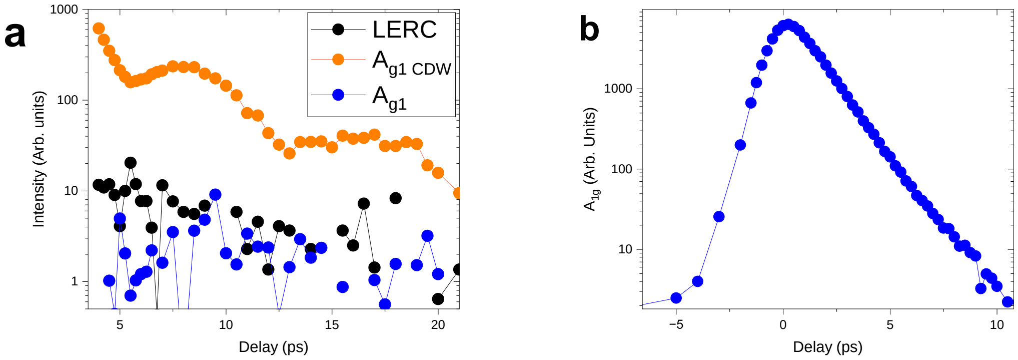

Figure S15: **Mode-selective emergence of coherent oscillations.** (a) Comparison of dynamics at low fluence showing the absence of oscillations in $A_{1\mathrm{g}}$, χ^2, and LERC, confirming the selectivity of the hybrid mode. (b) The strong $A_{1\mathrm{g}}$ signal remains featureless.

Note 9 Threshold Behavior in Anti-Stokes Raman Spectra

To further examine the robustness and collapse of the A_{1g}^* mode under increasing excitation, we present anti-Stokes Raman spectra at early time delays (1 ps and 2 ps) across a wide fluence range (Fig. S16). These spectra reveal a dramatic threshold behavior: while the A_{1g}^* mode softens and broadens continuously at intermediate fluences (up to $\sim 100\,\mu\mathrm{J/cm}^2$), it becomes strikingly rigid and unresponsive at higher fluences. Above this critical threshold, the spectral line shape of the A_{1g}^* mode remains virtually unchanged despite further increases in excitation density, signaling the saturation of its photoinduced softening. At the same time, the mode broadens as the fluence approaches and exceeds F_c, consistent with enhanced dephasing in the high-excitation regime. In addition, the damping time of the coherent $A_{1\mathrm{g}}^*$ oscillation extracted from the time-resolved reflectivity data decreases markedly with increasing fluence (Supplementary Note 11, Fig. S18e), consistent with the broadening of the $A_{1\mathrm{g}}^*$ feature and indicating enhanced dephasing of the CDW phonon at higher fluence. This points to enhanced incoherent relaxation of the surviving $A_{1\mathrm{g}}^*$ CDW phonon at higher fluence, most plausibly associated with the large transient phonon occupation, which can increase the phase space for anharmonic decay processes [16], with a possible additional contribution from electronic damping associated with the strongly excited nonequilibrium carrier background.

This saturation effect is a key experimental signature supporting the microscopic model introduced in the main text. Specifically, the model predicts a non-linear phonon-exciton coupling

that leads to a collapse of the hybrid order above a critical energy input. In this regime, the phonon ceases to soften because its coupling partner, the excitonic condensate, has already vanished, leaving the lattice component effectively "frozen" in its anharmonic state. The detailed spectral analysis of the A_{1g}^* line shape under these conditions is presented in Fig. 3 of the main manuscript.

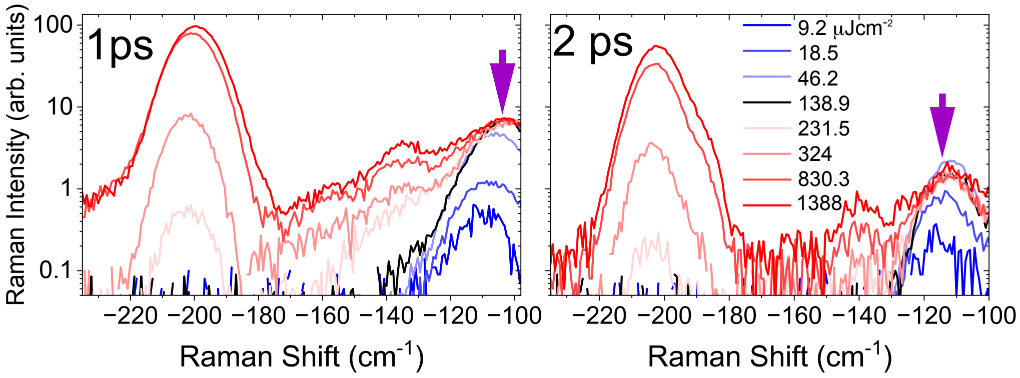

Figure S16: **Anti-Stokes Raman spectra at 1 ps and 2 ps for various fluences.** At low and intermediate fluences, the A_{1g}^* mode redshifts and broadens, reflecting phonon softening via coupling to the excitonic background. At high fluence ($> 100\,\mu$J/cm^2), the mode becomes rigid and unaffected by further excitation. This fluence threshold is consistent with the breakdown of excitonic coupling in the microscopic model.

Note 10 Estimation of Lattice Heating and Non-Thermal CDW Collapse

The pump fluence was calculated from the measured average power P, the repetition rate f, and the beam diameter at the sample. The spot size was obtained by imaging the reflected pump onto a CMOS camera and calibrating the image, yielding a beam diameter of 96 μm. From the pulse energy $E_\mathrm{p} = P/f$ and the illuminated area $A = \pi(D/2)^2$, where D is the pump-spot diameter, the fluence follows as $F = E_\mathrm{p}/A$. For $P = 1$ mW and $f = 150$ kHz this gives $F \approx 92\ \mu\mathrm{J\,cm^{-2}}$.

To estimate whether the collapse of CDW order can be attributed to laser-induced heating, we calculate the absorbed energy density using

$$E_\mathrm{abs} = \frac{F(1-R)}{\delta},$$

with $F = 476\,\mu\mathrm{J\,cm^{-2}}$, where $R \approx 0.6$ is the reflectivity and $\delta = 15$ nm is the penetration depth at the pump photon energy [17], yielding $E_\mathrm{abs} \approx 127\,\mathrm{mJ/cm^3}$. Converted per unit cell ($V_\mathrm{uc} \approx 100\,\mathrm{\AA}^3$), this corresponds to $\sim 37.4\,\mathrm{meV/u.c.}$

According to specific heat data from Ref. [18], heating the sample from 65 K to $T_\mathrm{CDW} = 202$ K requires $\sim$ 55-60 meV/u.c., indicating that the deposited energy at this fluence is insufficient to thermally drive the CDW transition.

Nonetheless, experimental data show a full collapse of the excitonic order at this fluence, confirming that the phase transition is of non-thermal origin and driven by photoinduced carrier density rather than lattice heating, in line with prior observations [19].

Note 11 Time-Resolved Reflectivity

To probe the ultrafast lattice dynamics and their fluence dependence in the CDW phase of $1T$-$TiSe_2$, we conducted time-resolved reflectivity (TRR) measurements at 5 K over a broad range of pump fluences. The transient reflectivity signal $\Delta R(t)$ exhibits a fast non-oscillatory electronic relaxation followed by coherent oscillations of the A_{1g}^* phonon mode associated with the CDW phonon.

The complete temporal response is modeled using a function comprising a biexponential electronic background and a damped coherent phonon oscillation: The damped coherent phonon oscillation is modeled as:

$$\Delta R(t) = \text{offset} + \left[A_1 e^{-\frac{(t-t_0)}{\tau_1}} + A_2 e^{-\frac{(t-t_0)}{\tau_2}}\right] * G(t) \cdot H(t) + A_{\text{osc}}\, e^{-\frac{(t-t_0)}{\tau_{\text{damp}}}} \cos\left(\frac{2\pi}{T}(t-t_0) + \phi\right) \cdot H(t-t_0), \tag{10}$$

where A_1 and A_2 are the amplitudes of the fast and slow background relaxations with decay constants τ_1 and τ_2, respectively. The background is convolved with the Gaussian instrument response $G(t)$ (FWHM ~130 fs), and multiplied by the Heaviside function $H(t)$. The last term models the coherent phonon oscillation with amplitude A_{osc}, damping τ_{damp}, period T, and phase ϕ.

Figure S17 displays the experimental data and fits for representative fluences. At low fluences, long-lived coherent oscillations of the A_{1g}^* mode are clearly observed. As fluence increases, the oscillations are strongly damped, and above ~180 μJ cm^{-2}, only the first oscillation cycle remains visible. The shaded regions highlight the time window of the first cycle.

The extracted fitting parameters are summarized in Fig. S18. The oscillation amplitude [panel (c)] initially increases and saturates around 150-200 μJ cm^{-2}, beyond which it begins to decrease. The frequency of the A_{1g}^* mode [panel (f)] exhibits a continuous redshift from 115 to ~105 cm^{-1}, consistent with phonon softening due to a weakened CDW potential. The damping time τ_{damp} [panel (e)] drops substantially, indicating increasing decoherence of the mode. These results are in line with time-resolved Raman spectroscopy and corroborate the critical fluence threshold beyond which the CDW order collapses.

To visualize the trend across the full fluence range, we construct a normalized grayscale map of $\Delta R(t)$ (Fig. S19). The first coherent oscillation, occurring around $t \approx 300$ fs, is evident across all fluences. However, at higher fluences, this oscillation becomes increasingly suppressed, appearing only as a faint cycle, consistent with overdamping of the A_{1g}^* mode.

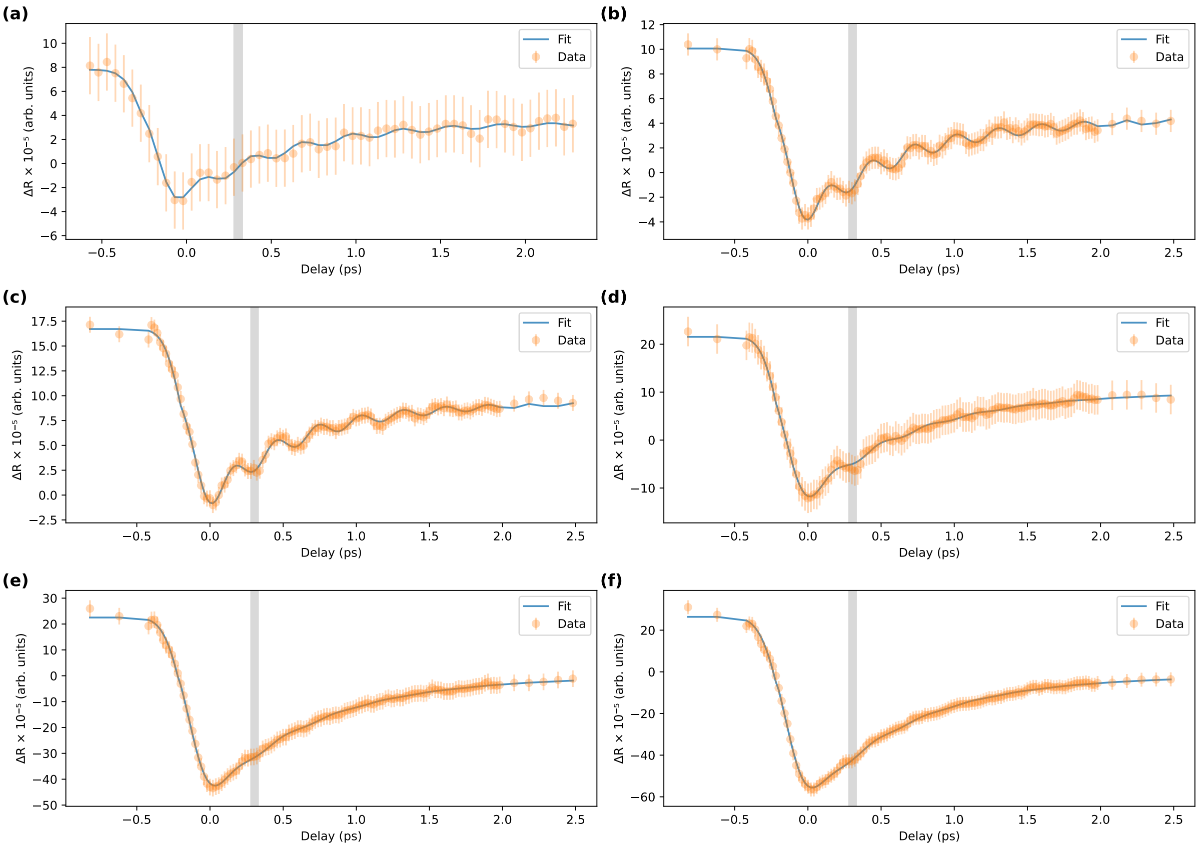

Figure S17: **Fluence-dependent TRR dynamics in *1T*-TiSe$_2$.** Time-resolved reflectivity $\Delta R(t)$ measured at 5 K for six representative fluences of (a) ∼50, (b) ∼60, (c) ∼90, (d) ∼120, (e) ∼180 and (f) ∼235 μJ cm^{-2}. Orange dots: measured data; blue lines: fit using the model in Eq. (S 10). Error bars indicate fitting uncertainties. The shaded region indicates the first oscillation cycle of the A^*_{1g} mode, which becomes increasingly damped with fluence.

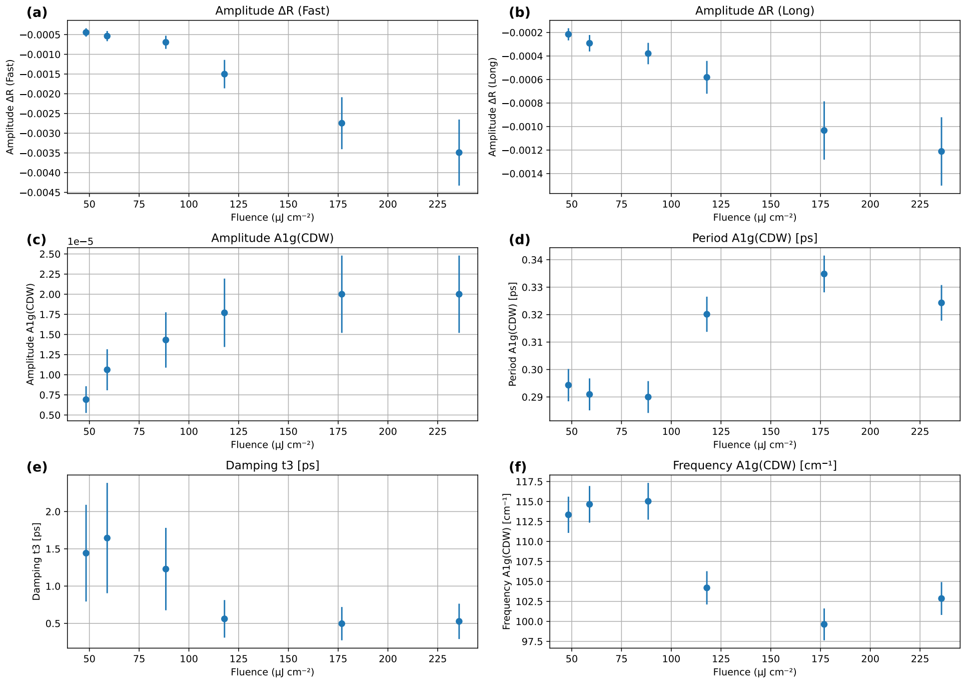

Figure S18: **Fluence dependence of fit parameters.** (a) and (b): amplitudes of the fast and long-lived background components. (c) Coherent A_{1g}^{*} oscillation amplitude. (d) Oscillation period. (e) Damping time. (f) Frequency of the A_{1g}^{*} mode. The results show redshift and increased damping with fluence, reflecting softening and decoherence of the CDW mode. Error bars indicate fitting uncertainties of the extracted parameters.

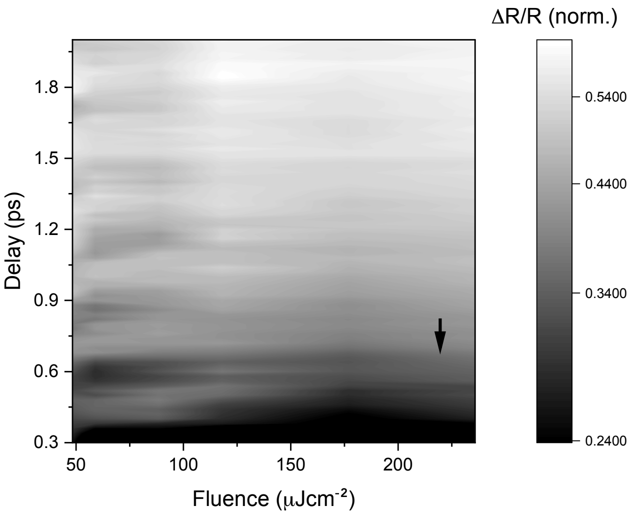

Figure S19: **Oscillation map of normalized** $\Delta R(t)$. Normalized TRR traces as a function of fluence. The first oscillation of the A_{1g}^{*} CDW phonon appears as a dark band near 300 and 600 fs. At fluences exceeding $\sim 180\,\mu\mathrm{J\,cm^{-2}}$, this band becomes faint and eventually vanishes, indicating selective damping of the mode.

Note 12 Comparison of critical fluences reported in ultrafast studies of $1T$-TiSe$_2$

To place the critical fluence identified in the present work in context, Table 1 summarizes representative fluence thresholds reported in previous ultrafast studies of $1T$-TiSe$_2$. Reported values are not expected to coincide exactly, because they depend on several factors, including the base temperature, the observable used to define the threshold, whether incident or absorbed fluence is quoted, and the probe geometry and sensitivity. With this distinction in mind, the literature shows a consistent trend rather than contradictory thresholds: electronic or excitonic signatures generally collapse at lower fluence, whereas structural suppression occurs at higher fluence. Our F_c falls within this established range and is fully consistent with our central observation that the coherent hybrid mode disappears at F_c while the folded A^*_{1g} phonon, and thus a substantial PLD, survives beyond it.

Supplementary Table 1: **Comparison of characteristic fluence thresholds reported in ultrafast studies of $1T$-TiSe$_2$.** The values listed here are approximate and should be interpreted in the context of the specific observable, temperature, and fluence definition used in each study.

Reference	Technique	Observable used to define F_c	F_{c1} (mJ cm^{-2})	F_{c2} (mJ cm^{-2})	Remark
Rohwer *et al.* [20]	trARPES	Collapse of long-range charge order / spectral reconstruction	~ 0.1	–	No single sharp F_c explicitly defined; lower-fluence regime already melts the CDW gap partially
Möhr-Vorobeva *et al.* [19]	Time-resolved x-ray diffraction / optical reflectivity	Structural CDW melting / suppression of coherent A_{1g} CDW amplitude mode	0.17–0.21	0.35	Temperature-dependent structural threshold; measurements near 140 K
Porer *et al.* [21]	Broadband THz spectroscopy	Electronic / excitonic suppression and high-fluence regime with persisting PLD	0.040	0.06–0.33	Excitonic order collapses before PLD; minimum temperature for PLD collapse is about 150 K

Supplementary Table 1 continued from previous page.

Reference	Technique	Observable used to define F_c	F_{c1} ($\mathrm{mJ\,cm^{-2}}$)	F_{c2} ($\mathrm{mJ\,cm^{-2}}$)	Remark
Hedayat *et al.* [22]	trARPES and tr-reflectivity	Excitonic / electronic collapse and partial gap dynamics	0.06	$\sim$ 0.1–0.2	Characteristic electronic threshold inferred from TRR and phonon-bottleneck analysis
Duan *et al.* [23]	trARPES and ultrafast electron diffraction	Exciton-condensation quench / PLD suppression	0.073	0.135	Two-threshold behavior in gap dynamics and structural response
Burian *et al.* [24]	Resonant / nonresonant tr-XRD	Orbital / excitonic order in the topmost layer and volume-averaged suppression	0.0123–0.033	$\sim$ 0.03–0.05	Separates near-surface orbital/electronic response from structural response
Cheng *et al.* [25]	Ultrafast electron diffraction	Collapse of interlayer coherence / dimensional crossover	$\sim$ 0.05	$\sim$ 0.1	Threshold regime associated with light-induced dimensional crossover
Huber *et al.* [26]	Ultrafast optical / photoemission study	Creation of a light-induced semimetallic state	$\sim$ 0.1	–	Strong-drive electronic transition; not always reported as two sharp thresholds
Cheng *et al.* [27]	Ultrafast electron diffraction	Topological defect formation / structural nonequilibrium regime	$\sim$ 0.1, $\sim$ 0.16	–	Approximate scales from the reported fluence window
Kurtz *et al.* [28]	Ultrafast electron diffraction	Full suppression of excitonic contribution to lattice distortion	$\sim$ 0.5	–	Complete suppression reached only at much higher fluence

Supplementary Table 1 continued from previous page.

Reference	Technique	Observable used to define F_c	F_{c1} ($\mathrm{mJ\,cm^{-2}}$)	F_{c2} ($\mathrm{mJ\,cm^{-2}}$)	Remark
This work	tr-Raman + tr-reflectivity	Collapse of coherent hybrid mode while folded A_{1g}^* persists	$\sim 0.1-0.2$	–	Coherent hybrid mode disappears before full collapse of the PLD

References

[1] Yuji Ikeda, Abel Carreras, Atsuto Seko, Atsushi Togo, and Isao Tanaka. Mode decomposition based on crystallographic symmetry in the band-unfolding method. *Phys. Rev. B*, 95:024305, Jan 2017.

[2] H. Hedayat, C. J. Sayers, D. Bugini, C. Dallera, D. Wolverson, T. Batten, S. Karbassi, S. Friedemann, G. Cerullo, J. Van Wezel, S. R. Clark, E. Carpene, and E. Da Como. Excitonic and lattice contributions to the charge density wave in 1T-$TiSe_2$ revealed by a phonon bottleneck. *Physical Review Research*, 1(2):023029, September 2019.

[3] J. A. Holy, K. C. Woo, M. V. Klein, and F. C. Brown. Raman and infrared studies of superlattice formation in Ti Se 2. *Physical Review B*, 16(8):3628–3637, October 1977.

[4] C. Monney, M. Puppin, C. W. Nicholson, M. Hoesch, R. T. Chapman, E. Springate, H. Berger, A. Magrez, C. Cacho, R. Ernstorfer, and M. Wolf. Revealing the role of electrons and phonons in the ultrafast recovery of charge density wave correlations in 1 T - $TiSe_2$. *Physical Review B*, 94(16):165165, October 2016.

[5] Timothy B. Boykin and Gerhard Klimeck. Practical application of zone-folding concepts in tight-binding calculations. *Physical Review B*, 71(11), 2005.

[6] Feipeng Zheng and Ji Feng. Electron-phonon coupling and the coexistence of superconductivity and charge-density wave in monolayer $nbse_2$. *Physical Review B*, 99(16), 2019.

[7] Yuji Ikeda, Abel Carreras, Atsuto Seko, Atsushi Togo, and Isao Tanaka. Mode decomposition based on crystallographic symmetry in the band-unfolding method. *Physical Review B*, 95(2), 2017.

[8] Atsushi Togo, Laurent Chaput, Terumasa Tadano, and Isao Tanaka. Implementation strategies in phonopy and phono3py. *J. Phys. Condens. Matter*, 35(35):353001, 2023.

[9] Atsushi Togo. First-principles phonon calculations with phonopy and phono3py. *J. Phys. Soc. Jpn.*, 92(1):012001, 2023.

[10] Jürgen Hafner. Ab-initio simulations of materials using vasp: Density-functional theory and beyond. *Journal of computational chemistry*, 29(13):2044–2078, 2008.

[11] Raffaello Bianco, Matteo Calandra, and Francesco Mauri. Electronic and vibrational properties of $TiSe_2$ in the charge-density-wave phase from first principles. *Physical Review B*, 92(9):094107, 2015.

[12] Stefan Grimme. Semiempirical gga-type density functional constructed with a long-range dispersion correction. *Journal of Computational Chemistry*, 27(15):1787–1799, 2006.

[13] R. B. Versteeg, J. Zhu, P. Padmanabhan, C. Boguschewski, R. German, M. Goedecke, P. Becker, and P. H. M. Van Loosdrecht. A tunable time-resolved spontaneous Raman spectroscopy setup for probing ultrafast collective excitation and quasiparticle dynamics in quantum materials. *Structural Dynamics*, 5(4):044301, July 2018.

[14] Hamoon Hedayat, Charles J Sayers, Arianna Ceraso, Jasper van Wezel, Stephen R Clark, Claudia Dallera, Giulio Cerullo, Enrico Da Como, and Ettore Carpene. Investigation of the non-equilibrium state of strongly correlated materials by complementary ultrafast spectroscopy techniques. *New Journal of Physics*, 23(3):033025, 2021.

[15] Kwangrae Kim, Hyun-Woo J Kim, Seunghyeok Ha, Hoon Kim, Jin-Kwang Kim, Jaehwon Kim, Junyoung Kwon, Jihoon Seol, Saegyeol Jung, Changyoung Kim, et al. Origin of the chiral charge density wave in transition-metal dichalcogenide. *Nature Physics*, 20(12):1919–1926, 2024.

[16] M Balkanski, RF Wallis, and E Haro. Anharmonic effects in light scattering due to optical phonons in silicon. *Physical Review B*, 28(4):1928, 1983.

[17] Ali Hussain Reshak and S. Auluck. Electronic and optical properties of the $1T$ phases of TiS2, TiSe2, and $TiTe_2$. *Physical Review B*, 68(24):245113, December 2003.

[18] RA Craven, FJ Di Salvo, and FSL Hsu. Mechanisms for the 200 k transition in $tise_2$: A measurement of the specific heat. *Solid State Communications*, 25(1):39–42, 1978.

[19] E. Möhr-Vorobeva, S. L. Johnson, P. Beaud, U. Staub, R. De Souza, C. Milne, G. Ingold, J. Demsar, H. Schaefer, and A. Titov. Nonthermal Melting of a Charge Density Wave in $TiSe_2$. *Physical Review Letters*, 107(3):036403, July 2011.

[20] Timm Rohwer, Stefan Hellmann, Martin Wiesenmayer, Christian Sohrt, Ankatrin Stange, Bartosz Slomski, Adra Carr, Yanwei Liu, Luis Miaja Avila, Matthias Kalläne, Stefan Mathias, Lutz Kipp, Kai Rossnagel, and Michael Bauer. Collapse of long-range charge order tracked by time-resolved photoemission at high momenta. *Nature*, 471(7339):490–493, March 2011.

[21] M. Porer, U. Leierseder, J.-M. Ménard, H. Dachraoui, L. Mouchliadis, I. E. Perakis, U. Heinzmann, J. Demsar, K. Rossnagel, and R. Huber. Non-thermal separation of electronic and structural orders in a persisting charge density wave. *Nature Materials*, 13(9):857–861, September 2014.

[22] H. Hedayat, C. J. Sayers, D. Bugini, C. Dallera, D. Wolverson, T. Batten, S. Karbassi, S. Friedemann, G. Cerullo, J. Van Wezel, S. R. Clark, E. Carpene, and E. Da Como. Excitonic and lattice contributions to the charge density wave in 1T-$TiSe_2$ revealed by a phonon bottleneck. *Physical Review Research*, 1(2):023029, September 2019.

[23] Shaofeng Duan, Yun Cheng, Wei Xia, Yuanyuan Yang, Chengyang Xu, Fengfeng Qi, Chaozhi Huang, Tianwei Tang, Yanfeng Guo, Weidong Luo, Dong Qian, Dao Xiang, Jie Zhang, and Wentao Zhang. Optical manipulation of electronic dimensionality in a quantum material. *Nature*, 595(7866):239–244, July 2021.

[24] Max Burian, Michael Porer, Jose R. L. Mardegan, Vincent Esposito, Sergii Parchenko, Bulat Burganov, Namrata Gurung, Mahesh Ramakrishnan, Valerio Scagnoli, Hiroki Ueda, Sonia Francoual, Federica Fabrizi, Yoshikazu Tanaka, Tadashi Togashi, Yuya Kubota, Makina Yabashi, Kai Rossnagel, Steven L. Johnson, and Urs Staub. Structural involvement in the

melting of the charge density wave in 1 T-$TiSe_2$. *Physical Review Research*, 3(1):013128, February 2021.

[25] Yun Cheng, Alfred Zong, Jun Li, Wei Xia, Shaofeng Duan, Wenxuan Zhao, Yidian Li, Fengfeng Qi, Jun Wu, Lingrong Zhao, Pengfei Zhu, Xiao Zou, Tao Jiang, Yanfeng Guo, Lexian Yang, Dong Qian, Wentao Zhang, Anshul Kogar, Michael W. Zuerch, Dao Xiang, and Jie Zhang. Light-induced dimension crossover dictated by excitonic correlations. *Nature Communications*, 13(1):963, February 2022.

[26] Maximilian Huber, Yi Lin, Giovanni Marini, Luca Moreschini, Chris Jozwiak, Aaron Bostwick, Matteo Calandra, and Alessandra Lanzara. Ultrafast creation of a light-induced semimetallic state in strongly excited 1T-$TiSe_2$. *Science Advances*, 10(19):eadl4481, May 2024.

[27] Yun Cheng, Alfred Zong, Lijun Wu, Qingping Meng, Wei Xia, Fengfeng Qi, Pengfei Zhu, Xiao Zou, Tao Jiang, Yanfeng Guo, Jasper Van Wezel, Anshul Kogar, Michael W. Zuerch, Jie Zhang, Yimei Zhu, and Dao Xiang. Ultrafast formation of topological defects in a two-dimensional charge density wave. *Nature Physics*, 20(1):54–60, January 2024.

[28] Felix Kurtz, Tim N. Dauwe, Sergey V. Yalunin, Gero Storeck, Jan Gerrit Horstmann, Hannes Böckmann, and Claus Ropers. Non-thermal phonon dynamics and a quenched exciton condensate probed by surface-sensitive electron diffraction. *Nature Materials*, 23(7):890–897, July 2024.
