# Supplementary material for: Tracking the catastrophic collapse of hybrid exciton-phonon order in a quantum material
Source: Nat Commun. 2026 Jul 30;17:7607. doi: 10.1038/s41467-026-75887-9 (PMC13424097; doi:10.1038/s41467-026-75887-9)
Supplement: Supplementary file 2 — Peer Review file [file 41467_2026_75887_MOESM2_ESM.pdf]

nature portfolio

Peer Review File

Tracking the Catastrophic Collapse of Hybrid Exciton-Phonon Order in a Quantum Material

Corresponding Author: Dr Hamoon Hedayat

This file contains all reviewer reports in order by version, followed by all author rebuttals in order by version.

Version 0:

Reviewer comments:

Reviewer #1

(Remarks to the Author)
The authors employ pump-probe techniques - Raman scattering and reflectivity measurements - to uncover for the first time a new hybrid exciton-phonon mode in the charge-density wave phase of the layered vdW compound 1T-TiSe2, manifested in the form of a locked phason.
Based on its thermal evolution in equilibrium, its temporal evolution, and its out-of-equilibrium thermal behavior, and based on the disparate evolution of the CDW-related periodic lattice distortion, the authors demonstrate that the charge-density wave phase in 1T-TiSe2 involves a cooperative interplay between electronic and lattice degrees of freedom.

The impressive results presented in this study are of high quality, and together with the discussion and theoretical support constitute an overall sound study.

There are a few issues for the authors could comment on, and improve or revise their work accordingly, before the manuscript can be accepted for publication.

1) In contrast to conventional CDW phases, the authors characterize the CDW in the title compound to emerge through a discrete breaking of symmetries rather than a continuous breaking of symmetries, thereby gapping out the phason. This scenario is somewhat reminiscent of other exotic CDW phases, as observed e.g. in rare-earth tritellurides (see, e.g., Nature 606, 896 and Nat. Phys. 21, 1578), where the breaking of different symmetries gives rise to an amplitudon characterized as an axial Higgs mode. Can the authors comment on the commonalities / differences between these CDW formation processes? Since the experimental results are based on Raman spectroscopy, can the authors find the existence (or absence) of subtle deviations from the expected Raman tensors in 1T-TiSe2?
On a broader note, it would be helpful if the authors could contextualize the relevance of their findings for other systems where charge-density wave order underlies unconventional processes.

2) Can the authors comment on the symmetry of the locked phason? Would it inherit the A1g symmetry of the CDW phonon, or be of lower symmetry?

3) Please show the color contour plot in Fig. 2(b) for extended times to illustrate the oscillation of the A1g* phonon up to 20 ps in the as-measured data.

4) The A1g mode at about 200 cm-1 appears to be imprinted with a faster, sub-ps modulation in intensity, as seen in Fig. 2(c). Please comment on the potential origins of this oscillation.

5) Previous studies have found that the cooperative nature of lattice and electronic instabilities in 1T-TiSe2 gives rise to a chiral charge-density wave (see, e.g., Nat. Phys. 20, 1919). What are the potential implications of chiral vs. non-chiral order on the results presented by the authors?

6) The ratio of Stokes to anti-Stokes scattering intensity is used to extract information about the temperature of the system during the measurement process. While this procedure is well-established for conventional bosonic excitations in systems where time-reversal symmetry is preserved, it may become invalid for selected excitations in systems where time-reversal symmetry is broken, such as charge-density wave phases with chiral electronic order. How can the authors rule out such

contributions when estimating the temperature of subsystems based on Stokes / anti-Stokes ratios?

7) minor issues / inconsistencies:
- Fig. 1, axis label in (c) should read "Temperature"
- Ni2TaSe5 should read Ta2NiSe5
- temperatures are sometimes referred to in the text as 300 K, yet labeled in the figures as 295 K.
- the statement "anti-Stokes TR-Raman, which more directly probes the phonon occupation" sounds a bit odd. Perhaps the authors meant to say that anti-Stokes TR-Raman allows for a more accurate determination of phonon occupation in the low-temperature limit?

Reviewer #2

(Remarks to the Author)
Many ultrafast experiments focus on 1-$TiSe_2$, widely regarded as a benchmark system for exploring nonequilibrium charge-density-wave and excitonic physics. While its band structure is relatively simple, complex many-body interactions create a wealth of intertwined excitation and relaxation channels. This complexity yields intriguing experimental findings, yet their interpretations frequently remain debated.

The authors report on a long-period (~ 0.13 THz) modulation in the intensity of the CDW A*1g mode. This 0.13 THz oscillation disappears at a critical fluence, Fc, although the spectral weight of the A*1g phonon persist above this threshold. They interpret the 0.13 THz mode as a locked phason of a hybrid exciton-phonon CDW and define Fc as the excitation fluence at which the locked phason amplitude collapses, marking the loss of coherent excitonic order while the lattice distortion remains.

The experiment was done to a high standard, and the results are impressive. To justify their interpretation, the authors review and rule out several alternatives. I believe, however, some important ones may have been overlooked.

1) Recent ultrafast experiments using transient absorption spectroscopy [Huang et al., Nanotechnology 34, 235707 (2023)] and electron diffraction dynamics [Karam et al., ACS Photonics 5 1228–34 (2018)] on exfoliated 1T-TiSe2 have revealed similar low-frequency oscillations, which arise from out-of-plane acoustic breathing modes and are attributed to the excitation of longitudinal acoustic phonons quantized by the sample thickness. For 14-nm thick 1T-TiSe2, the transient absorption oscillation frequency is 0.147 THz, which is quantitively very close to the 0.13 THz modulation of the CDW A*1g mode observed by Hedayat et al., along with their 15 nm penetration depth estimate given in the supplementary information (Note 10). In this scenario, strong electron–phonon coupling in excited TiSe2 is governed by Kohn anomalies, affecting electron transport and energy relaxation in the layered structure. The mode-selective nature of the Raman oscillations may relate to the specific character of the CDW mode, which emerges due to the acoustic branch folding (Fig. 1e).

2) In this context, impurities and lattice pinning can significantly influence interlayer coupling. Huang et al. (see Ref. above) note the inevitable presence of intrinsic intercalated Ti atoms in the van der Waals gap of 1T-TiSe2 and propose that photoexcited electrons are rapidly trapped by these Ti defects, forming color centers (small polarons). They further propose that the density of intercalated Ti atoms (2.2 × 10^13 cm–2 per Se–Ti–Se layer) sets a threshold pump fluence. Under higher pump fluence, carrier congestion impedes the migration of excessive carriers. Could the photogenerated carrier density at Fc also be comparable to the Ti defect density?

3) The authors assess and argue against a thermally induced CDW collapse due to lattice overheating at pump fluences exceeding Fc. Their assessment in SI Note 10 shows that the corresponding accumulated energy is insufficient yet comparable to that of the CDW thermal transition, missing by a few times or merely tens of percent. In such close estimates, it is crucial to account for the spatial inhomogeneity of the excitation, both laterally and through the sample depth. Such spatial excitation inhomogeneity may indeed be the reason why the 0.13 THz mode vanishes at Fc, even though the A*1g phonon persists well beyond this threshold, rendering one of the authors' key arguments unconvincing.

4) The authors note that the low-energy Raman continuum (LERC) may reflect excitonic fluctuations but avoid further discussion in this respect. It is worth citing another key reference alongside Refs 49 and 50: Kim et al., Nature Commun. 12, 1969 (2021). It remains ambiguous whether the authors consider the attribution of the LERC to exciton fluctuations unconvincing, or whether its behavior in their experiments conflicts with their own interpretation framework.

In conclusion, the authors propose an appealing and fashionable interpretation of the impressive experimental results, grounded in theoretical descriptions of non-equilibrium processes in idealized structures. Nevertheless, I do not find their case sufficiently compelling to rule out potential contributions from inhomogeneous spatial excitation, intrinsic defects, or local lattice overheating to the observed non-equilibrium response. I cannot recommend this work for publication in Nature Communications.

Additional note: To prepare the article for future submission, authors should thoroughly edit the Supplementary Information to eliminate typos and inconsistencies: CDW-CWD, Fig.10a vs Fig.10(left), Jcm-1 vs. Jcm-2, the same “d” in “A” and “E_abs”, etc.

Reviewer #3

(Remarks to the Author)
This manuscript reports a compelling study on phonon-exciton coupling in $TiSe_2$, employing the advanced experimental technique of time-resolved Raman spectroscopy. The comparison of Raman spectra pre- and post-optical pumping yields valuable insights into phonon excitation dynamics. While the work engages with intriguing physical phenomena, the interpretation would benefit from addressing several key points to solidify the conclusions.

1. For Figure 2, please clarify if the legend in panel (a) applies universally to panels (b) and (c). A similar clarification is needed for Figure 4a-d. Explicitly labeling the ΔI values in the relevant legends would also immediately enhance readability.

2. The oscillatory decay of the A1g* phonon intensity is attributed to coherent collective oscillations from phonon-exciton coupling. An alternative explanation could involve phonon-phonon scattering processes. Please discuss whether this mechanism was considered and, if feasible, comment on the possibility of incorporating it into the theoretical model to strengthen the argument for excitonic coupling.

3. The identified critical fluence (Fc) is linked to a phase transition. How does this threshold compare with values reported in prior studies on $TiSe_2$ or analogous materials? Providing this context would help position the new findings within the existing literature.

4. In Figure 3, the saturation of the anti-Stokes peak is suggested to correspond to a metastable state. Please elaborate on the proposed connection between saturation and metastability. Specifically, explain the physical rationale for why the phonon population would saturate under these conditions rather than continue to evolve.

5. Significant phonon broadening is observed in Figures 3c and 3e, occurring after frequency stabilization. Could this broadening be attributed to enhanced phonon scattering? If so, please speculate on the potential scattering mechanisms (e.g., increased electron-phonon or defect-phonon scattering) that may become active in this regime.

6. Figure 4d shows a less pronounced intensity change for the A1g mode compared to the A1g* mode. Does this imply a different thermal activity? Please discuss whether the A1g phonon becomes more active with increasing temperature, or if the A1g* phonon is simply less susceptible to thermal effects, and the implications for the coupling mechanism.

This study presents a rich and valuable dataset that clearly advances the investigation of coupled dynamics in $TiSe_2$. Addressing the points above to deepen the interpretation and discussion will significantly strengthen the manuscript and solidify its contribution to the field.

Reviewer #4

(Remarks to the Author)
I co-reviewed this manuscript with one of the reviewers who provided the listed reports. This is part of the Nature Communications initiative to facilitate training in peer review and to provide appropriate recognition for Early Career Researchers who co-review manuscripts.

Version 1:

Reviewer comments:

Reviewer #1

(Remarks to the Author)
I am glad to see that the authors responded to all raised issues, sharing their detailed comments and explanations. Based on these responses together with the revisions of the main text and the supplementary I am now happy to recommend this manuscript for a publication in Nature Communications.

Reviewer #2

(Remarks to the Author)
In their response, the authors carefully addressed the reviewers' comments. Their detailed, point-by-point replies and the subsequent revisions further enhanced the manuscript's quality. In particular, they critically examined my comment regarding thickness-quantized out-of-plane acoustic breathing modes, as experimentally observed in thin $TiSe_2$ flakes — an effect that had been overlooked in their initial analysis. They also engaged with, and partly accepted, my comment regarding spatially inhomogeneous excitation and possible near-surface melting of the CDW in $TiSe_2$.

Despite the extensive list of counterarguments provided, I cannot agree with some of them and find remaining inconsistencies:

- The authors acknowledge that "in thin flakes, breathing mode oscillations manifest as broadband optical modulations in the transient dynamics and are observed at room temperature in the normal phase."

- Citing room-temperature neutron scattering measurements by Stirling et al., they estimate that "a fundamental breathing mode at 0.13 THz would strictly require a mechanically bounded slab of approximately 10.5 nm thickness with two free

surfaces."

- However, thickness quantization does not necessarily require two mechanically exfoliated free surfaces — a sharp phase boundary can serve as the rear surface. The CDW phase is characterized not only by different structural periodicity but, more importantly, by dramatically different carrier scattering and density, CDW-induced gap features, and plasmon responses, all reflecting substantial band structure modifications.

In light of these points, might one not reasonably hypothesize that, under optical pumping within a very specific and narrow range of fluence, a sharp phase interface forms, and that a ~10 nm thick layer of the high-temperature phase accounts for the observed 0.13 THz modulation of the optical response?

Although I admit that this mechanism may sound exotic, I find the authors' proposed assignment of the 0.13 THz modulation to a locked phason no less exotic, particularly in light of possible phase coexistence arising from significant spatial inhomogeneity of both the pump and the probe. While this ambiguity complicates a definitive assessment of the manuscript, it highlights the unusual character and relevance of the experimental findings. I believe this work merits publication in some form. Whether it meets the specific criteria of Nature Communications is ultimately a matter for the editors to decide, in consideration of the feedback from all reviewers.

Reviewer #3

(Remarks to the Author)
All my previous concerns have been satisfactorily resolved. I support the publication of this work.

Reviewer #4

(Remarks to the Author)
I co-reviewed this manuscript with one of the reviewers who provided the listed reports. This is part of the Nature Communications initiative to facilitate training in peer review and to provide appropriate recognition for Early Career Researchers who co-review manuscripts.

Open Access This Peer Review File is licensed under a Creative Commons Attribution 4.0 International License, which permits use, sharing, adaptation, distribution and reproduction in any medium or format, as long as you give appropriate credit to the original author(s) and the source, provide a link to the Creative Commons license, and indicate if changes were made.
In cases where reviewers are anonymous, credit should be given to 'Anonymous Referee' and the source.
The images or other third party material in this Peer Review File are included in the article’s Creative Commons license, unless indicated otherwise in a credit line to the material. If material is not included in the article’s Creative Commons license and your intended use is not permitted by statutory regulation or exceeds the permitted use, you will need to obtain permission directly from the copyright holder.
To view a copy of this license, visit https://creativecommons.org/licenses/by/4.0/

We thank the reviewers for their careful, detailed, and constructive reports. We are grateful for their thoughtful assessment of the manuscript and for the many helpful suggestions, which have helped us improve the clarity and strength of the revised version.

We particularly appreciate that all three reviewers recognized the quality and significance of the work. Reviewer #1 describes the results as “impressive” and “of high quality,” and judges the study overall “sound,” highlighting that our pump-probe Raman and reflectivity measurements uncover a new hybrid exciton-phonon mode in the CDW phase and provide evidence for cooperative interplay between electronic and lattice degrees of freedom. Reviewer #2 also notes that the experiment was performed “to a high standard” and that the results are “impressive,” while raising important alternative physical scenarios that helped us sharpen and strengthen the central interpretation. Reviewer #3 likewise describes the manuscript as “compelling,” notes that the dataset is “rich and valuable,” and states that it “clearly advances” the investigation of coupled dynamics in $TiSe_2$, while recommending targeted clarifications to further strengthen the presentation.

In this revision, we have therefore focused on making the central interpretation as robust as possible, clarifying the physical picture, addressing the alternative scenarios raised by the reviewers, and improving the presentation throughout the manuscript and Supplementary Information.

All changes are provided in the revised manuscript with tracked changes and color highlighting, as requested. Below we provide a point-by-point response to all comments.

REVIEWER #1 (Remarks to the Author)

The authors employ pump-probe techniques - Raman scattering and reflectivity measurements - to uncover for the first time a new hybrid exciton-phonon mode in the charge-density wave phase of the layered vdW compound 1T- $TiSe_2$, manifested in the form of a locked phason.

Based on its thermal evolution in equilibrium, its temporal evolution, and its out-of-equilibrium thermal behavior, and based on the disparate evolution of the CDW-related periodic lattice distortion, the authors demonstrate that the charge-density wave phase in 1T-$TiSe_2$ involves a cooperative interplay between electronic and lattice degrees of freedom.

The impressive results presented in this study are of high quality, and together with the discussion and theoretical support constitute an overall sound study.

There are a few issues for the authors could comment on, and improve or revise their work accordingly, before the manuscript can be accepted for publication.

Response: We thank the reviewer for their positive evaluation and insightful suggestions.

1) In contrast to conventional CDW phases, the authors characterize the CDW in the title compound to emerge through a discrete breaking of symmetries rather than a continuous breaking of symmetries, thereby gapping out the phason. This scenario is somewhat reminiscent of other exotic CDW phases, as observed e.g. in rare-earth tritellurides (see, e.g., Nature 606, 896 and Nat. Phys. 21, 1578), where the breaking of different symmetries gives rise to an amplitudon characterized as an axial Higgs mode. Can the authors comment on the commonalities / differences between these CDW formation processes? Since the experimental results are based on Raman spectroscopy, can the authors find the existence (or absence) of subtle deviations from the expected Raman tensors in 1T-$TiSe_2$?

On a broader note, it would be helpful if the authors could contextualize the relevance of their findings for other systems where charge-density wave order underlies unconventional processes.

Response: We thank the reviewer for this insightful suggestion. The comparison with the recent Raman studies on rare-earth tritellurides (RT_3) is indeed very useful, because in both cases the central message is that collective modes provide symmetry information that is not captured by the ordering wavevector alone. In that sense, the commonality is that both systems go beyond the conventional single-component Peierls picture: the low-energy collective response reflects the internal structure of the ordered state and reveals that the broken-symmetry phase is more complex than a simple lattice modulation.

The important difference, however, is that the relevant collective sector is not the same. In RTe_3, the reported axial Higgs mode is an amplitude-sector excitation whose Raman tensor directly reflects the unconventional symmetry structure of a multi-component CDW. In our case, by contrast, the key excitation is a phase-sector mode: the 0.13 THz response is consistent with a locked phason, i.e. a gapped phase mode of the hybrid exciton-phonon order. Its gap does not arise from the same symmetry mechanism as an axial Higgs mode, but from locking of the excitonic phase by coupling to the lattice. Thus, while both systems illustrate how unconventional CDW order can be diagnosed through collective excitations, the underlying physics is different: RTe_3 highlights unusual amplitude-mode symmetry, whereas 1T-$TiSe_2$ reveals phase locking in a cooperative exciton-phonon condensate.

Regarding Raman tensors and selection rules, we now add a polarization-resolved static Raman measurement at 100 K (Fig. S2) to the SI, together with a short symmetry analysis (SI Section S2). The data confirm the expected conventional symmetry [PHYSICAL REVIEW B, 16, 8 1977] : the A_{1g} and folded A_{1g}* modes dominate in parallel (XX), while the E_g modes appear in both XX and crossed (XY) polarizations, consistent with the standard D3d selection rules. This also motivates our TR-Raman geometry: we work in XX geometry to maximize sensitivity to the A_{1g}* mode and thereby resolve its dynamics and low-frequency modulations in CDW phase.

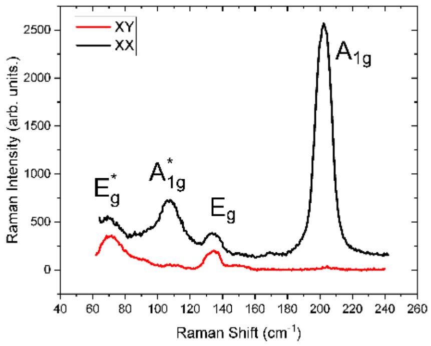

Figure R1. Polarization-resolved static Raman spectra of 1T-$TiSe_2$ at 100 K in parallel (XX, black) and crossed (XY, red) geometries. The data confirm the expected D3d selection rules: the A_{1g} and folded A_{1g}* modes are strongest in XX, while the E_g and folded E_g* modes are visible in both XX and XY. This supports the use of the XX geometry in the TR-Raman measurements to maximize sensitivity to the folded A_{1g}* mode.

2) Can the authors comment on the symmetry of the locked phason? Would it inherit the A_{1g} symmetry of the CDW phonon, or be of lower symmetry?

Response: We thank the reviewer for this important question. Microscopically, it is a collective mode of the phase fluctuation of the coupled exciton-phonon order parameter, i.e. the gapped phase mode that results when the excitonic phase sector is locked by coupling to the lattice. Its natural symmetry is

therefore that of the phase sector of the condensate. In the experiment, however, this collective mode becomes visible only through its coupling to the folded A_{1g}* phonon, and it is detected exclusively in that channel. Thus, what our data establishes directly is that the projection of the locked phason is manifested in the A_{1g} dynamics in our measurement geometry, not that the full microscopic mode is in itself an A_{1g} symmetry excitation.

3) Please show the color contour plot in Fig. 2(b) for extended times to illustrate the oscillation of the A_{1g}* phonon up to 20 ps in the as-measured data.

Response: We extended the plot to 20 ps using the as-measured data and included the extended data in Fig2 of the revised manuscript as shown in Fig R2.

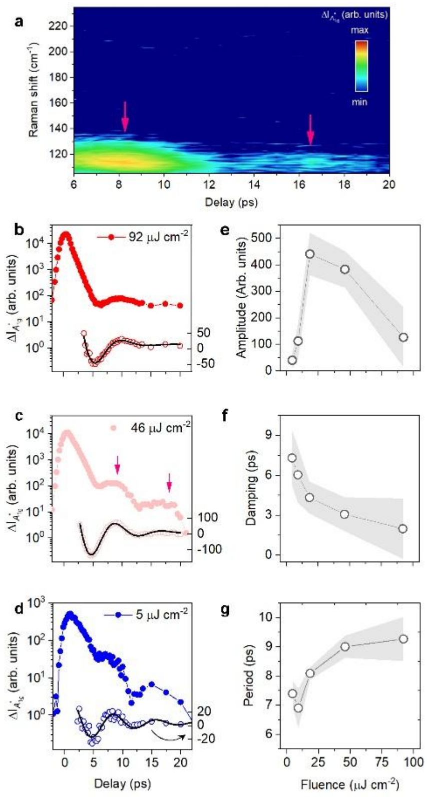

Figure R2. Revised layout of Extended Data Fig. 2 in the main manuscript. The newly added panel (a) shows the time-resolved Raman map of the photoinduced A^*_{1g} response at low temperature over an extended delay range (6-20 ps), corresponding to the same dataset shown in Fig. 2b of the main manuscript.

4) The A_{1g} mode at about 200 cm-1 appears to be imprinted with a faster, sub-ps modulation in intensity, as seen in Fig. 2(c). Please comment on the potential origins of this oscillation.

Response: We thank the reviewer for the careful inspection of our data. We have evaluated the intensity features occurring at early delays in Fig. 2(b) and Fig. 2(c), and we provide here several reasons why these represent experimental noise rather than a physical sub-picosecond modulation: 1. Spectral Distribution:

these intensity spikes are spread uniformly across all measured frequencies (white dashed box in Fig R3 left panel). A physical modulation from a phonon mode, for example, would typically be localized or follow specific symmetry-dependent selection rules, whereas this feature appears as a broadband fluctuation. 2. Absence of Coherent Periodicity: there is no discernible modulation frequency or stable phase pattern; instead, the signal is characterized by stochastic spikes that can be described by a single exponential decay plus random noise (Fig R3- right panel). 3. Experiment Specificity: this noise is specific to this individual measurement. Other datasets in the manuscript, such as the one presented in Figure 4, do not exhibit these spikes, further confirming they are not an intrinsic property of the material excitation spectrum.

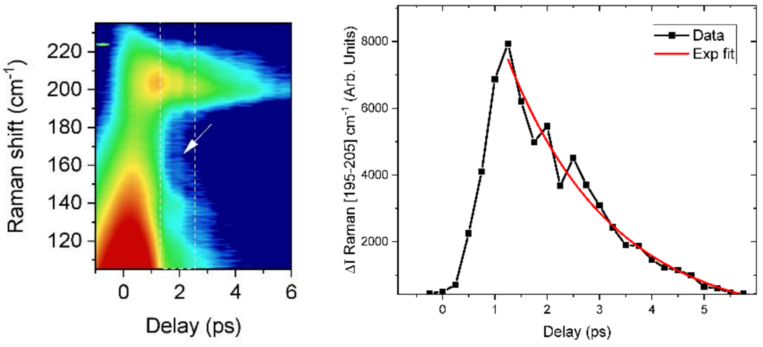

Figure R3. Early-time intensity fluctuations are attributable to experimental noise rather than a genuine sub-picosecond modulation. Left panel: Time-resolved Raman map in the spectral region containing the normal-phase A_{1g} mode. The intensity spikes highlighted by the dashed box are distributed broadly over the spectrum rather than being confined to a specific phonon response, which is inconsistent with a mode-selective coherent modulation. Right panel: Delay trace integrated over the A_{1g} spectral window (195–205 cm^{-1}). The data are well described by a simple exponential decay with superimposed stochastic fluctuations, with no discernible periodicity or stable phase. Together, these observations support the conclusion that the apparent early-time spikes arise from experimental noise specific to this measurement and do not represent an intrinsic sub-picosecond oscillation.

5) Previous studies have found that the cooperative nature of lattice and electronic instabilities in 1T-$TiSe_2$ gives rise to a chiral charge-density wave (see, e.g., Nat. Phys. 20, 1919). What are the potential implications of chiral vs. non-chiral order on the results presented by the authors?

Response: We thank the reviewer for raising this important point regarding chiral CDW order in 1T-$TiSe_2$. The recent work by Kim et al. (Nat. Phys. 20, 1919) is highly relevant. That study demonstrates that chirality originates from "charge-lattice symmetry frustration," where electronic and lattice modulations follow distinct irreducible representations, inducing subtle lattice distortions that lower the symmetry.

However, the emergence of chirality does not alter the fundamental conclusions of our study. Kim et al. confirm that the A_{1g}* mode remains the dominant representation of the structural distortion. Since our 0.13 THz locked phason is detected exclusively through the modulation of this primary A_{1g}* channel, our experiment is sensitive to the fundamental cooperative coupling rather than the secondary, chiral symmetry-breaking distortions. Even if chiral order were present, its role would be to renormalize the detailed symmetry and polarization properties of the “other” folded phonons, such as the splitting of E_g doublets reported in Kim et al. (Nat. Phys. 20, 1919) . Our pulsed TR-Raman setup is optimized for tracking ultrafast non-equilibrium dynamics and does not have the spectral resolution required to resolve the subtle folded-mode E_g splittings discussed in the chiral CDW works. A full disentangling of the role of

chirality in the ultrafast phononic response would require a dedicated study beyond the scope of the present work. Therefore, while chirality is an important structural refinement, our dynamical signatures of A_{1g}* channel remain robust spectroscopic fingerprints of the hybrid order, independent of these secondary chiral distortions. We now discuss this point explicitly in the revised manuscript: "Any possible chiral refinement of the CDW order is not expected to alter this interpretation, since the collapse of the low-frequency mode together with the survival of the folded A_{1g}* response is not explained by a small symmetry refinement affecting mainly folded E_g mode features (Nat. Phys. 20, 1919).

6) The ratio of Stokes to anti-Stokes scattering intensity is used to extract information about the temperature of the system during the measurement process. While this procedure is well-established for conventional bosonic excitations in systems where time-reversal symmetry is preserved, it may become invalid for selected excitations in systems where time-reversal symmetry is broken, such as charge-density wave phases with chiral electronic order. How can the authors rule out such contributions when estimating the temperature of subsystems based on Stokes / anti-Stokes ratios?

Response: We thank the reviewer for raising this important point. We agree that the extraction of phonon occupation from the Stokes/anti-Stokes ratio assumes that the two channels are related by the usual reciprocal Raman matrix element, and that this relation could in principle be modified if strong symmetry-related corrections were present.

However, we believe such effects do not compromise our temperature analysis here. First, the recent chiral-CDW Raman study mentioned by the Reviewer (Kim et al., Nat. Phys. 2024) itself uses the Stokes/anti-Stokes ratio to estimate laser heating, indicating that the possible chirality-related correction is not expected to invalidate this procedure in practice when the chiral contribution is not significant.

Second, our temperature analysis is performed for the folded A_{1g}* phonon, which is the primary structural CDW mode and not itself the chiral mode discussed in the chirality literature. Thus, even if chirality affects other phonon modes or their detailed symmetry classification, it is not expected to strongly distort the temperature extracted from the A_{1g}* Stokes/anti-Stokes ratio.

Third, any chirality-related correction would enter our temperature extraction through a transient asymmetry of the Raman matrix elements in the Stokes and anti-Stokes channels, that is, through the assumption that both channels share the same underlying susceptibility dynamics apart from the phonon occupation factor. Such a correction is therefore a Raman-tensor effect, not a delayed phonon-population effect. In our analysis, we explicitly separate the susceptibility contribution from the phonon-population response (see Note 5 in the Supplementary Information). The extracted susceptibility change is confined to the earliest delays, within the first 1 ps and essentially on the instrumental-response timescale, where the photoinduced electronic redistribution and any associated matrix-element renormalization are expected to be strongest. By contrast, the maximum A_{1g}* phonon temperature occurs later, around 2 ps, after this fast electronic response has already relaxed and the phonon population has built up with delay (see Figure R3 and Fig. 8 in the SI). Therefore, even if a small chirality-induced deviation from the equal-matrix-element assumption were present at early times, it would mainly affect the earliest part of the extracted response and weakly renormalize the absolute temperature scale. This temporal separation strongly supports the conclusion that the extracted high-temperature signal reflects genuine delayed phonon population dynamics rather than a residual Raman-matrix-element or electronic-susceptibility effect.

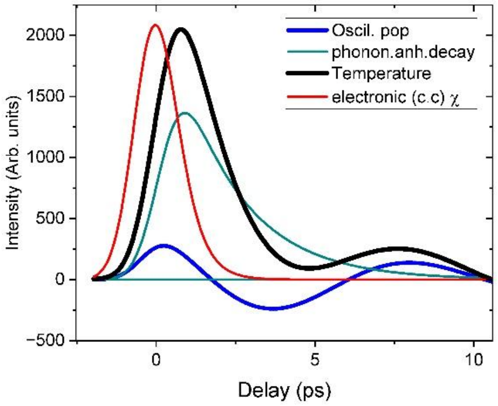

Figure R4: Simulated decomposition of electronic, phonon, and hybrid contributions to the transient Raman response. The modeled temporal components include the electronic Raman susceptibility χ(t) (red), the anharmonic phonon decay channel (cyan), the effective lattice temperature extracted from the phonon occupation (black), and the coherent hybrid-mode population (blue). The simulation highlights that the susceptibility response is confined to the earliest delay times, whereas the phonon temperature builds up later, demonstrating that the delayed high-temperature signal originates from phonon population dynamics rather than from the electronic Raman matrix element.

Finally, our main conclusion does not rely on the absolute value of the extracted temperature itself. The key result in Extended Data Fig. 3d is the trend that shows that, above the critical fluence Fc, the A_{1g}* mode reaches an extremely high temperature, however at this high effective temperature the folded phonon remains present. We acknowledge that the absolute uncertainty becomes larger when the anti-Stokes and Stokes intensities approach each other, but any marginal systematic correction common to all fluences would not change this central trend. We have clarified in the revised the Supplementary Information: In principle, subtle symmetry-related changes in the Raman matrix elements could renormalize the absolute temperature scale extracted from the Stokes/anti-Stokes ratio. We do not expect such effects to alter the central conclusion here for several reasons. A recent study of chiral CDW order in 1T-$TiSe_2$ suggests that possible chirality-related corrections are small in the present context, since our analysis is performed for the folded A_{1g}* phonon, which is the primary structural CDW mode rather than the secondary chiral refinement discussed in that work (Kim et al., Nat. Phys. 2024). Then, in our analysis we explicitly separate the phonon-population response from the transient Raman susceptibility: the susceptibility change is confined to the earliest delays, whereas the phonon occupation builds up later and exhibits the delayed behavior discussed in the main text. This temporal separation strongly supports the interpretation that the inferred temperature reflects phonon population dynamics rather than a transient renormalization of the Raman matrix element. Finally, our main conclusion does not rely on the exact absolute value of the extracted temperature, but on its fluence dependence: the folded A_{1g}* phonon remains clearly visible even when the inferred occupation becomes very large. We therefore use the Stokes/anti-Stokes ratio primarily as an effective measure of phonon occupation, while allowing that small symmetry-related corrections could renormalize the absolute temperature scale. .

7) minor issues / inconsistencies:

- Fig. 1, axis label in (c) should read "Temperature"

- Ni2TaSe5 should read Ta2NiSe5

- temperatures are sometimes referred to in the text as 300 K, yet labeled in the figures as 295 K.

- the statement "anti-Stokes TR-Raman, which more directly probes the phonon occupation" sounds a bit odd. Perhaps the authors meant to say that anti-Stokes TR-Raman allows for a more accurate determination of phonon occupation in the low-temperature limit?

Response: Fig. 1(c): corrected to “Temperature”.

Ni_2TaSe_5: corrected to Ta_2NiSe_5.

300 K versus 295 K: we have now made the notation consistent throughout the manuscript and figures.

We agree with the reviewer’s suggested wording and have revised the text accordingly. The sentence now states that anti-Stokes TR-Raman allows a more accurate determination of phonon occupation.

REVIEWER #2 (Remarks to the Author)

Many ultrafast experiments focus on 1-$TiSe_2$, widely regarded as a benchmark system for exploring nonequilibrium charge-density-wave and excitonic physics. While its band structure is relatively simple, complex many-body interactions create a wealth of intertwined excitation and relaxation channels. This complexity yields intriguing experimental findings, yet their interpretations frequently remain debated.

Thank you for the thoughtful assessment and for recognizing the high experimental standard and the quality of our dataset. We also appreciate that the reviewer acknowledges our efforts to test and rule out several non-relevant mechanisms. The additional alternative scenarios raised here are valuable, and we have evaluated each of them carefully. In the following and in the revised manuscript, we address the points systematically and show, using direct experimental results, that the proposed alternatives cannot account for our observations. Incorporating this deeper analysis has strengthened the clarity and robustness of the manuscript.

1) Recent ultrafast experiments using transient absorption spectroscopy [Huang et al., Nanotechnology 34, 235707 (2023)] and electron diffraction dynamics [Karam et al., ACS Photonics 5 1228–34 (2018)] on exfoliated 1T-$TiSe_2$ have revealed similar low-frequency oscillations, which arise from out-of-plane acoustic breathing modes and are attributed to the excitation of longitudinal acoustic phonons quantized by the sample thickness. For 14-nm thick 1T-$TiSe_2$, the transient absorption oscillation frequency is 0.147 THz, which is quantitively very close to the 0.13 THz modulation of the CDW A^*_{1g} mode observed by Hedayat et al., along with their 15 nm penetration depth estimate given in the supplementary information (Note 10). In this scenario, strong electron–phonon coupling in excited $TiSe_2$ is governed by Kohn anomalies, affecting electron transport and energy relaxation in the layered structure. The mode-selective nature of the Raman oscillations may relate to the specific character of the CDW mode, which emerges due to the acoustic branch folding (Fig. 1e).

Response: We fully agree that thickness-quantized out-of-plane acoustic breathing modes exist in thin flakes. However, assigning the ~0.13 THz modulation of the folded CDW A_{1g}* Raman mode observed in our bulk single crystal to a longitudinal acoustic phonon is incompatible with our experimental parameters and the underlying physics. We base this conclusion on four independent and mutually reinforcing reasons:

(1) Thickness quantization requires two free surfaces, optical penetration depth cannot replace a mechanical boundary.

A discrete breathing eigenmode is a standing longitudinal acoustic resonance defined by mechanical boundary conditions, specifically two free surfaces. In the reference that the Reviewer mentioned, Huang et al. explicitly demonstrate that optical penetration depth does not define this effective thickness: using

a 650 nm pump with a penetration depth of ~19 nm, their 52 nm flake oscillates at 0.040 THz. This is the frequency dictated by the full 52 nm physical thickness rather than the 19 nm excitation depth. Their 14 nm thick flake shows a 0.147 THz oscillation, again consistent with physical thickness scaling and not the penetration depth of ~19 nm . Therefore, in our thick macroscopic bulk sample, the optically pumped region is not related to a second free surface. Consequently, photoexcitation launches a propagating strain wavepacket into the bulk interior rather than forming a thickness-quantized standing resonance.

(2) Quantitative acoustic frequency scaling precludes a 0.13 THz geometric resonance in a bulk sample.

The breathing frequency in flakes scales as

$f = v / (2d)$,

where v is the longitudinal sound velocity and d is the flake thickness, a relationship utilized in the flake diffraction studies to extract thickness and elastic constants. Bulk neutron scattering measurements by Stirling et al. establish the out-of-plane longitudinal sound velocity in bulk $TiSe_2$ as v = 2740 +/- 100 m/s. Using this established bulk value, a fundamental breathing mode at 0.13 THz would strictly require a mechanically bounded slab of approximately 10.5 nm thickness with two free surfaces. Because our sample is a macroscopic bulk crystal, the geometry does not support a standing mode in this frequency range, directly contradicting the breathing mode interpretation for our ~0.13 THz signal.

(3) CDW soft-mode at M or L points, acoustic breathing modes, and Kohn-type anomalies.

The proposed argument mixes two distinct phenomena: CDW-related softening (driven by Kohn-type anomalies or exciton-phonon coupling) occurs at the finite wavevector q_{CDW} at the Brillouin zone boundary, whereas a breathing mode is a near-Gamma longitudinal acoustic phenomenon governed by small-q wavevector. This separation is explicitly maintained in the literature. In the paper cited by the Reviewer, Karam et al. attribute the fast biexponential diffraction-intensity dynamics to strong electron-phonon coupling associated with a Kohn anomaly for the lowest frequency mode at the Brillouin-zone boundary (CDW-soft mode). Conversely, they treat the coherent oscillations in peak positions as separate out-of-plane acoustic breathing modes originating from lowest-order LA phonons quantized by the thin-flake thickness. This distinction is reinforced by Otto et al., who demonstrate that key renormalizations pertain to the zone-boundary transverse soft branch (the CDW sector). Furthermore, bulk neutron scattering by Stirling et al. reports no evidence for a Kohn-type anomaly in the small-q acoustic dispersion regime, which is the precise regime relevant to the breathing-mode. Therefore, invoking zone-boundary CDW physics to explain a zone-center LA breathing mode is physically unwarranted.

(4) Our signal is observed strictly in the CDW phase, unlike broadband strain-induced breathing modes.

In thin-flakes, breathing mode oscillations manifest as broadband optical modulations in the transient dynamics and are observed at room temperature in the normal phase. By contrast, our ~0.13 THz oscillation is observed strictly in the CDW phase and modulates the folded CDW A_{1g}* Raman response. On the other hand, it is absent in the parent A_{1g} mode and in the electronic continuum. A macroscopic longitudinal acoustic (LA) breathing strain would not naturally produce such an exclusive, symmetry-related modulation of a single folded CDW Raman channel.

While the studies cited by the Reviewer illustrate thickness-quantized breathing oscillations in thin films, they simultaneously provide the physical constraints that preclude this mechanism in our bulk Raman observation: (i) optical penetration depth does not define mechanical quantization boundaries, (ii) scaling with the bulk sound velocity makes a 0.13 THz standing wave impossible in our sample, (iii) CDW/Kohn-anomaly physics is a zone-boundary phenomenon distinct from near-Gamma breathing acoustics, and (iv) our oscillation exhibits strict fluence, temperature and symmetry dependence, which is inconsistent with generic broadband strain-type modulations.

Should we add to the paper and to the reply letter a short comment stating that such acoustic breathing modes have been observed in thin films (citing also the papers) and discussing why they are incompatible with our observations.

2) In this context, impurities and lattice pinning can significantly influence interlayer coupling. Huang et al. (see Ref. above) note the inevitable presence of intrinsic intercalated Ti atoms in the van der Waals gap of 1T-$TiSe_2$ and propose that photoexcited electrons are rapidly trapped by these Ti defects, forming color centers (small polarons). They further propose that the density of intercalated Ti atoms (2.2 × 10^13 cm−2 per Se–Ti–Se layer) sets a threshold pump fluence. Under higher pump fluence, carrier congestion impedes the migration of excessive carriers. Could the photogenerated carrier density at Fc also be comparable to the Ti defect density?

Response: In the following we clarify further that the critical fluence we observed.

(1) The number intercalated Ti atoms (2.2×10^{13} cm^{-2} per Se–Ti–Se layer) that the reviewer mentioned is only valid for the sample in the cited paper [Huang et al., Nanotechnology 34, 235707 (2023)], it is not generic and we do not expect this number of defects in our sample. It is well established that self-intercalated Ti atoms act as electron donors that weaken excitonic correlations and suppress the equilibrium CDW transition temperature. Huang et al. report Tc ≈ 190 K, consistent with their high estimated defect density. Our bulk crystals exhibit a sharp Tc ≈ 202 K (SI, note 1), characteristic of highly stoichiometric, high-purity $TiSe_2$. Their "CVD grown" method is known for such density of defects and does not apply in our case. As we calculated in the SI, our density of photoexcitation at Fc (in the range of 2.5 - 3×10^{13} cm^{-2}) is in this order of magnitude. But our bulk crystalline sample has a substantially lower defect density than the defect-rich CVD grown flake. Thus, the numerical match suggested by the reviewer is completely accidental.

(2) Consistency with broader ultrafast $TiSe_2$ literature: One or two critical photoexcitation fluences have been observed in many previous ultrafast studies of $TiSe_2$ in relation to the dynamics of CDWs (not for defect-rich flakes as in the work of Huang et al). One of the earliest critical fluence measurements is referred in our abstract (Porer et al., Nat. Mater. 13, 857–861 (2014)). We now explicitly compare Fc to multiple prior studies on high-quality $TiSe_2$ reporting fluence thresholds for collapse of the electronic CDW excitonic or phononic order in the same regime, useful for showing that Fc is not related to defects or any other artifact, but this fluence scale is widely reported as a universal electronic or phononic instability threshold of CDW phase in high-quality samples rather than a sample-dependent defect saturation limit. To the response of this comment and specific comment of Reviewer 3 we now add a table which summarizes the critical fluences that have been found in other ultrafast experiments to the SI note 12 and to the end of this response letter.

3) The authors assess and argue against a thermally induced CDW collapse due to lattice overheating at pump fluences exceeding Fc. Their assessment in SI Note 10 shows that the corresponding accumulated energy is insufficient yet comparable to that of the CDW thermal transition, missing by a few times or merely tens of percent. In such close estimates, it is crucial to account for the spatial inhomogeneity of the excitation, both laterally and through the sample depth. Such spatial excitation inhomogeneity may indeed be the reason why the 0.13 THz mode vanishes at Fc, even though the A*1g phonon persists well beyond this threshold, rendering one of the authors' key arguments unconvincing.

Response: We thank the reviewer for this important point. We have carefully considered spatially inhomogeneous overheating, but we believe that it cannot explain our observations.

First, if the "melted fraction" picture were correct, the measured signal would simply be a spatial average of ordered and melted regions, $I_{total} = (1-\alpha)\, I_{ordered} + \alpha\, I_{melted}$, where α is the melted volume fraction. In that case, both the 0.13 THz oscillation and the A_{1g}* spectral weight would scale with the remaining ordered

volume, so their ratio should stay roughly constant as the ordered fraction shrinks. This is not what we observe. Instead, the 0.13 THz mode collapses at Fc while the folded A_{1g}* phonon clearly remains, which directly rules out a simple spatial inhomogeneity scenario.

Second, if the scenario described by the reviewer were correct and the probed region were merely heated above the equilibrium transition temperature, the folded A_{1g}* mode should disappear from the Raman spectrum, just as it does in equilibrium above T_{CDW}. Instead, A_{1g}* remains visible well above Fc, while only the 0.13 THz mode vanishes.

Third, the energy deposited by the pump is not sufficient for a purely thermal transition, as discussed in the SI. Based on the specific heat data, heating the sample to T_{CDW} requires about 55–60 meV per unit cell, whereas the deposited energy at that fluence is only about 37.4 meV per unit cell. Thus, the "tens of percent" difference mentioned by the reviewer is in fact substantial, here about 40%, and is not negligible. This is not just an arbitrary mismatch between calculation and experiment. A similar mismatch has been reported repeatedly in ultrafast studies of $TiSe_2$, beginning with the original work titled "Nonthermal Melting of a Charge Density Wave in $TiSe_2$" [Möhr-Vorobeva et al., Phys. Rev. Lett. 107, 036403 (2011)] , and is widely interpreted as evidence that the ultrafast transition is not driven by equilibrium lattice heating alone.

Fourth, in our data, the temperature dependence is opposite to what a thermally melted-fraction scenario would predict. In such a picture, increasing the base temperature should increase the melted fraction and strengthen the normal-phase response. Experimentally, we observe the opposite: the photoinduced A_{1g} intensity is strongest at low temperature and decreases as the base temperature is raised (Fig. 4 of the main manuscript). This is incompatible with simple thermal melting.

Finally, the Fc critical fluence itself falls in the regime where many independent ultrafast studies have reported pump fluences for suppression of the electronic, excitonic, or phononic components of the hybrid order. As suggested by Reviewer 3, we have collected these references and summarized them in a table in the SI. This strongly supports a loss of coherent hybrid order, rather than an effect from spatially inhomogeneous heating.

4) The authors note that the low-energy Raman continuum (LERC) may reflect excitonic fluctuations but avoid further discussion in this respect. It is worth citing another key reference alongside Refs 49 and 50: Kim et al., Nature Commun. 12, 1969 (2021). It remains ambiguous whether the authors consider the attribution of the LERC to exciton fluctuations unconvincing, or whether its behavior in their experiments conflicts with their own interpretation framework.

Response: We thank the reviewer for the reference and have now cited Kim et al. (Nat. Commun. 12, 1969 (2021)) alongside our existing references. We do not view an interpretation of the LERC in terms of excitonic fluctuations as conflicting with our framework. In fact, we find that the LERC is less pronounced at low fluences where electrons and holes remain largely condensed, suggesting that the continuum is linked to the breaking of the exciton condensate into excited carriers.

We nonetheless avoid a detailed microscopic interpretation in the main text because (i) its rise/decay are limited by our ~1.2 ps instrument response function, and we cannot provide more information than that the effect is in the picosecond timescale, comparable with electronic/excitonic response, and (ii) the full spectral line shape extends beyond our spectral window, therefore we cannot reveal the energy dependence of this effect and (iii) our central argument relies on the phononic response, which does not require a detailed assignment of the LERC. We therefore treat the LERC as a transient electronic-related background channel that we model and subtract to isolate phonon dynamics.

Now we made this explicit to remove ambiguity: Although the LERC may be related to the excitonic fluctuations (Kim et al., Nature Commun. 12, 1969 (2021)), given its nearly invariant lineshape across

conditions, we regard it as an electronic background channel (i.e., a change in the Raman susceptibility), and model it using an exponential function at each delay, which is then subtracted to provide the isolated phonon response (Supplementary Note 4).

5) Additional note: To prepare the article for future submission, authors should thoroughly edit the Supplementary Information to eliminate typos and inconsistencies: CDW-CWD, Fig.10a vs Fig.10(left), Jcm-1 vs. Jcm-2, the same "d" in "A" and "E_abs", etc.

Response: We thank the reviewer for the careful proofreading. We performed a careful SI edit and corrected all typos and inconsistencies (CDW/CWD, figure references, units, labels, and notation).

REVIEWER #3 (Remarks to the Author)

This manuscript reports a compelling study on phonon-exciton coupling in $TiSe_2$, employing the advanced experimental technique of time-resolved Raman spectroscopy. The comparison of Raman spectra pre- and post-optical pumping yields valuable insights into phonon excitation dynamics. While the work engages with intriguing physical phenomena, the interpretation would benefit from addressing several key points to solidify the conclusions.

We thank the reviewer for the constructive feedback and helpful suggestions.

1) For Figure 2, please clarify if the legend in panel (a) applies universally to panels (b) and (c). A similar clarification is needed for Figure 4a-d. Explicitly labeling the ΔI values in the relevant legends would also immediately enhance readability.

Response: We clarified all captions in the relevant panels to remove ambiguity.

2) The oscillatory decay of the A_{1g}* phonon intensity is attributed to coherent collective oscillations from phonon-exciton coupling. An alternative explanation could involve phonon-phonon scattering processes. Please discuss whether this mechanism was considered and, if feasible, comment on the possibility of incorporating it into the theoretical model to strengthen the argument for excitonic coupling.

Response: We thank the reviewer for this important point. We agree that phonon-phonon scattering contributes to the dynamics through the well-established signatures of decay, dephasing, and linewidth broadening of the A_{1g}* response. These effects are visible in our experimental data, including the time-resolved reflectivity and the supplementary analysis, and we now clarify this explicitly in the revised manuscript. In our model, such processes are included phenomenologically through the damping terms, namely the phonon damping γ in the lattice equation of motion and the relaxation term κ in the excitonic sector, which together account for relaxation into incoherent channels and the thermal background.

A purely lattice-based explanation would therefore require a distinct coherent phononic channel with the appropriate energy scale and fluence dependence. We find no evidence for such a mode in the known phonon dispersions or in our DFT calculations. By contrast, the observed phenomena are exactly what is expected if the oscillatory component is tied to the excitonic sector: the excitonic coherence is more fragile under photoexcitation than the periodic lattice distortion (PLD), so the collective mode can collapse at Fc while the folded structural phonon survives. For this reason, we interpret the oscillatory component as arising from coupled exciton-phonon dynamics, that is, the locked phason, whereas phonon-phonon scattering acts primarily as a decay and dephasing channel.

3) The identified critical fluence (Fc) is linked to a phase transition. How does this threshold compare with values reported in prior studies on $TiSe_2$ or analogous materials? Providing this context would help position the new findings within the existing literature.

Response: We thank the reviewer for this important point. We have now added a comparison table to the Supplementary Information; the table summarizes reported fluence thresholds for melting either the excitonic/electronic sector or the phononic/structural sector of the CDW in $TiSe_2$. We also revised the discussion to distinguish more clearly between critical fluences reported for electronic/excitonic observables and those reported for structural/PLD observables.

The reported Fc values are not expected to coincide exactly, because they depend on the base temperature, the observable being probed, whether incident or absorbed fluence is quoted, and the experimental geometry and probe sensitivity. With this distinction in mind, the literature shows a consistent hierarchy rather than contradictory thresholds: electronic or excitonic signatures generally collapse at lower fluence, whereas structural suppression occurs at higher fluence. Our Fc falls within this established range and is fully consistent with our central observation that the coherent hybrid mode disappears at Fc while the folded A_{1g}* phonon, and thus a substantial PLD, survives beyond it.

Reference	Technique	Observable used to define Fc	Fc1 (mJ cm−2)	Fc2 (mJ cm−2)	Remark
Rohwer et al., Nature 471, 490–493 (2011)	trARPES	Collapse of long-range charge order / spectral reconstruction	~0.1		No single sharp Fc explicitly defined low fluence series to melt the CDW gap
Möhr-Vorobeva et al., Phys. Rev. Lett. 107, 036403 (2011)	Time-resolved X-ray diffraction / optical reflectivity	Structural CDW melting / suppression of coherent A_{1g} CDW amplitude mode	0.17–0.21	0.35	Temperature-dependent structural threshold is 140 K
Porer et al., Nat. Mater. 13, 857–861 (2014)	Ultrabroadband THz spectroscopy	Electronic / excitonic suppression and high-fluence regime with persisting PLD	0.040	0.06–0.33	excitonic order collapses before PLD.<br>Minimum T is 150 K for PLD collapse
Hedayat et al., Phys. Rev. Research 1, 023029 (2019)	trARPES and tr-Reflectivity	Excitonic / electronic collapse and partial gap dynamics and optical response	0.06	~0.1- 0.2	Characteristic electronic threshold from TRR
Duan et al., Nature 595, 239–244 (2021)	tr-ARPES and Ultrafast electron diffraction	Exciton condensation quench / PLD suppression	0.073	0.135	two-threshold in gap dynamics of TR-ARPES
Burian et al., Phys. Rev. Research 3, 013128 (2021)	Resonant / nonresonant tr-XRD	Orbital / excitonic order in topmost layer and volume-averaged suppression	0.0123–0.033	~0.03–0.05	Directly resolves orbital/electronic sector separately from structural response
Cheng et al., Nat. Commun. 13, 963 (2022)	Ultrafast electron diffraction	Collapse of interlayer coherence / dimensional crossover	~0.05	~0.1	Characteristic threshold regime rather than one uniquely defined Fc
Huber et al., Sci. Adv. 10, eadl4481 (2024)	Ultrafast optical / photoemission study	Creation of light-induced semimetallic state	~0.1	—	Strong-drive electronic transition; not always reported as two sharp thresholds
Cheng et al., Nat. Phys. 20, 54–60 (2024)	Ultrafast electron diffraction	Topological defect formation / structural non-equilibrium regime	~0.1, ~0.16		approximate scale from reported fluence window
Kurtz et al., Nat. Mater. 23, 890–897 (2024)	Ultrafast electron diffraction	Full suppression of excitonic contribution to lattice distortion	~0.5		Full suppression reached only at much higher fluence
This work	TR-Raman + TR-reflectivity	Collapse of coherent hybrid mode while folded A_{1g}* persists	~0.1-0.2		Coherent hybrid mode disappears before full collapse of PLD

4) In Figure 3, the saturation of the anti-Stokes peak is suggested to correspond to a metastable state. Please elaborate on the proposed connection between saturation and metastability. Specifically, explain the physical rationale for why the phonon population would saturate under these conditions rather than continue to evolve.

Response: We thank the reviewer for this important question. Below Fc, the excitonic order sustains the coupled exciton-phonon potential and supports efficient population of the folded A_{1g}* mode. Above Fc, this hybrid coupling collapses: the PLD survives, but the folded phonon is no longer reinforced by the excitonic sector. As a result, additional absorbed energy does not directly drive A_{1g}* through the excitonic sector but is redirected into all other channels without preferred direction, including the electronic continuum, the emergent normal-phase A_{1g} mode, and incoherent relaxation, and the already highly populated A_{1g}* will not be the preferred channel anymore. This is why the A_{1g}* signal saturates. This interpretation is supported by the effective theory showing this saturation in Fig. 3g and illustrated schematically in Fig. 4f,g.

5) Significant phonon broadening is observed in Figures 3c and 3e, occurring after frequency stabilization. Could this broadening be attributed to enhanced phonon scattering? If so, please speculate on the potential scattering mechanisms (e.g., increased electron-phonon or defect-phonon scattering) that may become active in this regime.

Response: We appreciate the reviewer's insightful comment regarding the origin of the observed phonon broadening. We agree that the observed broadening is consistent with enhanced scattering and dephasing of the surviving A_{1g}* CDW phonon in the high-fluence regime. As the fluence approaches and exceeds F_c, the A_{1g}* mode remains clearly present, but it evolves in a strongly modified nonequilibrium environment in which the coherent hybrid response weakens while incoherent relaxation channels become more important. In this regime, the linewidth increase is most naturally interpreted as a signature of enhanced dephasing of the phonon response. A plausible contribution comes from the large transient phonon occupation, as indicated by the anti-Stokes response. Such a high occupation can enhance anharmonic decay processes [Balkanski et al., Phys. Rev. B 28, 1928 (1983)], while additional electronic damping from the nonequilibrium carrier background cannot be excluded. We prefer to keep this interpretation appropriately cautious, since the present data do not allow us to isolate a unique microscopic channel.

At the same time, the interpretation in terms of enhanced dephasing is supported independently by the time-resolved reflectivity results, where the damping time of the coherent A_{1g}* oscillation decreases markedly with increasing fluence, consistent with the broadening observed in the Raman response.

To clarify this point, we have added the following text to the Supplementary Information: At the same time, the mode broadens as the fluence approaches and exceeds F_c, consistent with enhanced dephasing in the high excitation regime. In addition, the damping time of the coherent A_{1g}* oscillation extracted from the time-resolved reflectivity data decreases markedly with increasing fluence (Supplementary Note 11, Fig. 14 e), consistent with the broadening of the A_{1g}* feature and indicating enhanced dephasing of the CDW phonon at higher fluence. This points to enhanced incoherent relaxation of the surviving A_{1g}* CDW phonon at higher fluence, most plausibly associated with the large transient phonon occupation, which can increase the phase space for anharmonic decay processes, with a possible additional contribution from electronic damping associated with the strongly excited nonequilibrium carrier background.

6) Figure 4d shows a less pronounced intensity change for the A_{1g} mode compared to the A_{1g}* mode. Does this imply a different thermal activity? Please discuss whether the A_{1g} phonon becomes more active with increasing temperature, or if the A_{1g}* phonon is simply less susceptible to thermal effects, and the implications for the coupling mechanism.

Response: We thank the reviewer for highlighting this important aspect of the data. We agree that the weaker temperature dependence of A_{1g}*, compared with the much stronger variation of the normal-phase A_{1g} mode, is an important result and provides further evidence that the high-fluence state is not simply thermal.

If the response were dominated by heating, both phonons would be expected to evolve toward their equilibrium temperature-dependent behavior. Instead, the photoinduced A_{1g} intensity decreases strongly as the base temperature is raised, while A_{1g}* remains comparatively robust. This does not imply that A_{1g} becomes more thermally active or that A_{1g}* is simply less sensitive to heating. Rather, it shows that the two modes are governed by different non-equilibrium channels. The A_{1g} response appears to be controlled by the transient electronic structure and its temperature-dependent relaxation pathways, likely linked to the progressive closing of the residual partial gap (this part was discussed in the main manuscript in detail), whereas the persistence of A_{1g}* indicates that a substantial PLD survives and its weak temperature dependence is consistent with a lattice distortion that is trapped in a non-thermal remnant state (we added this part to the revised manuscript). As the reviewer correctly notes, a purely thermal scenario would not produce such contrasting behavior between the two modes. We have now made this point more explicit in the revised discussion: The contrasting temperature dependences of A_{1g} and A_{1g}* therefore reflect distinct non-equilibrium channels rather than simple thermal activation of one mode over the other.

REVIEWER #4

We thank the reviewer for the co-review note and appreciate the time invested in evaluating our work.

Reviewer #1 (Remarks to the Author)

I am glad to see that the authors responded to all raised issues, sharing their detailed comments and explanations. Based on these responses together with the revisions of the main text and the supplementary I am now happy to recommend this manuscript for a publication in Nature Communications.

Response: We thank the reviewer for the positive evaluation of our response and for recommending publication of the manuscript in Nature Communications.

Reviewer #2 (Remarks to the Author):

In their response, the authors carefully addressed the reviewers' comments. Their detailed, point-by-point replies and the subsequent revisions further enhanced the manuscript's quality. In particular, they critically examined my comment regarding thickness-quantized out-of-plane acoustic breathing modes, as experimentally observed in thin $TiSe_2$ flakes — an effect that had been overlooked in their initial analysis. They also engaged with, and partly accepted, my comment regarding spatially inhomogeneous excitation and possible near-surface melting of the CDW in $TiSe_2$.

Despite the extensive list of counterarguments provided, I cannot agree with some of them and find remaining inconsistencies:

- The authors acknowledge that "in thin flakes, breathing mode oscillations manifest as broadband optical modulations in the transient dynamics and are observed at room temperature in the normal phase."

- Citing room-temperature neutron scattering measurements by Stirling et al., they estimate that "a fundamental breathing mode at 0.13 THz would strictly require a mechanically bounded slab of approximately 10.5 nm thickness with two free surfaces."

- However, thickness quantization does not necessarily require two mechanically exfoliated free surfaces — a sharp phase boundary can serve as the rear surface. The CDW phase is characterized not only by different structural periodicity but, more importantly, by dramatically different carrier scattering and density, CDW-induced gap features, and plasmon responses, all reflecting substantial band structure modifications.

In light of these points, might one not reasonably hypothesize that, under optical pumping within a very specific and narrow range of fluence, a sharp phase interface forms, and that a ~10 nm thick layer of the high-temperature phase accounts for the observed 0.13 THz modulation of the optical response?

Although I admit that this mechanism may sound exotic, I find the authors' proposed assignment of the 0.13 THz modulation to a locked phason no less exotic, particularly in light of possible phase coexistence arising from significant spatial inhomogeneity of both the pump and the probe. While this ambiguity complicates a definitive assessment of the manuscript, it highlights the unusual character and relevance of the experimental findings. I believe this work merits publication in some form. Whether it meets the

specific criteria of Nature Communications is ultimately a matter for the editors to decide, in consideration of the feedback from all reviewers.

Response: We thank the reviewer for the careful reassessment. We recognize that, in principle, a photoinduced phase boundary might be considered as a possible acoustic interface. However, the thin-flake studies on this material [Huang et al., Nanotechnology 34, 235707 (2023) and Karam et al., ACS Photonics 5 1228–34 (2018)] show a thickness-dependent breathing-mode frequency set by the physical flake thickness. This makes such a photoinduced acoustic interpretation much less straightforward for our bulk crystal. Nevertheless, because a more exotic phase-boundary scenario cannot be excluded on conceptual grounds alone, we have revised the Discussion to explicitly state that other exotic mechanisms cannot be fully ruled out: "Although this combined evidence strongly supports the locked-phason interpretation, other exotic mechanisms cannot be fully ruled out."

We retain the locked-phason interpretation because it provides the most consistent account of the complete set of observations. The assignment is not based solely on the absolute value of the 0.13 THz frequency, or on the corresponding acoustic length scale, but on the combined set of observations. Specifically, the modulation appears only in the ordered phase; selectively modulates the folded A_{1g}^* CDW phonon; softens toward the critical fluence in the same fluence range where non-thermal suppression of the CDW has been reported in prior ultrafast studies; and disappears at Fc while the A_{1g}^* phonon itself remains visible, indicating that the PLD survives. The anti-Stokes Raman data further show that the modulation is tied to the CDW phonon sector: it appears in the phonon-population dynamics, and the Stokes/anti-Stokes ratio reveals selective overheating and saturation of A_{1g}^* at the same critical fluence at which the locked phason vanishes. As explained in more detail in our previous responses and in the main manuscript, these observations are incompatible with a generic acoustic mode, an induced phase-boundary scenario, or a purely structural response alone, while they are well captured by the locked-phason scenario and our effective theory.

Reviewer #3 (Remarks to the Author):

All my previous concerns have been satisfactorily resolved. I support the publication of this work.

Response: We thank the reviewer for the positive evaluation and for supporting publication of the manuscript.

Reviewer #4 (Remarks to the Author):

I co-reviewed this manuscript with one of the reviewers who provided the listed reports. This is part of the Nature Communications initiative to facilitate training in peer review and to provide appropriate recognition for Early Career Researchers who co-review manuscripts.

Response: We thank the reviewer for the contribution to the peer-review process.
